# Rational design of thioamide peptides as selective inhibitors of cysteine protease cathepsin L†

**DOI:** 10.1039/d1sc00785h

**Published:** 2021-07-19

**Authors:** Hoang Anh T. Phan, Sam G. Giannakoulias, Taylor M. Barrett, Chunxiao Liu, E. James Petersson

**Affiliations:** Department of Chemistry, University of Pennsylvania Philadelphia Pennsylvania 19104 USA ejpetersson@sas.upenn.edu; Key Laboratory for Northern Urban Agriculture of Ministry of Agriculture and Rural Affairs, Beijing University of Agriculture Beijing 102206 P. R. China

## Abstract

Aberrant levels of cathepsin L (Cts L), a ubiquitously expressed endosomal cysteine protease, have been implicated in many diseases such as cancer and diabetes. Significantly, Cts L has been identified as a potential target for the treatment of COVID-19 due to its recently unveiled critical role in SARS-CoV-2 entry into the host cells. However, there are currently no clinically approved specific inhibitors of Cts L, as it is often challenging to obtain specificity against the many highly homologous cathepsin family cysteine proteases. Peptide-based agents are often promising protease inhibitors as they offer high selectivity and potency, but unfortunately are subject to degradation *in vivo*. Thioamide substitution, a single-atom O-to-S modification in the peptide backbone, has been shown to improve the proteolytic stability of peptides addressing this issue. Utilizing this approach, we demonstrate herein that good peptidyl substrates can be converted into sub-micromolar inhibitors of Cts L by a single thioamide substitution in the peptide backbone. We have designed and scanned several thioamide stabilized peptide scaffolds, in which one peptide, R^S^_1A_, was stabilized against proteolysis by all five cathepsins (Cts L, Cts V, Cts K, Cts S, and Cts B) while inhibiting Cts L with >25-fold specificity against the other cathepsins. We further showed that this stabilized R^S^_1A_ peptide could inhibit Cts L in human liver carcinoma lysates (IC_50_ = 19 μM). Our study demonstrates that one can rationally design a stabilized, specific peptidyl protease inhibitor by strategic placement of a thioamide and reaffirms the place of this single-atom modification in the toolbox of peptide-based rational drug design.

## Abstract 2

Information on the effects of sidechain and backbone modification on the activity of cathepsin (Cts) L, V, K, S, and B was used to design a thioamide peptide that is inert to all Cts and selectively inhibits Cts L.

## Graphical abstract

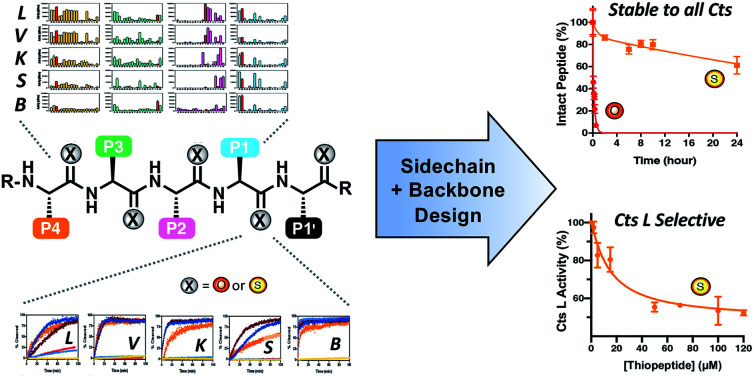

## Introduction

In recent decades, there has been an increased interest in the development of peptides as therapeutics and imaging agents.^1–3^ Peptide-based drugs offer advantages such as high selectivity and potency, low tissue accumulation, relatively predictable metabolism, and safety. Additionally, the ease of obtaining high biological and chemical diversity from standard synthetic procedures makes peptide therapeutics attractive.^1,4^ Peptides thus stand out as promising candidates to fill the gap between the two main drug categories – traditional small-molecule drugs (smaller than 500 Da) and biologics (larger than 5000 Da).^4^ Most of the peptides that are currently clinically approved or under active development are targeted for metabolic diseases and cancer.^2^ However, despite the numbers of known targets for peptide therapeutics and existing peptide libraries, peptides still display certain disadvantages that hinder them from more easily becoming effective drugs.^1,2^ Peptides are subject to rapid proteolysis, oxidation, display short half-lives and fast renal clearance *in vivo*, as well as low membrane permeability, thereby exhibiting suboptimal pharmacokinetics.^1,2^ To address the metabolic stability issues of peptides, modifications at protease cleavage sites using techniques such as synthetic substitutions of amino acid sidechains or the peptide backbone have been developed and utilized to increase resistance to proteolysis.^3,5–8^

Backbone thioamidation is a promising tool that has been shown to improve proteolytic stability of both linear and macrocyclic peptides.^9–20^ Our laboratory previously demonstrated that a thioamide substitution near the scissile bond of glucagon-like peptide-1 (GLP-1) and gastric inhibitory polypeptide (GIP), two therapeutically relevant peptides for diabetes treatment, significantly enhances their proteolytic stability against dipeptidyl peptidase 4.^21^ Thioamidation of GLP-1 and GIP increased their half-lives up to 750-fold without significantly compromising their cellular activity; the thioamide GLP-1 analogue was also biologically active in rats and exhibited improved potency for glycemic control compared to its native, all-amide GLP-1 counterpart.^21^ Motivated by these results showing thioamide stabilization effects at P2 and P1 positions (positions numbered from the scissile bond by convention), our laboratory developed a fluorescence sensor design to systematically study the positional effects of thioamide substitution against different cysteine proteases (papain, cathepsins L, V, K, B, and S) and serine proteases (trypsin, chymotrypsin, and kallikrein).^22–24^ Intriguingly, we found that thioamide positional effects differ not only between serine proteases and cysteine proteases, but also between members of the same protease family despite their high homology (31–59% sequence identity) and mechanistic similarity.^22,24^ We also successfully utilized data from these systematic studies to design a two-site stabilized thioamide peptide specifically targeting neuropeptide Y_1_-receptor expressing MCF-7 breast cancer cells.^22^ With the experimental data from these systematic studies, we recently developed a Rosetta machine learning model that accurately classifies positional effects of thioamides on proteolysis by these cysteine and serine proteases which can be used to rationally design stabilized peptides for therapeutic and imaging applications.^23^

Given this precedent, in this study, we aim to further utilize the strategic incorporation of thioamides to develop stabilized peptides as protease inhibitors, more specifically, inhibitors of the cysteine protease cathepsin L (Cts L). Among the 500–600 proteases identified in mouse and human, the cathepsin (Cts) family includes proteases that orchestrate numerous critical physiological processes and are involved in many different diseases such as neurological disorders, cardiovascular diseases, arthritis, obesity, and cancer.^25,26^ Cysteine Cts proteases, which comprise 11 members in humans (Cts B, C, F, H, K, L, O, S, V/L2, X, and W), belong to the papain-like cysteine protease family. They have been shown to be upregulated in many cancer types and play critical roles in cancer progression.^27,28^ Cts L is an ubiquitously expressed endopeptidase that is uniquely involved in the major histocompatibility complex (MHC) class II processing pathway,^29^ prohormone or proneuropeptide processing,^30–32^ and autophagy,^33^ as well as cardiac homeostasis and signal transduction.^34–37^ Cts L is highly expressed in tumors associated with breast cancer, colorectal cancer, and pancreatic adenocarcinoma.^27,38^ Cts L participates in the degradation of epithelial cadherins, transmembrane receptors, and extracellular domains of cell adhesion molecules in cancer cells, thereby disrupting cell adhesion, promoting tumor invasion, and possibly underlying resistance to chemotherapy.^27,39,40^ Importantly, Ou *et al.* recently showed that lysosomal activation of SARS-CoV-2's spike (S) glycoproteins by the host cell's Cts L, but not Cts B, is critical for its cellular entry *via* endocytosis during infection.^41^ These researchers showed that treatment with Cts L inhibitor SID 26681509 decreased SARS-CoV-2 pseudovirus entry into HEK 293/hACE2 cells by more than 76%, highlighting the role of Cts L in lysosomal priming of the virus upon entry.^41^ There is also evidence for elevated Cts L circulating level in COVID-19 patients.^42^ This is significant as Cts L inhibitors have now been identified as promising therapeutic agents to inhibit SARS-CoV-2 for potential treatment of COVID-19.^41–44^ It has been proposed that a protease inhibitor cocktail composed of a Cts L-specific inhibitor as well as serine protease inhibitors could be a safe and novel treatment for COVID-19 patients.^45^ Although it is desirable to develop Cts L inhibitors, there are currently no specific inhibitors for Cts L that have advanced to clinical trials as it is challenging to obtain selectivity against closely related Cts family members.^46^

Many of the known Cts L inhibitors, which are mostly small molecules, resemble its physiological substrate and often have electrophilic “warheads” (*e.g.* epoxide ring, acyloxymethyl ketone, aziridine, vinylsufonate, nitrile, or thiosemicarbazone) that are strategically placed to trap the catalytic Cys_25_ residue of Cts L.^46^ This follows a logic common to the development of covalent protease inhibitors, wherein a good protease substrate is converted into an inhibitor by strategic incorporation of such warheads.^46–48^ Inspired by this principle, we demonstrate herein that good peptidyl substrates of Cts L, designed by combining knowledge about substrate sequence specificity from previous positional scanning with our protease sensor studies, can be converted into good inhibitors of Cts L by a single thioamide substitution to the peptide backbone. Unlike the warhead strategy, thioamide modification only renders the substrate inert to proteolysis and does not result in covalent inhibition. There are many advantages to our strategy as concerns about the use of covalent enzyme inhibitors linger in spite of several successes with the aforementioned warhead approach.^46,49,50^ With our thioamidation approach, we hope to potentially overcome challenges with selectivity and off-target effects of Cts inhibitors that are normally encountered with small molecule protease inhibitors.^46^ An inhibitor with high specificity for a single Cts is a powerful tool compound for studying its role in health and disease and can serve as a therapeutic lead where such specificity is necessary to avoid undesirable side effects. In this study, we examine the stability of several thioamide peptide scaffolds toward Cts proteolysis and identify one peptide that shows resistance to Cts L, Cts V, Cts K, Cts S, and Cts B while inhibiting only Cts L. We also show that this stabilized thioamide peptide can inhibit Cts L in human hepatocellular liver carcinoma (HepG2) whole cell lysate. To our knowledge, this is the highest affinity thioamide-based protease inhibitor to date. Our studies show the potential of utilizing thioamides to stabilize and convert good peptidyl substrates into specific protease inhibitors.

## Results and discussion

### Designing and examining thioamide peptide inhibitors of cathepsin using a fluorescence protease sensor system

Previously, our laboratory has designed a fluorescent protease sensor system that capitalizes on the fluorophore quenching property of thioamides to monitor real-time protease activity.^22,24,51,52^ For our first-generation sensors, a thioamide and a fluorophore are placed on opposite sides of the scissile bond, thereby leading to a turn on of fluorescence upon cleavage.^51,52^ Building upon this design, we generated a series of peptides with 7-methoxycoumarin-4-yl-alanine (Mcm; μ) at both the N-terminus and C-terminus to systematically study thioamide positional effects on proteolysis of cysteine^24^ and serine^22^ protease substrates. Once the doubly-labeled peptide is cleaved, there will be a turn on in fluorescence as one of the fluorophores will be separated from the thioamide, regardless of the placement of the thioamide.^22,24^ This allows real-time monitoring of proteolysis kinetics. From our previous systematic studies with cysteine proteases (Cts V, Cts K, Cts S, Cts B, Cts L, and papain), we learned that thioamide substitution at the P1 position significantly slowed the proteolysis rates of the generic μLLKAAAμ substrate by Cts V, Cts K, Cts S, and Cts L, but not significantly by Cts B and papain.^23,24^ By convention, amino acids N-terminal to the scissile bond are denoted PX positions (*e.g.* P1, P2, P3; non-primed positions), while those C-terminal to the scissile bond are considered PX′ positions (*e.g.* P1′, P2′, P3′; primed positions). Interestingly, we found that the P1 thioamide peptide, μLLK^S^AAAμ (K^S^_P1_), not only showed the highest level of protease resistance to Cts L, but also served as a potent inhibitor of Cts L (*K*_I_ = 0.87 μM; Fig. S13 and Table S12†). Although this preliminary inhibition data was exciting, the K^S^_P1_ peptide would not be very stable *in vivo* since it could still be efficiently cleaved by other cysteine proteases (papain, Cts B, Cts V, Cts K, and Cts S)^23,24^ as the sequence of this peptide was designed to be generic. Motivated by these results, we thus envisioned advancing this approach to design and scan for a sequence-optimized, thioamide-containing peptide specific inhibitor to Cts L, yet being stabilized in the presence of other closely related cathepsins without inhibiting them.

**Fig. 1:**
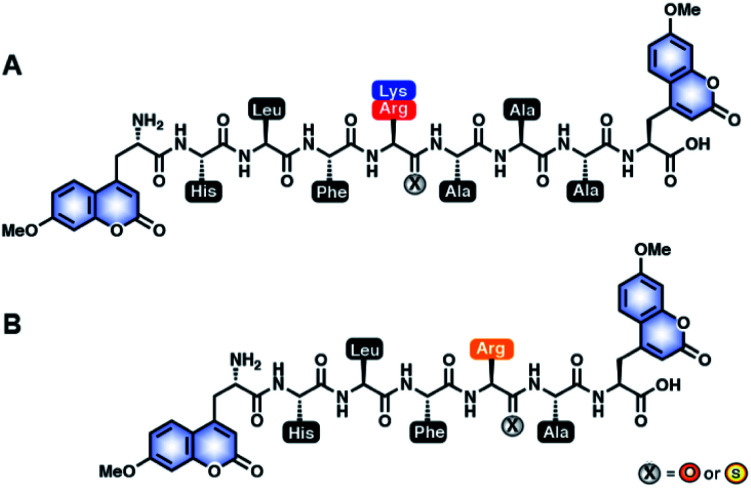
All-amide peptides and thioamide peptides investigated in this study. (A) Sequence-optimized all-amide and thioamide peptides of K_3A_ (μHLFKAAAμ) and R_3A_ (μHLFRAAAμ) for initial steady-state protease scanning with Cts L, Cts V, Cts K, Cts S, and Cts B and inhibition studies with Cts L. The peptides contain 7-methoxycoumarin-4-yl-alanine (MCM; μ) residues at both termini, and either an amide (X = O) or a thioamide (X = S) residue at the denoted P1 position. (B) Truncated all-amide and thioamide R1A peptide (μHLFRAμ). The R^S^_1A_ peptide, which shows stabilization against all five proteases, was further investigated for specificity and inhibitory effect in HepG2 whole cell lysate.

The rationale of our peptide design entails two main steps: (1) to design all-amide peptides that are good substrates of Cts L, then (2) to turn those substrates into stabilized peptides inhibiting Cts L by strategic placement of a single thioamide. In this study, utilizing the peptide sensor design from our previous studies, our peptide inhibitor candidates contained two coumarins (μ residues) at their termini, allowing for quick initial identification of peptides that showed resistance to proteolysis by cathepsins *via* steady-state protease assays (Fig. 1).^22,24^ To design the amino acid sequences for initial scanning, the primed positions of these peptides were kept generic and consistent with our previous studies by retaining alanine at the P1′, P2′, and P3′ positions. For the non-primed positions, sequence design was guided by a comprehensive substrate profiling study using a synthetic library of 160 000 fluorogenic tetrapeptides by Choe *et al.* (Fig. S1†).^53^ With the knowledge of different amino acid preferences by different cathepsins, we identified peptide sequences that might be specific to Cts L. At the P1 position, all human cathepsins prefer basic residues, so arginine and lysine were clearly the choice for this position, with arginine being preferred by Cts L.^53^ P2 is considered the major determinant for substrate specificity of Cts L that differentiates it from Cts K, Cts S, and Cts B, as Cts L has a unique preference for aromatic residues (phenylalanine, tryptophan, tyrosine) at this position.^53,54^ Similar preference for aromatic residues at P2 position is only observed in Cts V, which is most closely related to Cts L by sequence identity (78% sequence identity).^53^ As Cts V favors tryptophan and tyrosine over phenylalanine, phenylalanine seemed to be the best choice for P2. For P4, Cts L shows a preference for histidine, prompting us to choose histidine at this position. At P3, however, Cts L has less well defined specificity, but displays some preference for basic residues as well as a few aliphatic amino acids.^53^ Since we already included a positively charged residue at the P1 position, we decided to incorporate an aliphatic amino acid at P3 to reduce the potential for multiple Cts L cleavage sites. We chose leucine for the P3 position since the data from Choe *et al.* suggested that Cts L prefers leucine at P3 among the aliphatic amino acids (Fig. S1†).^53^ Regarding placement of the thioamide, the P1 position was chosen based on our previous systematic studies with the cysteine proteases that showed P1 thioamide peptide K^S^_P1_ gave the highest level of protease resistance.^23,24^ Using this rationale, the first series of peptides synthesized *via* solid-phase peptide synthesis for initial scanning with steady state protease assays were: μHLFRAAAμ (R_3A_), μHLFKAAAμ (K_3A_), and their P1 thioamide analogs (R^S^_3A_ and K^S^_3A_) (Fig. 1A). The thioamide position is denoted as a superscript “S” in the peptide sequences.

**Fig. 2:**
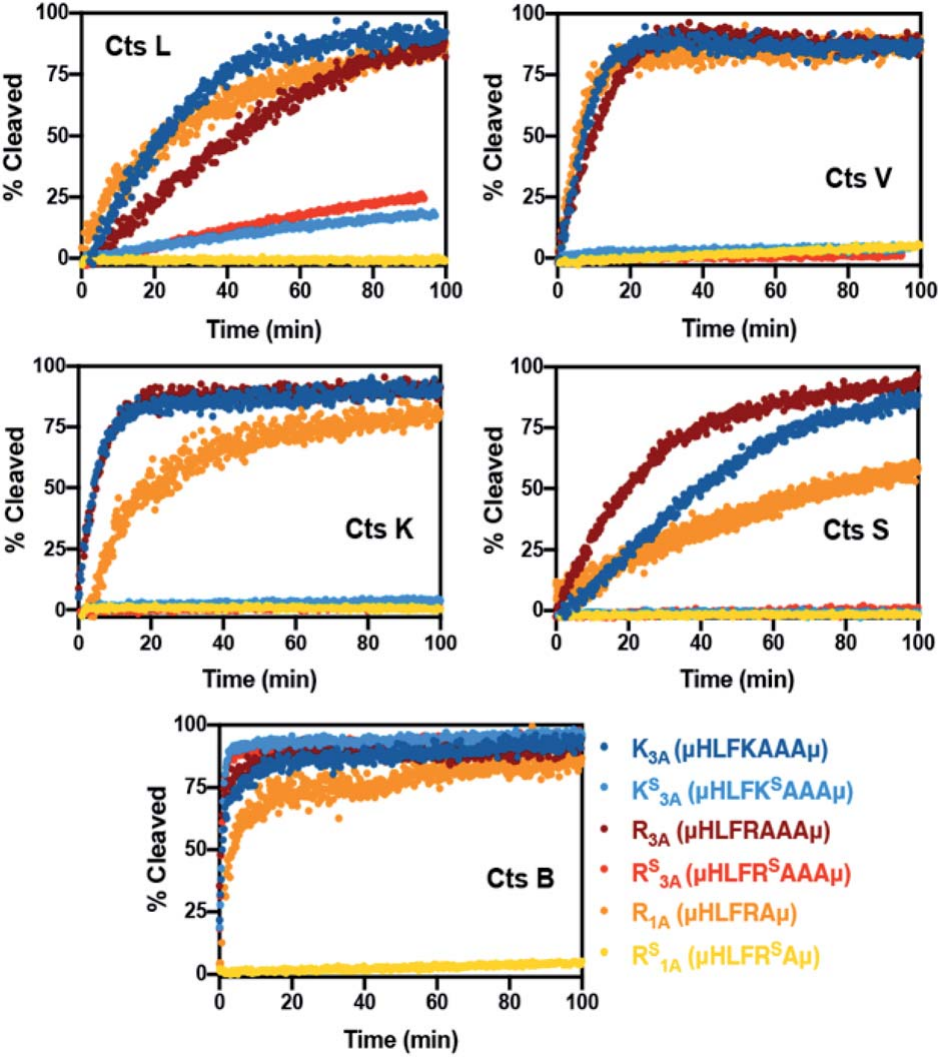
Summary of normalized cleavage data with cysteine cathepsin proteases. Peptides (7.5 μM) were incubated in the absence or presence of Cts B (37.6 nM), Cts K (42.6 nM), Cts L (30.3 nM), Cts S (21.6 nM) or Cts V (20.5 nM) in 100 mM sodium acetate, 100 mM NaCl, 1 mM EDTA, 5 mM DTT, and pH 5.5 at 27 °C. The original fluorescence was originally monitored at 390 nm with an excitation wavelength of 325 nm, then the fluorescence data was converted to percent cleavage rates. All traces are the average of three independent trials. Raw fluorescence data and more details of the assays are described in the ESI.†

To validate our design, we needed to first confirm whether the all-amide peptides were good substrates of the proteases before proving that the thioamide substitution could transform them into stabilized peptide inhibitors. For ease of comparison between thioamide positions and protease, raw fluorescence measurements were normalized and are presented in Fig. 2 (primary data are shown in ESI, Fig. S2–S6†). Initial rates of proteolysis were determined for each cleavage reaction (Table 1). High performance liquid chromatography (HPLC) and matrix-assisted laser desorption ionization mass spectrometry (MALDI MS) were used to confirm the cleavage sites in all assays (Tables S6–S11 and Fig. S7–S12;† cleavage sites summarized in Table S5†). In the absence of protease, no significant changes in fluorescence intensities nor degradation of the peptides in the assay buffers were observed. Both of the all-amide peptides K_3A_ and R_3A_ were recognized and efficiently cleaved by all five proteases, confirming that these were indeed good substrates of Cts L (Fig. 2 and Table 1). It is worth noting that the all-amide peptides K_3A_ and R_3A_ were both cleaved at the P1 position by all of the proteases, consistent with the fact that these five cysteine cathepsin proteases have high preferences for recognizing and cleaving their substrates at basic residues (Table S5†). Interestingly, for the all-amide peptides K_3A_ and R_3A_, cleavage sites other than the expected P1 position was also observed with Cts L, Cts V, and Cts K, while the peptides were cleaved at only the P1 position by Cts S and Cts B (Tables S6, S8 and Fig. S7, S9†). This likely reflects the fact that Cts L, Cts V, and Cts K are most closely related in sequence identity, resulting in similar preferences for substrate specificity. We then performed the assays with the thioamide analogs, where P1 thioamide stabilization was observed with Cts L, Cts V, Cts K, and Cts S (Fig. 2 and Table 1). This supports our choice of thioamide placement at the P1 position and is consistent with our previous findings reported in Liu *et al.* and Giannakoulias *et al.*, where we observed that P1 thioamides retarded proteolysis by Cts V, Cts K, Cts S, and Cts L, but not Cts B.^23,24^ Substituting the thioamide at the P1 position thus not only stabilized the P1 position, but also resulted in multiple-site stabilization effects in the cases with Cts L, Cts V, and Cts K (Tables S7, S9 and Fig. S8, S10†). We have previously observed similar multiple-site stabilization with thioamide substrates of serine proteases, which we were able to exploit to stabilize cancer cell imaging peptides at two positions with a single thioamide modification.^22^ Overall, having a thioamide at the P1 position here rendered the K^S^_3A_ and R^S^_3A_ peptides completely resistant to proteolysis by Cts V, Cts K, and Cts S, while significantly slowing the rate of proteolysis by Cts L (Table 1). The only exception to this P1 thioamide effect was with Cts B, where the K^S^_3A_ and R^S^_3A_ peptides were cleaved at the penultimate C-terminal alanine residue as indicated by the slashes – μHLFK^S^AA/Aμ or μHLFR^S^AA/Aμ (Tables S7, S9 and Fig. S8, S10†). This pattern of cleavage by Cts B aligns well with the fact that Cts B is known to be both an endopeptidase and a carboxydipeptidase (exopeptidase).^28^ The K^S^_3A_ and R^S^_3A_ peptides were also slowly cleaved by Cts L at the same position. We therefore postulated that a truncated version of this peptide, μHLFR^S^Aμ (R^S^_1A_; Fig. 1B), would eliminate this cleavage site by Cts L and Cts B and stabilize the peptide. As expected, the all-amide version of this shorter peptide (R_1A_; μHLFRAμ) was recognized and cleaved by all five cathepsins – Cts L, Cts V, Cts K, Cts S, and Cts B (Fig. 2, S11 and Tables 1, S10) while the corresponding thiopeptide R^S^_1A_ was left intact (Fig. 2, S12 and Tables 1, S11). Since preceding literature and our inhibition assays with the K_3A_ and R_3A_ peptides suggested that the arginine substrates have higher affinity for Cts L, we proceeded to investigate in depth the R^S^_1A_ peptide instead of its lysine analog (μHLFK^S^Aμ) as discussed in the next section.^53^

**Table 1:** Initial rates^a^ of peptides cleavage by cysteine cathepsin proteases

Peptide	Sequence	Cts L	Cts V	Cts K	Cts S	Cts B
K_3A_	μHLFKAAAμ	0.181 ± 0.003	0.511 ± 0.025	0.689 ± 0.045	0.110 ± 0.002	1.921 ± 0.206
K^S^_3A_	μHLFK^S^AAAμ	0.014 ± 0.000	0.002 ± 0.000^b^	0.002 ± 0.000^b^	0.000 ± 0.000^b^	3.643 ± 0.153
R_3A_	μHLFRAAAμ	0.112 ± 0.003	0.363 ± 0.014	0.564 ± 0.024	0.189 ± 0.004	2.747 ± 0.265
R^S^_3A_	μHLFR^S^AAAμ	0.023 ± 0.000	0.002 ± 0.000^b^	0.001 ± 0.000^b^	0.002 ± 0.000^b^	3.968 ± 0.209
R_1A_	μHLFRAμ	0.125 ± 0.003	0.553 ± 0.021	0.402 ± 0.021	0.053 ± 0.000	1.034 ± 0.074
R^S^_1A_	μHLFR^S^Aμ	0.000 ± 0.000^b^	0.005 ± 0.000^b^	0.000 ± 0.000^b^	0.000 ± 0.000^b^	0.003 ± 0.000^b^

a All rates are reported in μM min^−1^. Rates and standard errors are calculated by fitting to linear regression function in Prism 8.

b MALDI MS and HPLC confirmed essentially no cleavage with these peptides. Details are reported in the ESI.

### Investigating inhibitory effects with cathepsin proteases

Inhibition assays with Cts L were performed with all of the peptides from the first series (K_3A_ and R_3A_ peptides and their P1 thiopeptides) as well as the R^S^_1A_ peptide (Tables 2, S13–S18 and Fig. S14–S19†). For these assays, Z-Phe-Arg-AMC (Z-FR-AMC, where Z is benzyl and AMC is 7-aminomethylcoumarin), which is a commercial fluorogenic substrate of Cts L, was used. As the Z-FR-AMC substrate was cleaved by Cts L, its turn-on fluorescence was monitored at 460 nm with an excitation wavelength of 380 nm, which is different from the wavelength used for monitoring potential cleavages of our peptides with the μ residues (*λ*_excitation_ = 325 nm; *λ*_emission_ = 390 nm), allowing them to be separately monitored without interference. The all-amide peptides (K_3A_ and R_3A_) showed some inhibitory effects, which was expected since these substrates compete for the active site of Cts L (Table 2). The corresponding thioamide peptides, K^S^_3A_ and R^S^_3A_, were very good inhibitors of Cts L, with respective *K*_I_ values of 0.60 ± 0.15 μM and 0.52 ± 0.12 μM (Table 2). The R^S^_1A_ peptide (μHLFR^S^Aμ), which showed resistance to proteolysis by all five cathepsins, was also a good inhibitor of Cts L with a *K*_I_ value of 1.11 ± 0.22 μM. Although the R^S^_3A_ and K^S^_3A_ exerted slightly better inhibitory effects than the truncated peptide R^S^_1A_, they are not as ideal because we established in the steady-state protease assays that they could be cleaved by Cts L and by Cts B at the C-terminus. Lastly, the role of the two coumarins was examined with the coumarin-free peptide HLFR^S^A (R^S^_1A_^*^). Although this peptide showed resistance to cleavage by Cts L (Fig. S20†), it was a significantly weaker inhibitor of Cts L (*K*_I_ = 13.23 ± 6.89 μM), indicating an important role for the coumarins in binding. The finding that the thioamide peptides could serve as potent inhibitors of Cts L was exciting because our previous investigations of thioamide-stabilized protease substrates had found them to be only fairly weak inhibitors, implying that the thioamide primarily acted to disrupt binding to the protease.^21,22,24^ Indeed, earlier investigations of thioamide effects on proteolysis had found similar results for di- and tripeptides.^14,15,20,55^ Thus, we wished to further investigate the mechanism of inhibition.

**Table 2:** Evaluation of Cts L inhibition by the all-amide and thioamide peptides^a^

Peptide	*K*_I_ (μM)	*α*	*αK*_I_ or *K*′_I_ (μM)
K_3A_	3.05 ± 0.64	1.91 ± 0.92	5.81 ± 3.06
K^S^_3A_	0.60 ± 0.15	2.18 ± 1.35	1.30 ± 0.87
R_3A_	1.68 ± 0.49	1.33 ± 0.78	2.23 ± 1.47
R^S^_3A_	0.52 ± 0.12	1.83 ± 0.99	0.95 ± 0.56
R^S^_1A_	1.11 ± 0.22	1.61 ± 0.69	1.79 ± 0.85
R^S^_1A_^*^	13.23 ± 6.89	1.71 ± 1.89	22.58 ± 27.61

a Data was obtained by fitting to the mixed inhibition model that allows us to simultaneously determine the *K*_I_ and the mechanism of inhibition using the output “alpha” (*α*) in GraphPad Prism 8 software.^56,57^ Detailed analysis are described in the ESI.

**Fig. 3:**
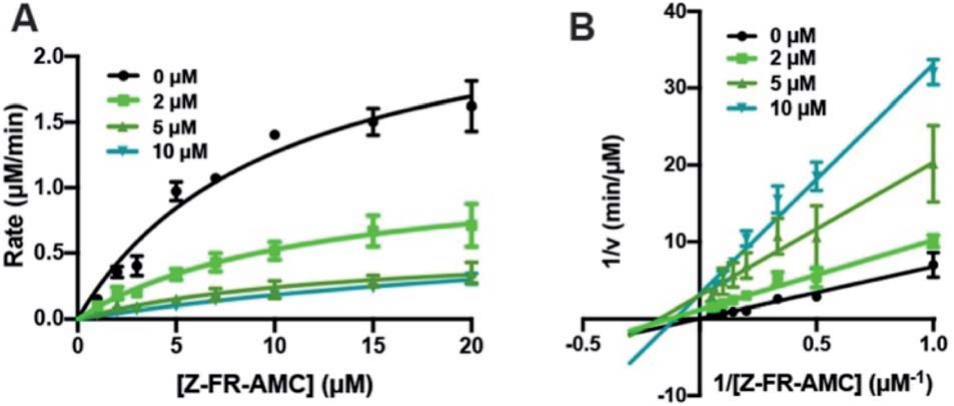
Cts L proteolysis inhibition by R^S^_1A_ peptide (μHLFR^S^Aμ). (A) Michaelis–Menten analysis and (B) Lineweaver–Burke plot of Cts L activity in the absence of presence of three different concentrations of the R^S^_1A_ peptide (μHLFR^S^Aμ). Various concentrations of the fluorogenic Z-Phe-Arg-AMC (Z-FR-AMC) were incubated in the presence of 37.93 nM Cts L. Averages of three trials along with the standard deviations are shown. Raw fluorescence traces are reported in Fig. S18.† These suggest that the peptide is likely a mixed-type inhibitor, with element of competitive inhibition since a value of *α* = 1.61 (*α* > 1) suggests tighter binding to the free enzyme.

From initial evaluation of the kinetic parameters obtained by fitting data to a Michaelis–Menten model (details are shown in the ESI; Tables S13–S18†), there was generally a decrease in *V*_max_, but either a minor increase or no significant change in *K*_M_ as the concentration of the inhibitors was increased. This eliminates the possibility of these peptides as acting as purely competitive inhibitors or uncompetitive inhibitors, suggesting that they are likely mixed-type inhibitors of Cts L based on the traditional categorization of inhibitors. This can be easily visualized in Lineweaver–Burke plots (Fig. S14–S19†), confirming the high likelihood of a mixed-type mechanism of inhibition, as shown in Fig. 3 for the R^S^_1A_ peptide. To further evaluate the mechanism of inhibition and to obtain the *K*_I_ values, kinetics data were fitted using a non-linear regression analysis with the mixed inhibition model that allows us to determine the *K*_I_ and the mechanism of inhibition using the output “alpha” (*α*) using GraphPad Prism software.^56^ All of the *α* values are consistently between 1–2, confirming these peptides are likely mixed-type inhibitors, with element of competitive inhibition since a value of *α* > 1 suggests tighter binding to the free enzyme (Table 2).^57^ Interestingly, mixed inhibitors of Cts L have been previously shown to be promising antiviral candidates. A study with a high-throughput screening of 5000 molecules discovered a small-molecule inhibitor of Cts L (5705213) with a mixed inhibition mechanism that can inhibit Cts L-mediated cleavage of the viral glycoproteins derived from all four viruses – SARS-CoV, Ebola, Hendra, and Nipah viruses, a process that is essential for entry into host cells.^58^

To serve as useful specific inhibitors of Cts L, the thioamide peptides must also be inert to cleavage by other proteases that may be present *in vivo* while not inhibiting them. Since the sequence-optimized R^S^_1A_ peptide herein showed resistance to proteolysis by all five proteases, we then assessed the specificity of inhibition by the R^S^_1A_ peptide by determining whether it could also effectively inhibit Cts V, Cts K, Cts S, and Cts B using assays similar to the Z-FR-AMC used with Cts L. The R^S^_1A_ peptide exhibited a 26-fold increase in *K*_I_ and is a weak mixed-inhibitor of Cts V, with a *K*_I_ of 26.22 ± 8.42 μM (Fig. S21 and Table S19†). No significant differences in the values of *k*_cat_ and *K*_M_ were observed for proteolysis of Z-Leu-Arg-AMC (Z-LR-AMC) by Cts K or Cts S in the presence of >30 μM concentrations of R^S^_1A_ peptide (Fig. S22, S23 and Tables S20, S21†). Similarly, essentially no differences in *k*_cat_ and *K*_M_ were observed for Cts B proteolysis of the Z-Arg-Arg-AMC (Z-RR-AMC) substrate in the presence of up to 50 μM R^S^_1A_ peptide with Cts B (Fig. S24 and Table S22†). In summary, in addition to acting as a potent inhibitor of Cts L (*K*_I_ = 1.11 ± 0.22 μM), the R^S^_1A_ peptide is ≥25-fold selective against other Cts family members tested, as it only weakly inhibits the closely related Cts V (78% sequence identity with Cts L) and shows little to no significant inhibitory effects with Cts K, Cts S, and Cts B (58%, 55%, and 26% respective sequence identity with Cts L).

### Evaluation of cathepsin L inhibition in HepG2 whole cell lysate

**Fig. 4:**
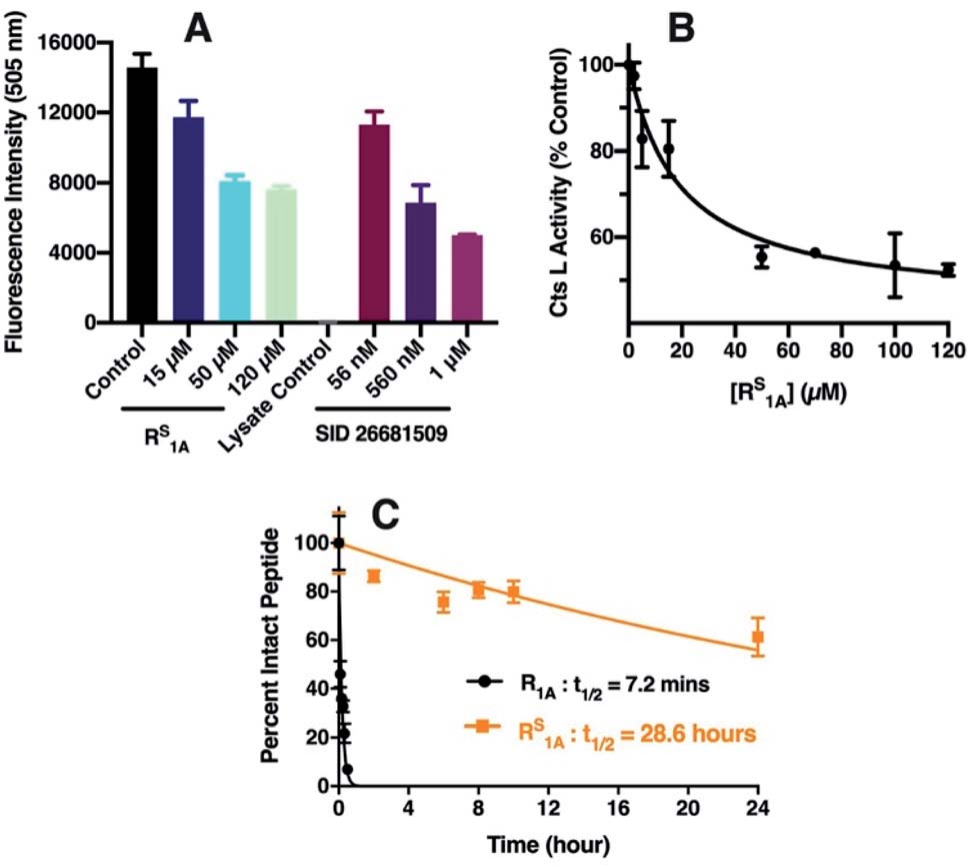
Inhibition of Cts L activity by R^S^_1A_ peptide in HepG2 whole cell lysate. (A) Cts L activity in HepG2 cell lysate monitored by fluorescence intensity at 505 nm. The control (black bar) was done without the inhibitor. The colored bars show fluorescence signals in the presence of select R^S^_1A_ peptide concentrations. SID 26681509, a known Cts L inhibitor,^59,60^ was used as the positive control. The lysate control was a background control for any inherent fluorescence signals from the cell lysate. (B) Cts L activity at different inhibitor concentrations calculated by taking the % of the average fluorescence signal from the control (without inhibitor). Error bars represent the standard deviations from three trials. The IC_50_ value was obtained from fitting to a sigmoidal dose–response equation in GraphPad Prism 8 (details of fitting are in the ESI†). (C) Stability of the all-amide peptide (R_1A_) and thioamide-peptide R^S^_1A_ in HepG2 lysate as monitored by HPLC.

Cts L has been considered an appealing target for cancer treatment because its expression has been linked to tumor progression and metastases of different types of cancers.^38,61^ In particular, it has been previously shown that increased Cts L expression is associated with worse outcome in hepatocellular carcinoma patients^62^ and elevated Cts L activity has been found in malignant liver cancer HepG2 cells.^63^ To further validate our R^S^_1A_ peptide inhibitor of Cts L, we investigated whether it could effectively inhibit Cts L activity in whole cell lysate from the HepG2 human hepatocellular liver carcinoma cell line. Using a commercially available fluorescence based Cts L activity kit, we incubated different doses of R^S^_1A_ peptide with HepG2 whole cell lysate. We found that the R^S^_1A_ peptide could effectively inhibit fluorescent reporter activity in the HepG2 whole cell lysate (IC_50_ = 19.3 ± 4.5 μM) (Fig. 4). Significantly, MALDI MS and HPLC data showed that this peptide's half-life was 28.6 hours, which was approximately 238 times more stable than its all-amide counterpart, R_1A_, with a half-life of only 7.2 minutes in HepG2 whole cell lysate (Fig. 4C and S26†). The fact that this thioamide peptide showed great stability in the presence of other proteases and cellular components in the HepG2 whole cell lysate further corroborated the enhanced stability we previously observed in steady-state protease assays with individual cathepsins (Cts L, V, K, S, and B). Excitingly, our preliminary data showed that R^S^_1A_ peptide could also inhibit Cts L in human MDA-MB-231 breast cancer cells overexpressing Cts L (Fig. S47†).^42,64^ These findings establish exciting precedent for translating R^S^_1A_ to *in vivo* assays to determine the impact of highly specific Cts L inhibition on processes such as cancer cell growth and viral uptake.

### Computational modeling

**Fig. 5:**
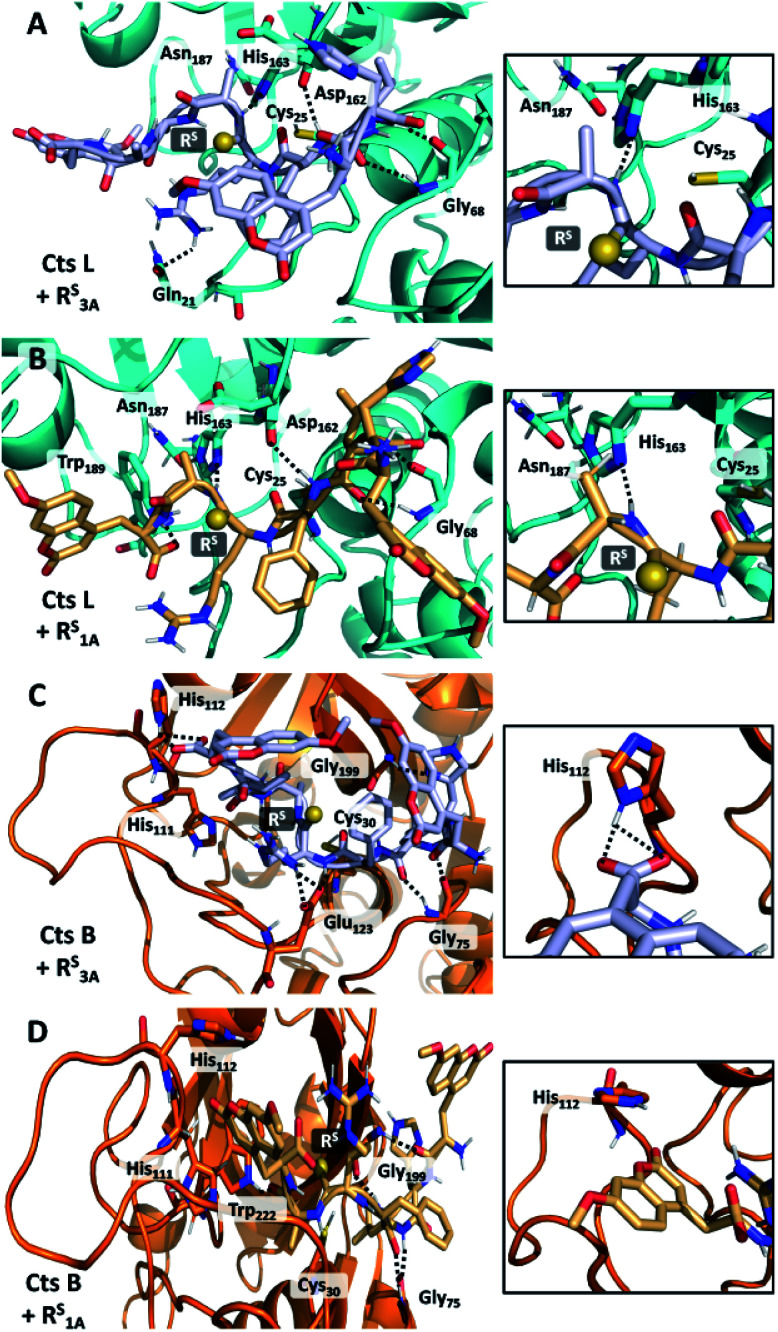
Interactions of R^S^_3A_ and R^S^_1A_ peptides in cathepsin L and cathepsin B active sites. (A and B) Docked structures of R^S^_3A_ (A) and R^S^_1A_ (B) with Cts L. The N–H group of Ala of either peptide forms hydrogen bond with His_163_ of the Cts L's catalytic triad. (C) Structure shows interaction of the C-terminal μ of R^S^3A peptide with His_112_ on the occluding loop of Cts B, allowing the peptide to be cleaved in a carboxydipeptidase manner and may explain why this peptide is not stabilized against Cts B. (D) The truncated peptide R^S^_1A_ no longer possess this interaction with His_112_, which may protect it against proteolysis by Cts B in addition to other proteases.

In order to rationalize the specific inhibitory effects of our peptides, we utilized computational modeling to flexibly dock the longer peptide R^S^_3A_ and the truncated peptide R^S^_1A_ with Cts L and the other four cathepsins investigated in this project. Interestingly, exclusively in the Cts L simulations, we observe that the P1 thioamide bond N–H of the R^S^_3A_ peptide can interact with His_163_, which is part of the Cts L catalytic triad (Fig. 5A). Hydrogen bonding in this manner would prevent His_163_ from efficiently deprotonating Cys_25_, thereby attenuating the proteolytic activity of Cts L and making the R^S^_3A_ peptide a good inhibitor. Similarly, with the truncated peptide R^S^_1A_, only with Cts L, did we observe the interaction between the P1 thioamide N–H group of the peptide and His_163_ (Fig. 5B). This hydrogen bond would be expected to be stronger for the thioamide than for the amide.^5,65^ This may explain why both the R^S^_3A_ and R^S^_1A_ peptides can effectively inhibit Cts L.

Our computational modeling can also be used to reasonably explain our other experimental data. From the steady-state protease assays, we found that the only exception to the P1 thioamide stabilization effect was with Cts B, where the R^S^_3A_ peptide was cleaved at the last two C-terminal alanine residues (μHLFR^S^AA/Aμ), which is consistent with the fact that Cts B is both an endopeptidase and a carboxydipeptidase.^28^ Upon examination of the docked structure of the R^S^_3A_ and Cts B, we found that the carboxylic acid of the C-terminal μ of the peptide interacts with His_112_ on the occluding loop, which is one of the two histidines (His_111_/His_112_ or His_110_/His_111_) known in the literature to anchor the C-terminal carboxylate of substrates to give Cts B its carboxydipeptidase properties (Fig. 5C).^66,67^ The truncated peptide R^S^_1A_ eliminates this interaction, thus protecting the peptide from proteolysis by Cts B and making it inert to all five cathepsins L, V, K, S, and B while specifically inhibiting Cts L (Fig. 5D).

**Fig. 6:**
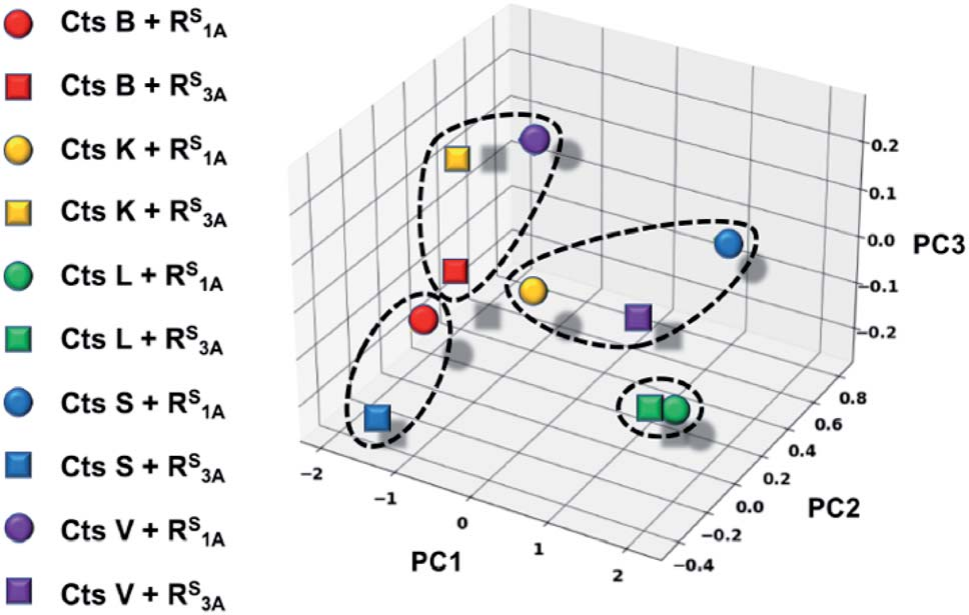
Three-dimensional plot displaying energetic clustering of the cathepsin–peptide complexes. The *x*, *y*, and *z* axes represent the condensed energy vectors of the complexes from principle component analysis (PC1, PC2, PC3). The solid colored shapes correspond to each of the ten protease–peptide complexes simulated in this study, as indicated. The dotted lines surrounding data points indicate the four clusters, including one that comprises the two Cts L complexes.

In an effort to further rationalize why incorporation of a thioamide at the P1 position imbues inhibitory effects for both the longer peptide R^S^_3A_ and shorter peptide R^S^_1A_ with Cts L and not the other cathepsins, we performed the following two analyses. The first analysis investigated the change in distances observed between the active site cysteine sulfur and the scissile bond carbonyl carbon upon incorporation of the thioamide. We detected large increases of up to 1.2 Å in this distance (placing the active site residue outside the range for nucleophilic attack) for the two Cts L peptides of interest (Table S25†). Importantly, despite this change in backbone geometry, the key histidine hydrogen bonding interactions were preserved. Our second retrospective analysis utilized unsupervised machine learning (KMeans Clustering) of residue-level energy differences between amide and thioamide peptide complexes from our structural models. Energy feature clustering analysis demonstrated that the Cts L peptides were clustered with each other, but separately from all other clusters (Fig. 6). These data indicate that the changes in energy associated with thioamidation in Cts L complexes are distinct when compared with thioamidation energy changes for the other cathepsins. Taken together, our identification of relevant hydrogen bonding interactions, tolerance of the complexes to the incorporation of a P1 thioamide (change in distance upon removal of constraints), and energy feature clustering identify distinct aspects of the R^S^_3A_ and R^S^_1A_ complexes with Cts L that can explain the mechanism of their specific inhibition: thioamidation disrupts binding of the peptides to other proteases while it strengthens a hydrogen bonding interaction with Cts L that keeps R^S^_3A_ or R^S^_1A_ tightly bound.

## Conclusions

In summary, we have examined several thioamide peptide scaffolds and identified one peptide, R^S^_1A_, that is not only resistant to proteolysis by all five cathepsins (Cts L, Cts V, Cts K, Cts S, and Cts B), but is also a potent, specific inhibitor of Cts L. This peptide can reversibly inhibit Cts L without degradation in HepG2 liver cancer cell lysate and shows promising activity in MDA-MB-231 breast cancer cells. Such a peptide is desirable since peptide-based agents, especially those targeting proteases, are often subject to degradation *in vivo*. Furthermore, reversible inhibitors like this could potentially address the safety concerns from lack of specificity and potential elicitation of immune responses with irreversible, covalent inhibitors.^49,50^ While the selectivity against other cathepsins is not as high as some previously reported peptidomimetics (primarily covalent inhibitors),^45^ this has not been our focus here. Rather, we sought to demonstrate that one can rationally design a potent reversible protease inhibitor by strategic modification of amino acid sidechains and thioamide position based on sensor data from our own work and others. More detailed mechanistic studies, as well as further optimization of this peptide for higher affinity and selectivity will be pursued and reported subsequently. Our studies show the potential of utilizing thioamides as stabilized peptide inhibitors and reaffirm the value of thioamides in the peptide drug design toolbox. In future studies, we will further optimize the thioamide peptide scaffolds by exploring substitutions with unnatural amino acids as well as more carefully examining the role of the N- and C-terminal coumarin groups, removal of which led to a 13-fold decrease in *K*_I_. More rigorous biological studies, including assessment of cell permeability, are also warranted to more fully assess the utility of these compounds for *in vivo* studies of Cts L and possible therapeutic advancement. Given our previous success in machine learning approach to predict thioamide effects and the existing database of sequence effects on cathepsin activity, we may be able to computationally design peptide-based inhibitors for cathepsins as well as for other targets.^23^ Taken together these approaches can form a paradigm for developing thioamide-stabilized peptides as enzyme inhibitors.

## Experimental

### Protease assays with sensor peptides

For a typical trial, a 7.5 μM peptide solution was incubated in the absence or presence of the appropriate concentration of Cts L (30.3 nM), Cts V (20.5 nM), Cts K (42.6 nM), Cts S (21.6 nM), or Cts B (37.6 nM) in 100 mM sodium acetate, 100 mM NaCl, 1 mM EDTA, 5 mM DTT, and pH 5.5 buffer at 27 °C. The fluorescence was monitored as a function of time at 390 nm with an excitation wavelength of 325 nm using a Tecan M1000 plate reader. Three independent trials were conducted for each assay to ensure reproducibility. More details of the assays, along with the raw data and analysis, are included in the ESI.†

### Inhibition assays with cathepsins L, V, K, S, and B

For Cts L, various concentrations of its substrate Z-Phe-Arg-AMC (Z-FR-AMC; 1 μM, 2 μM, 3 μM, 5 μM, 7 μM, 10 μM, 15 μM, and 20 μM) were reacted with 37.93 nM Cts L. For Cts V, different concentrations of its substrate Z-Leu-Arg-AMC (Z-LR-AMC; 1 μM, 3 μM, 5 μM, 7 μM, 10 μM, 15 μM, 20 μM, and 25 μM) were reacted with 19.3 nM Cts V. For Cts K, different concentrations of the substrate Z-LR-AMC (10 μM, 15 μM, 20 μM, 30 μM, 40 μM, 60 μM, 80 μM, and 100 μM) were reacted with 53.2 nM Cts K. For Cts S, various concentrations of the substrate Z-LR-AMC (15 μM, 20 μM, 25 μM, 30 μM, 40 μM, 60 μM, 80 μM, and 100 μM) were reacted with 33.8 nM Cts S. Lastly, for Cts B, various concentrations of the substrate Z-Arg-Arg-AMC (Z-RR-AMC; 40 μM, 100 μM, 200 μM, 400 μM, 600 μM, 800 μM, 1000 μM, and 1200 μM) were reacted with 40.9 nM Cts B. All assays were performed in an assay buffer consisted of 100 mM sodium acetate, 100 mM NaCl, 1 mM EDTA, 5 mM DTT, and pH 5.5 in a 96-well plate at 27 °C. The peptide inhibitors were pre-incubated with the appropriate proteases in the assay buffer for 10 min to ensure full interactions prior being added to the fluorogenic substrates. The fluorescence of the reaction was monitored as a function of time at 460 nm with an excitation wavelength of 380 nm by a Tecan M1000 plate reader. Each assay was done in triplicates to ensure reproducibility. Details of the analysis for these assays are outlined in the ESI.†

### Cathepsin L activity assay with HepG2 whole cell lysate

Cts L activity in human hepatocellular carcinoma HepG2 whole cell lysate (200 μg at 2.5 mg mL^−1^; ab166833; Abcam, Cambridge, MA) was evaluated using a fluorometric Cathepsin L Activity Assay Kit (ab65306; Abcam, Cambridge, MA, USA) following the manufacturer's protocols. Briefly, in each well of a 96-well plate, 50 μL of the HepG2 cell lysate diluted in CL buffer (to a final concentration of 0.05 mg mL^−1^ HepG2) was incubated with 50 μL of CL buffer without (Control) or with the presence of different peptide inhibitor R^S^_1A_ concentrations (15 μM, 20 μM, 30 μM, 50 μM, 70 μM, 80 μM, 100 μM, and 120 μM). The cell lysate and the peptide inhibitor were incubated at room temperature for 10 min. A total of 2 μL of 10 mM CL substrate Ac-FR-AFC substrate (to a final concentration of 200 μM) was then added to each well, except the Lysate Background Control wells. Different concentrations (56 nM, 560 nM, and 1 μM) of SID 26681509, a known Cts L inhibitor, were used as positive controls. The samples were mixed; the plate was sealed to avoid evaporation and incubated at 37 °C for 1 h. The fluorescence of each sample was measured at 505 nm with an excitation wavelength of 400 nm on the Tecan plate reader. More details of the assay and data fitting are included in the ESI.† Stability assays of the peptides in HepG2 whole cell lysate are also detailed in the ESI.†

### Computational modeling

In order to simulate the protease/peptide complexes from this study, the structure of the papain protease (PDB ID: 1BP4) which contains a peptide-like covalent inhibitor^68^ was used as a template in order to provide a reasonable starting structure for docking. Manual docking was performed by replacing the native covalent inhibitor with the WHLFRAAAW peptide which was prepared using PyRosetta.^69^ The cathepsin proteases of interest, Cts B (PDB ID 1GMY),^70^ Cts K (PDB ID 1BGO),^71^ Cts L (PDB ID 3HHA),^72^ Cts S (PDB ID 1MS6),^73^ and Cts V (PDB ID 1FH0),^74^ were aligned to the manually docked papain complex using PyMOL. The cathepsin protease WHLFRAAAW starting complexes were formally docked by performing the FlexPepDock protocol in Rosetta in order to optimize the binding interaction between the proteases and peptides of interest.^75^ The tryptophan residues in WHLFRAAAW were mutated to 7-methoxycoumarinyl alanine (μ) residues using the MutateResidue tool in PyRosetta toolbox with a params and rotamer library generated previously.^23^ Next, a constrained FastRelax was performed in PyRosetta in order to accommodate the newly mutated 7-methoxycoumarinyl alanine residues. A flat harmonic constraint was used to maintain proximity of the scissile bond to the active site cysteine residue. Thioamides were introduced into the relaxed complexes through patches written previously.^23,76^ The thioamide containing peptides were then simulated with five independent local relax trajectories without any constraints.

### Machine learning

Unsupervised machine learning was performed by clustering energy features from PyRosetta modeling with scikit-learn.^77^ Specifically, score differences (termed deltas) between the residue total energies (energy in thioamide peptide complex minus energy in all-amide peptide complex) of the three residues of the protease catalytic triad as well as the P1 and P1′ residues of the peptide were computed from all of our FlexPepDock models. These five energy score deltas were reduced into three dimensions with Principal Component Analysis.^77^ The three principal component axes were then clustered with the KMeans algorithm utilizing four clusters which was derived by maximizing the Silhouette heuristic.^78^ The three-dimensional data were plotted and visualized with matplotlib.^79^

## Data availability

Kinetic and inhibition data with associated fitting as well as structural models of the peptides in complex with proteases are available as ESI. A key file is provided with descriptions of each data file.

## Author contributions

H. A. T. P. and E. J. P. designed the experiments. H. A. T. P. performed all of the experiments with some guidance and preliminary inhibition data from T. M. B. and C. L. S. G. G. performed computational modeling and machine learning analysis. H. A. T. P. and E. J. P. performed the experimental data analysis and structural model analysis. H. A. T. P. and E. J. P. wrote the manuscript with input from all other authors.

## Conflicts of interest

There are no conflicts to declare.

## Supplementary Material

SC-012-D1SC00785H-s001

SC-012-D1SC00785H-s002

SC-012-D1SC00785H-s003

SC-012-D1SC00785H-s004

SC-012-D1SC00785H-s005

SC-012-D1SC00785H-s006

SC-012-D1SC00785H-s007

SC-012-D1SC00785H-s008

SC-012-D1SC00785H-s009

SC-012-D1SC00785H-s010

SC-012-D1SC00785H-s011

SC-012-D1SC00785H-s012

SC-012-D1SC00785H-s013

SC-012-D1SC00785H-s014

SC-012-D1SC00785H-s015

SC-012-D1SC00785H-s016

SC-012-D1SC00785H-s017

SC-012-D1SC00785H-s018

SC-012-D1SC00785H-s019

SC-012-D1SC00785H-s020

SC-012-D1SC00785H-s021

SC-012-D1SC00785H-s022

SC-012-D1SC00785H-s023

SC-012-D1SC00785H-s024

SC-012-D1SC00785H-s025

SC-012-D1SC00785H-s026

SC-012-D1SC00785H-s027

SC-012-D1SC00785H-s028

SC-012-D1SC00785H-s029

SC-012-D1SC00785H-s030

SC-012-D1SC00785H-s031

SC-012-D1SC00785H-s032

SC-012-D1SC00785H-s033

SC-012-D1SC00785H-s034
