# Supplementary material for: Rational design of thioamide peptides as selective inhibitors of cysteine protease cathepsin L
Source: Chem Sci. 2021 Jul 19;12(32):10825–35. doi: 10.1039/d1sc00785h (PMC8901119; doi:10.1039/d1sc00785h)
Supplement: SC-012-D1SC00785H-s034 [file SC-012-D1SC00785H-s034.pdf]

Electronic Supplementary Material (ESI) for Chemical Science.
This journal is © The Royal Society of Chemistry 2021

Rational Design of Thioamide Peptides as Selective Inhibitors of Cysteine Protease Cathepsin L

Hoang Anh T. Phan,[a] Sam G. Giannakoulias,[a] Taylor M. Barrett,[a] Chunxiao Liu,[a,b] and E. James Petersson[a*]

[a]*Department of Chemistry, University of Pennsylvania, Philadelphia, Pennsylvania 19104, USA*

[b]*Key Laboratory for Northern Urban Agriculture of Ministry of Agriculture and Rural Affairs, Beijing University of Agriculture, Beijing 102206, P. R. China*

[*]Email: ejpetersson@sas.upenn.edu

General Information **S2**
Peptide Inhibitor Design **S3**
Peptide Synthesis, Purification, and Characterization **S4-S7**
Steady-State Protease Activity Analysis **S8-S26**
Cathepsin L Inhibition Assays **S27-S36**
Cathepsin V Inhibition Assays **S37-S38**
Cathepsin K Inhibition Assays **S39-S40**
Cathepsin S Inhibition Assays **S41-S42**
Cathepsin B Inhibition Assays **S43-S44**
Inhibition Assays Analysis **S45-S47**
Cathepsin L Activity Assay with HepG2 Whole Cell Lysate **S48**
Stability of Peptide Inhibitors in HepG2 Whole Cell Lysate **S49-S50**
Computational Modeling **S51-S73**
Unsupervised Machine Learning **S74**
Cell Culture & Cathepsin L Inhibition Assays in MDA-MB-231 Cells **S75-S77**
References **S78-S79**

General Information. Fluorenylmethoxycarbonyl-β-(7-methoxycoumarin-4-yl)-Ala-OH and benzoyl (Z), 7-aminomethylcoumarin (AMC) cathepsin substrates Z-Phe-Arg-AMC (Z-FR-AMC), Z-Leu-Arg-AMC (Z-LR-AMC), and Z-Arg-Arg-AMC (Z-RR-AMC) were purchased from Bachem (Torrance, CA, USA). All other fluorenylmethoxycarbonyl (Fmoc) protected amino acids were purchased from Novabiochem (currently EMD Millipore, MilliporeSigma; Burlington, MA, USA). (7-Azabenzotriazol-1-yloxy)tripyrrolidino-phosphonium hexafluorophosphate (PyAOP) was purchased from Chem-Impex (Wood Dale, IL, USA). Piperidine, *N*,*N*-diisopropylethylamine (DIPEA), cathepsin B (Cts B, ≥1,500 units/mg protein, solution; from human liver), cathepsin K (Cts K, 0.5 mg/mL, solution; recombinant, expressed in *E. coli*, ≥90% pure by SDS-PAGE), cathepsin L (Cts L, ≥ 0.5 units/mg protein, solution; from human liver), cathepsin S (Cts S, 0.2 mg/mL or 2 mg/mL, solution; recombinant, expressed in FreeStyle™ 293-F cells, ≥90% pure by SDS-PAGE), and cathepsin V (Cts V, 0.38 mg/mL, solution; recombinant, expressed in FreeStyle™ 293-F cells, ≥90% pure by SDS-PAGE) were purchased from Sigma-Aldrich (St. Louis, MO, USA). All other reagents were purchased from Fisher Scientific (Pittsburgh, PA, USA) unless specified otherwise. Milli-Q filtered (18 MΩ) water was used for all solutions (EMD Millipore). Peptides were purified with an Agilent 1260 Infinity II Preparative HPLC system and analyzed with an Agilent 1260 Infinity II Analytical HPLC system (Santa Clara, CA, USA). Peptide mass spectrometry was collected with a Bruker Ultraflex III matrix-assisted laser desorption ionization mass spectrometer (MALDI MS) (Billerica, MA, USA). Time-course UV-Vis absorbance and fluorescence data were obtained with a Tecan Infinite® M1000 PRO plate reader (Männedorf, Switzerland).

Peptide Design

The primed positions of our peptides were kept generic and consistent with our previous studies by having alanine at the P1', P2', and P3' positions. For the non-primed positions, sequence design was guided by a comprehensive substrate profiling study by Choe *et al.*[1]

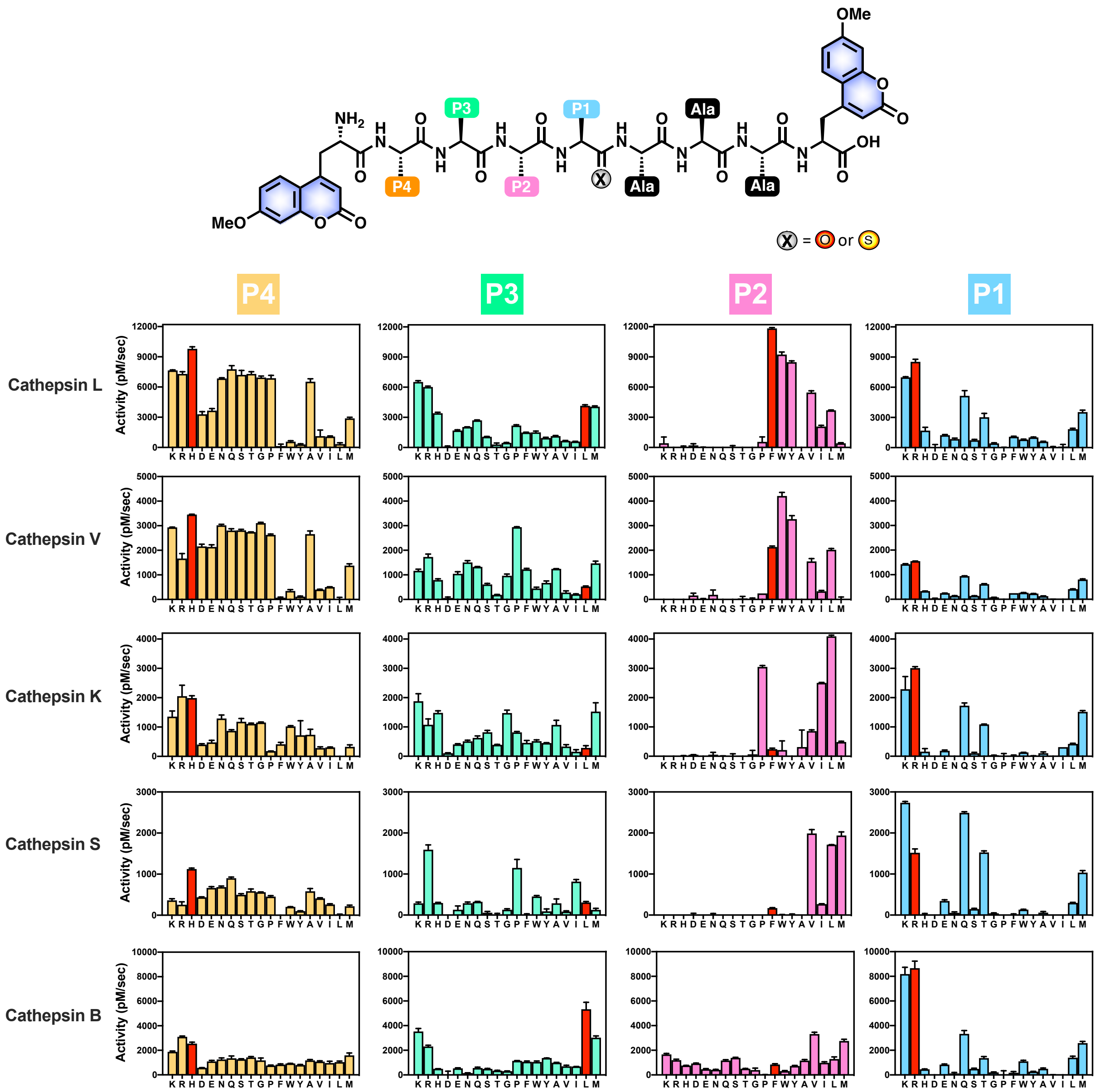

Figure S1. Cathepsin L (Cts L) Inhibitor Design. *Top:* Our peptide inhibitor design. The peptides contain 7-methoxycoumarin-4-yl-alanine (MCM; μ) residues at both termini, and either an amide (X=O) or a thioamide (X=S) residue at the P1 position. *Bottom:* Substrate specificity of human cathepsins by scanning with Acetyl-P4-P3-P2-P1-aminomethylcoumarin peptides from Choe *et al.*[1] The red bars denote the residues we chose for the non-primed positions (P4, P3, P2, P1) for our desired peptide inhibitor of Cts L.

Peptide Synthesis, Purification, and Characterization.

Synthesis of Thioamino acid Precursors. N_α-Fmoc-N_e-Boc-L-thiolysine-nitrobenzo triazolide and N_α-Fmoc-N_ω-Pbf-L-thioarginine-nitrobenzotriazolide were synthesized and characterized as previously published procedures by our laboratory.[2, 3]

Peptide Synthesis. Each peptide was manually synthesized on a 25 μmol scale on 2-chlorotrityl resin based on our established protocols.[4] For a typical synthesis, 2-chlorotrityl resin was added to a dry reaction vessel (RV) and initially swelled in 5 mL dimethylformamide (DMF) for 30 min with magnetic stirring. Between each reaction, the resin was washed extensively with adequate DMF. For the first amino acid coupling, 5 equiv. of amino acid was dissolved in 1 mL of DMF and added to the RV, following an addition of 10 equiv. of DIPEA. The reaction ran for 45 min under stirring. If the first amino acid was Fmoc-β-(7-methoxycoumarin-4-yl)-Ala-OH, instead, 2 equiv. of the amino acid was dissolved in 1 mL of DMF with 4 equiv. of DIPEA and stirred for 30 min; this reaction was repeated to ensure efficient coupling. After washing, the resin was incubated with 2 mL of 20% piperidine solution in DMF for 20 mins under stirring for deprotection. The same deprotection procedure was followed for all the subsequent standard amino acids. For a typical 45-minute coupling reaction, 5 equiv. of the standard amino acid and 5 equiv. of PyAOP was dissolved in 1 mL DMF, and added to the RV, with an addition of 10 equiv. of DIPEA. For Fmoc-β-(7-methoxycoumarin-4-yl)-Ala-OH, 2 equiv. of the amino acid and 2 equiv. of PyAOP were dissolved in 1 mL DMF, then added to the RV with an addition of 4 equiv. of DIPEA and stirred for 30 min; a second coupling reaction was repeated before deprotection. Thioamide residues were coupled and deprotected with slightly modified procedures. Thioamides were coupled through pre-activated precursors, where 3 equiv. of the thioamide precursor was dissolved in 1.5 mL of dry dichloromethane (DCM) with 6 equiv. DIPEA and stirred for 30 min. This

procedure was repeated prior to deprotection to ensure efficient incorporation of the thioaminoacid onto the peptide chains. For the deprotection of thioamides, 2 mL of 2% DBU (1,8-diazabicyclo(5.4.0)undec-7-ene) in DMF was added to the RV and reacted three times for 2 min each, with extensive washing with DMF and DCM between each deprotection step.

Peptide Cleavage and Purification. Upon completion of the synthesis, the resin was dried with DCM under vacuum. Peptides were cleaved from resin by treatment with a 2 mL fresh cleavage cocktail of trifluoroacetic acid (TFA), water, and triisopropylsilane (TIPSH) (95:2.5:2.5 v/v) for 45 mins with stirring. After treatment, the cocktail solution was expelled from the RV with nitrogen and reduced to a volume of less than 1 mL by rotary evaporation. This resulting solution was then treated with over 10 mL of cold ethyl ether in order to precipitate out the peptides. This precipitate was flash frozen with liquid nitrogen and evaporated using lyophilization. The crude peptide was diluted in CH_3CN/H_2O (10:90 v/v) and then purified on a Luna® Omega 5 µm PS C18 100 Å, LC semi-preparative column (Phenomenex; Torrance, CA, USA) by HPLC using the following gradients (**Tables S1 & S2**) at a flow rate of 4 mL/min. MALDI MS was used to confirm peptide identities (**Table S3**). Purified peptides were dried on a lyophilizer (Labconco; Kansas City, MO, USA) or in a vacuum centrifuge (Savant/Thermo Scientific; Rockford, IL, USA). If necessary, peptides were subjected to multiple rounds of purification until 99% purity by analytical HPLC was achieved. Details of the synthesis, purification, and MALDI MS characterization for the µLLK^{S}AAAµ (K$^S_{P1}$) peptide were previously published in Liu *et al*. (2019).[3]

Table S1. Peptide Purification Methods and Retention Time.

Peptide	Gradient	Retention Time
μHLFKAAAμ	1	23 min
μHLFK^{S}AAAμ	1	24 min
μHLFRAAAμ	2	18 min
μHLFR^{S}AAAμ	2	19 min
μHLFRAμ	2	16 min
μHLFR^{S}Aμ	2	18 min
HLFR^{S}A	3	9 min

* Abbreviations: μ: 7-methoxycoumarinylalanine; K^{S}: thiolysine; R^{S}: thioarginine.

Table S2. HPLC Gradients for Peptide Purification.

No.	Time (min)	%B	No.	Time (min)	%B
1	0:00	10	2	0:00	10
	2:00	10		2:00	10
	5:00	20		5:00	20
	10:00	20		25:00	40
	30:00	40		26:00	40
	33:00	40		27:00	100
	35:00	100		30:00	100
	45:00	100		35:00	10
	48:00	10			
3	0:00	10			
	2:00	10			
	5:00	20			
	17:00	30			
	18:00	30			
	19:00	100			
	22:00	100			
	27:00	10			

* Solvent A: 0.1 % TFA in water; Solvent B: 0.1 % TFA in acetonitrile

Table S3. Calculated and Observed Masses of Peptides.

**Peptide**	**[M+H]$^+$**		**[M+Na]$^+$**		**[M+K]$^+$**	
	**Calculated**	**Observed**	**Calculated**	**Observed**	**Calculated**	**Observed**
μHLFKAAAμ	1247.57	1247.74	1269.56	1269.72	1285.53	1285.72
μHLFK^{S}AAAμ	1263.55	1263.48	1285.53	1285.55	1301.51	1301.57
μHLFRAAAμ	1275.58	1275.79	1297.56	1297.80	1313.54	1313.73
μHLFR^{S}AAAμ	1291.56	1291.70	1313.54	1313.71	1329.51	1329.64
μHLFRAμ	1133.51	1133.37	1155.49	1155.36	1171.46	1171.32
μHLFR^{S}Aμ	1149.48	1149.42	1171.46	1171.42	1187.44	1187.38
HLFR^{S}A	659.35	659.45	681.33	681.44	697.30	697.41

* Abbreviations: μ: 7-methoxycoumarin-4-yl-alanine; K^S: thiolysine; R^S: thioarginine.

Steady-State Protease Activity Analysis

Steady State Protease Assays. In a typical trial, a 7.5 μM peptide solution was incubated in the absence or presence of the appropriate concentration of Cts L (30.3 nM), Cts V (20.5 nM), Cts K (42.6 nM), Cts S (21.6 nM), or Cts B (37.6 nM) in a buffer of 100 mM sodium acetate, 100 mM NaCl, 1 mM EDTA, 5 mM DTT, and pH 5.5 at 27 °C; assays with R_{1A} peptide (μHLFRAμ) were supplemented with 2% DMSO. The fluorescence was monitored as a function of time at 390 nm with an excitation wavelength of 325 nm on the Tecan M1000 plate reader. Three independent trials were performed for each assay to ensure reproducibility. These primary data are shown in **Figures S2-S6**. The data from the three trials were averaged and cleavage percentages were calculated by dividing the fluorescence change of each time point by the fluorescence change of complete cleavage of the peptide. Initial rates were determined by fitting a line to the linear portion of the curve using a linear regression function in Prism 8 (GraphPad Software; La Jolla, CA). These rates, along with the standard errors, are reported in **Table S4** (or **Table 1** in the main text). MALDI MS (**Tables S6-S11**) & HPLC analysis (**Figures S7-S12**) of peptide proteolysis by all five proteases were also done to confirm the cleavage sites, indicated by the slashes (*e.g.* μHLFK/AAAμ) as summarized in **Table S5**. A Phenomenex Luna® Omega 5 μm PS C18 100 Å analytical HPLC column was used to analyze all samples using the same gradient of 20-40 % B over 20 minutes (Solvent A: 0.1% TFA in Milli-Q water; Solvent B: 0.1% TFA in acetonitrile).

Table S4. Initial Rates of Peptides Cleavage by Cysteine Cathepsin Proteases.

Peptide	Cts L	Cts V	Cts K	Cts S	Cts B
μHLFKAAAμ	0.181 ± 0.003	0.511 ± 0.025	0.689 ± 0.045	0.110 ± 0.002	1.921 ± 0.206
μHLFK^{S}AAAμ	0.014 ± 0.000	0.002 ± 0.000*	0.002 ± 0.000*	0.000 ± 0.000*	3.643 ± 0.153
μHLFRAAAμ	0.112 ± 0.003	0.363 ± 0.014	0.564 ± 0.024	0.189 ± 0.004	2.747 ± 0.265
μHLFR^{S}AAAμ	0.023 ± 0.000	0.002 ± 0.000*	0.001 ± 0.000*	0.002 ± 0.000*	3.968 ± 0.209
μHLFRAμ	0.125 ± 0.003	0.553 ± 0.021	0.402 ± 0.021	0.053 ± 0.000	1.034 ± 0.074
μHLFR^{S}Aμ	0.000 ± 0.000*	0.005 ± 0.000*	0.000 ± 0.000*	0.000 ± 0.000*	0.003 ± 0.000*

All rates are reported in $\mu M{\cdot}min^{-1}$. Rates and standard errors are calculated by fitting to linear regression function in Prism 8. *MALDI MS and HPLC confirmed no cleavage with these peptides.

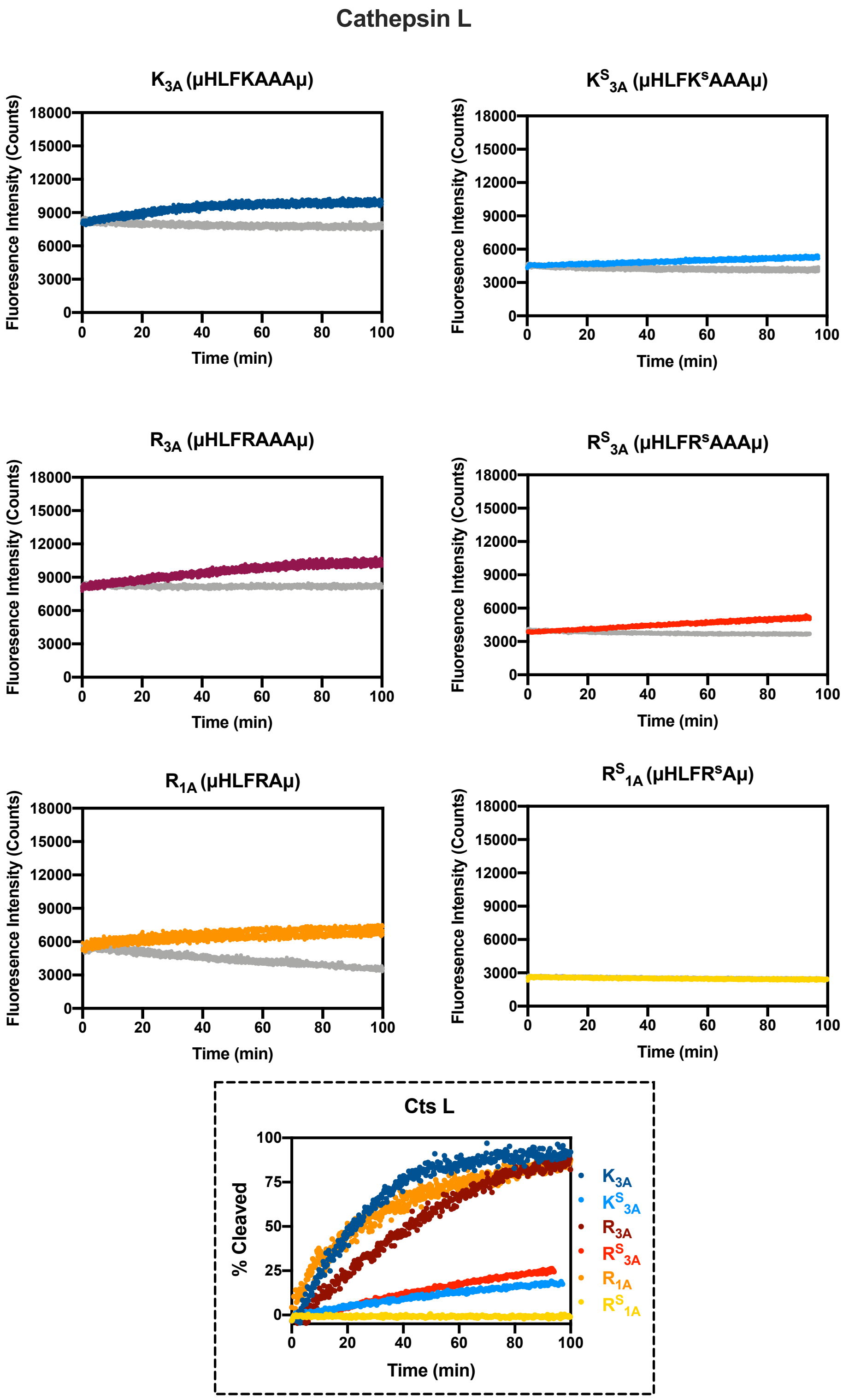

Figure S2. Proteolysis of Cathepsin L (Cts L) Monitored by Fluorescence at 390 nm. Peptides (7.5 µM) were incubated in the presence (colored traces) or absence (gray traces) of 30.3 nM Cts L in 100 mM sodium acetate, 100 mM NaCl, 1 mM EDTA, 5 mM DTT, pH 5.5 at 27 ºC. Fluorescence traces of three trials are overlayed. *Inset:* Summary of the normalized cleavage data for the peptides with Cts L.

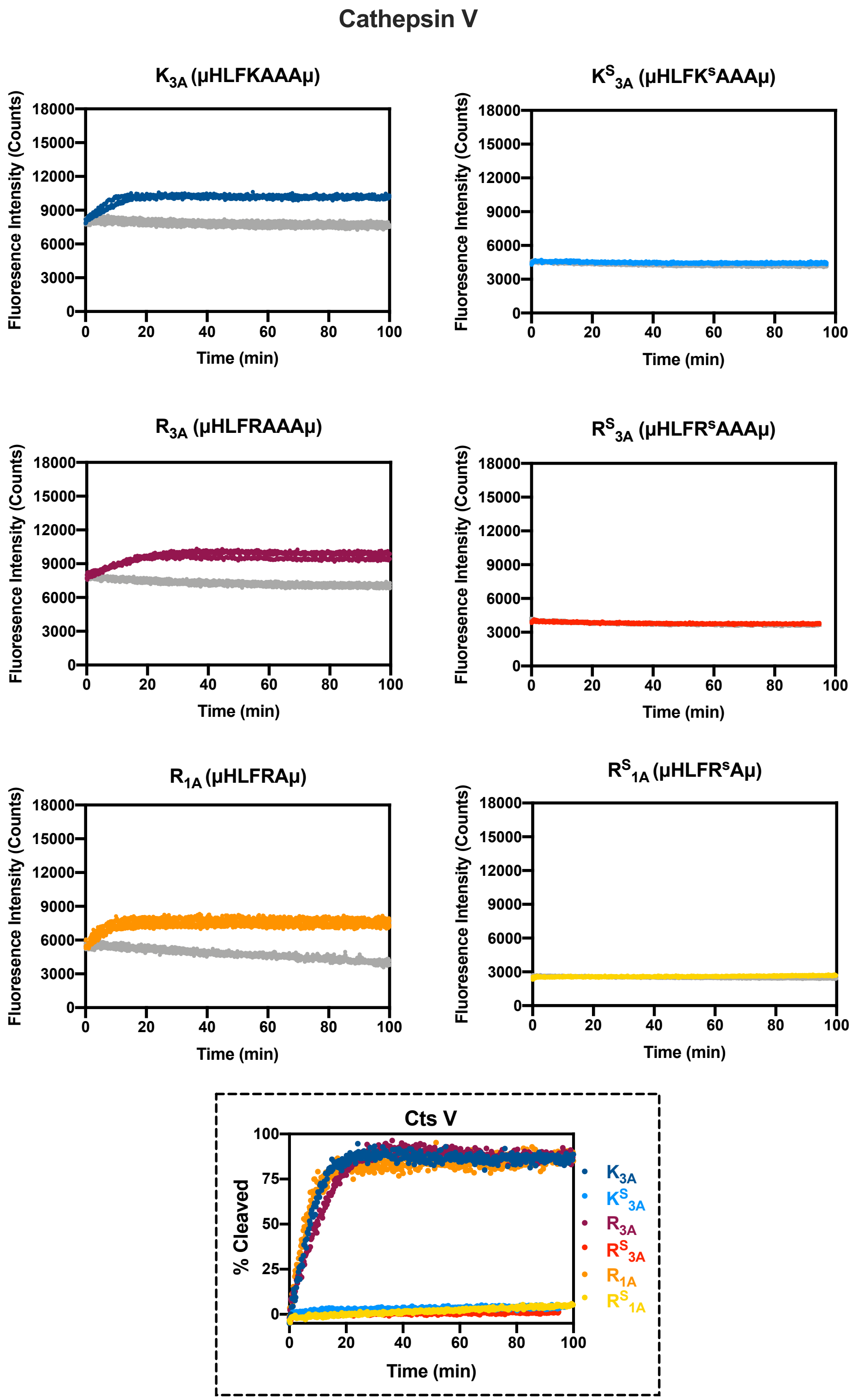

Figure S3. Proteolysis of Cathepsin V (Cts V) Monitored by Fluorescence at 390 nm. Peptides (7.5 µM) were incubated in the presence (colored traces) or absence (gray traces) of 20.5 nM Cts V in 100 mM sodium acetate, 100 mM NaCl, 1 mM EDTA, 5 mM DTT, pH 5.5 at 27 ºC. Fluorescence traces of three trials are overlayed. *Inset:* Summary of the normalized cleavage data for the peptides with Cts V.

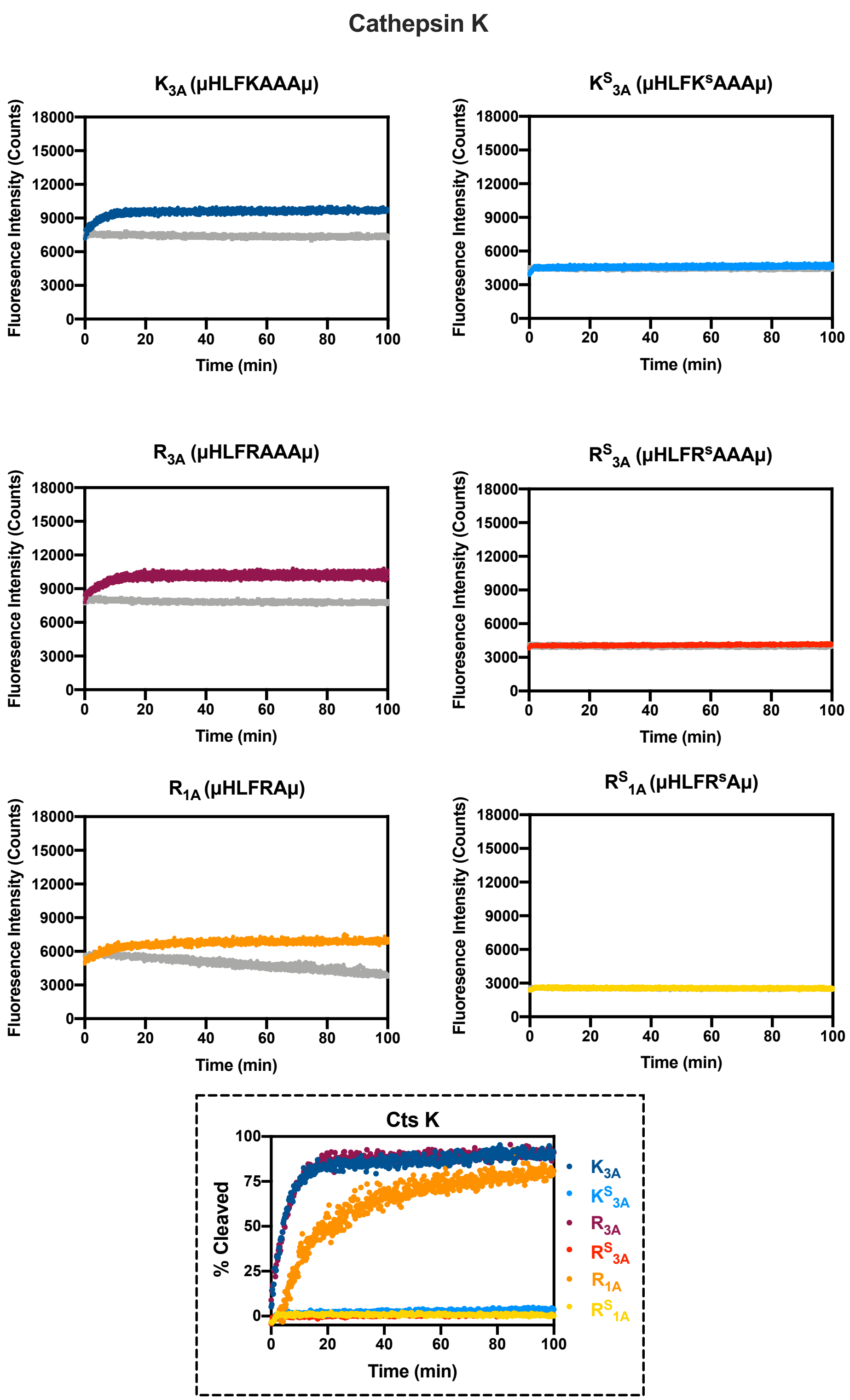

Figure S4. Proteolysis of Cathepsin K (Cts K) Monitored by Fluorescence at 390 nm. Peptides (7.5 µM) were incubated in the presence (colored traces) or absence (gray traces) of 42.6 nM Cts K in 100 mM sodium acetate, 100 mM NaCl, 1 mM EDTA, 5 mM DTT, pH 5.5 at 27 ºC. Fluorescence traces of three trials are overlayed. *Inset:* Summary of the normalized cleavage data for the peptides with Cts K.

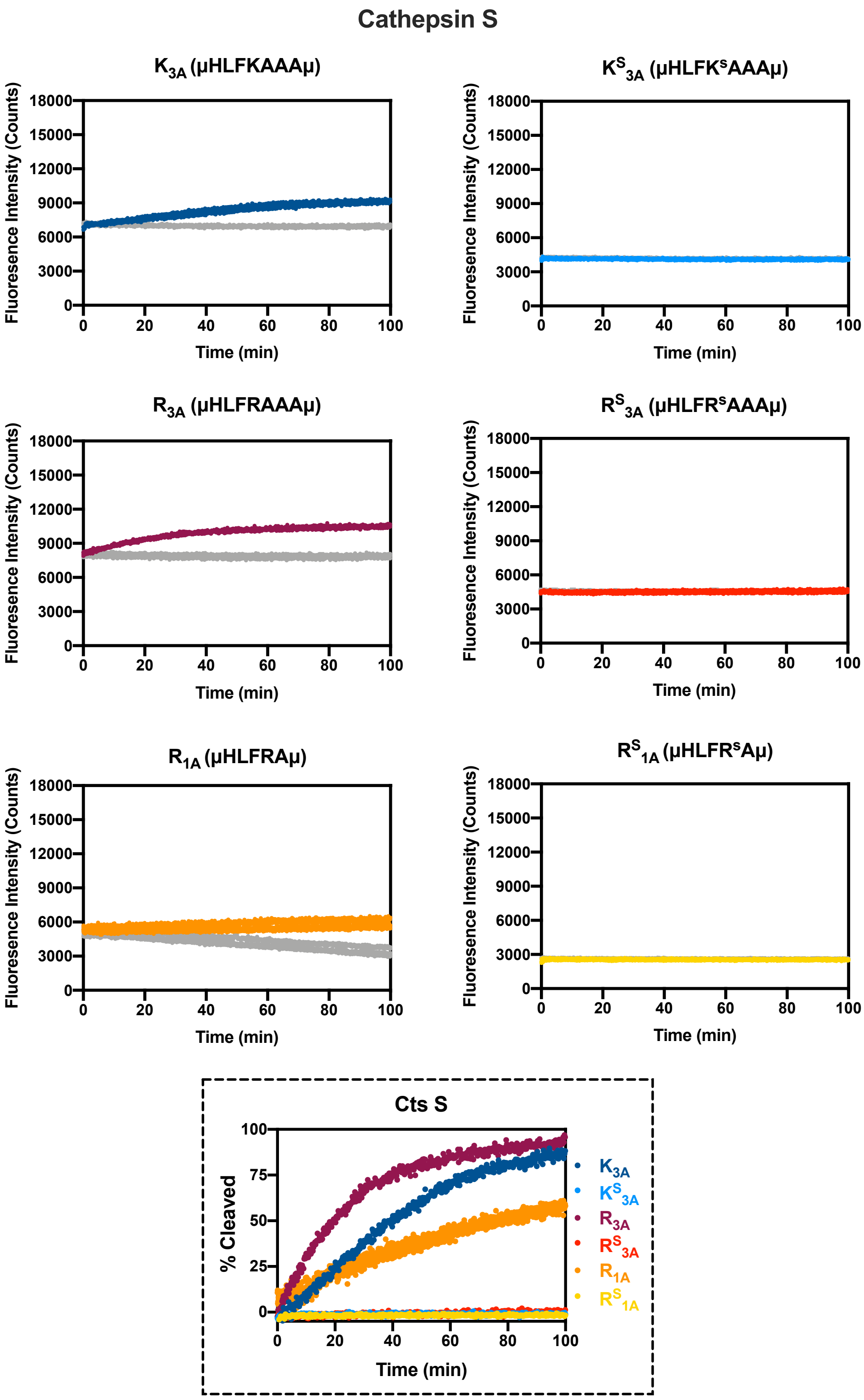

Figure S5. Proteolysis of Cathepsin S (Cts S) Monitored by Fluorescence at 390 nm. Peptides (7.5 μM) were incubated in the presence (colored traces) or absence (gray traces) of 21.6 nM Cts S in 100 mM sodium acetate, 100 mM NaCl, 1 mM EDTA, 5 mM DTT, pH 5.5 at 27 ºC. Fluorescence traces of three trials are overlayed. *Inset:* Summary of the normalized cleavage data for the peptides with Cts S.

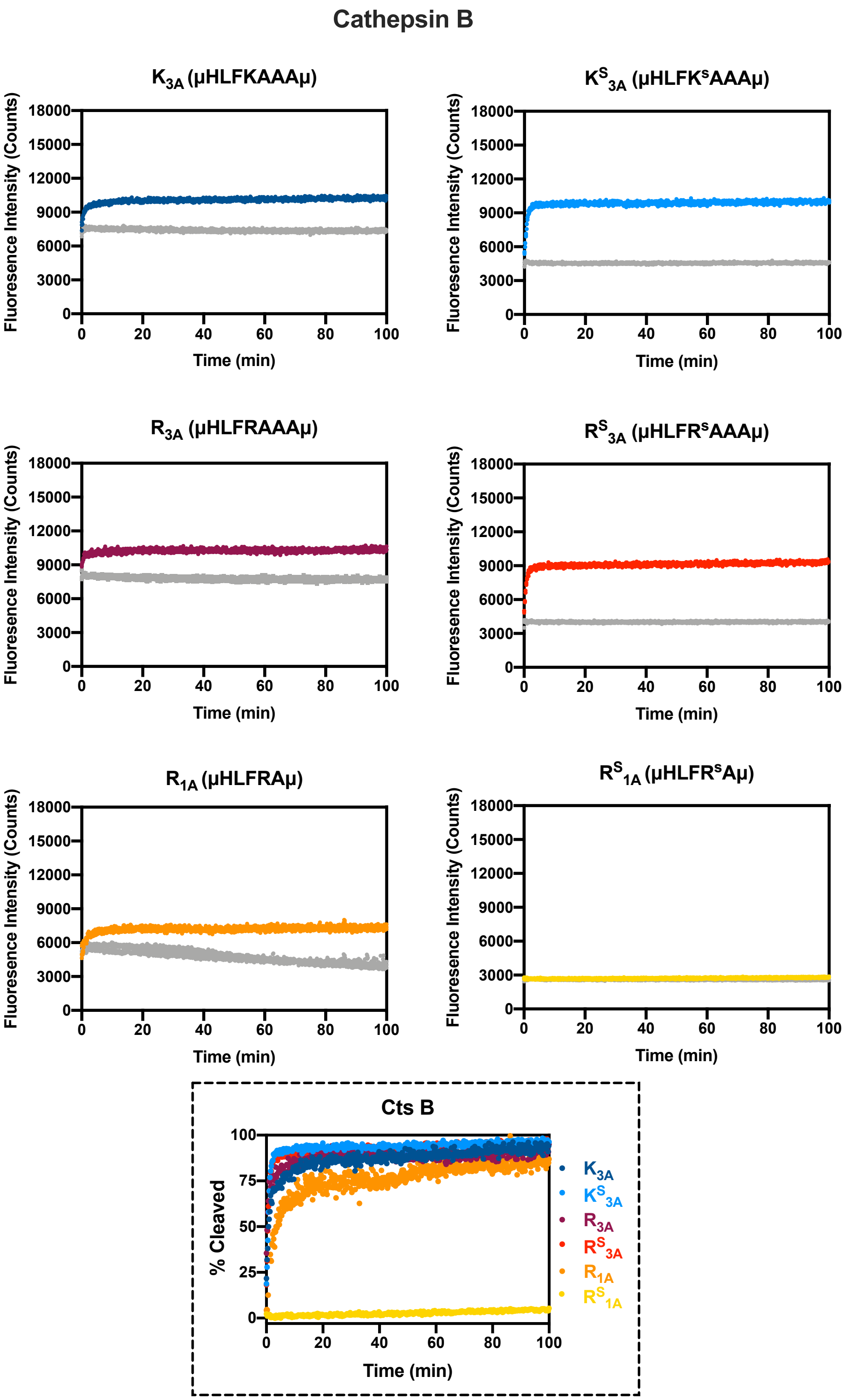

Figure S6. Proteolysis of Cathepsin B (Cts B) Monitored by Fluorescence at 390 nm. Peptides (7.5 μM) were incubated in the presence (colored traces) or absence (gray traces) of 37.6 nM Cts B in 100 mM sodium acetate, 100 mM NaCl, 1 mM EDTA, 5 mM DTT, pH 5.5 at 27 ºC. Fluorescence traces of three trials are overlayed. *Inset:* Summary of the normalized cleavage data for the peptides with Cts B.

Table S5. Summary of Cleavage Sites of Peptides with Different Cathepsins from Steady State Protease Assays. The dashes indicate cleavage sites by the corresponding proteases.

Peptides	Cts L	Cts V	Cts K	Cts S	Cts B
K_{3A}	μHLF/K/A/AAμ	μHLF/K/AAAμ	μHLF/K/AAAμ	μHLFK/AAAμ	μHLFK/AAAμ
K^S_{3A}	μHLFK^{S}AA/Aμ	No cleavage	No cleavage	No cleavage	μHLFK^{S}AA/Aμ
R_{3A}	μHLF/R/AAAμ	μHLF/R/AAAμ	μHLF/R/AAAμ	μHLFR/AAAμ	μHLFR/AAAμ
R^S_{3A}	μHLFR^{S}AA/Aμ	No cleavage	No cleavage	No cleavage	μHLFR^{S}AA/Aμ
R_{1A}	μHLF/R/Aμ	μHLF/R/Aμ	μHLF/R/Aμ	μHLF/R/Aμ	μHLFR/Aμ
R^S_{1A}	No cleavage	No cleavage	No cleavage	No cleavage	No cleavage

Table S6. Masses Identified from Protease Assays with K_{3A} Peptide (μHLFKAAAμ).

Protease	Fragment	$[M+H]^+$		$[M+Na]^+$		$[M+2Na-H]^+$	
		**Calculated**	**Observed**	**Calculated**	**Observed**	**Calculated**	**Observed**
**No protease** ***(Negative Control)***	μHLFKAAAμ *(Intact peptide)*	1247.57	1247.71	1269.56	1269.69	1291.54	1291.09
**Cts L**	μHLF	661.30	661.50	683.28	683.37	705.27	705.40
	KAAAμ	605.29	605.40	627.28	627.37	649.26	649.37
	μHLFK	789.39	789.55	811.38	811.53	833.36	833.52
	AAAμ	477.20	-	499.18	-	521.16	521.27
	μHLFKA	860.43	861.19	882.41	-	904.39	-
	AAμ	406.16	-	428.14	-	450.12	450.42
**Cts V**	μHLF	661.30	661.48	683.28	683.45	705.27	705.43
	KAAAμ	605.29	605.39	627.28	627.37	649.26	649.33
	μHLFK	789.39	789.60	811.38	811.58	833.36	833.55
	AAAμ	477.20	-	499.18	-	521.16	522.74
**Cts K**	μHLF	661.30	661.32	683.28	683.30	705.27	705.28
	KAAAμ	605.29	605.30	627.28	627.28	649.26	649.26
	μHLFK	789.39	789.44	811.38	811.42	833.36	833.41
	AAAμ	477.20	-	499.18	-	521.16	521.17
**Cts S**	μHLFK	789.39	789.53	811.38	811.51	833.36	833.44
	AAAμ	477.20	-	499.18	-	521.16	522.72
**Cts B**	μHLFK	789.39	789.52	811.38	811.14	833.36	833.52
	AAAμ	477.20	-	499.18	-	521.16	522.71

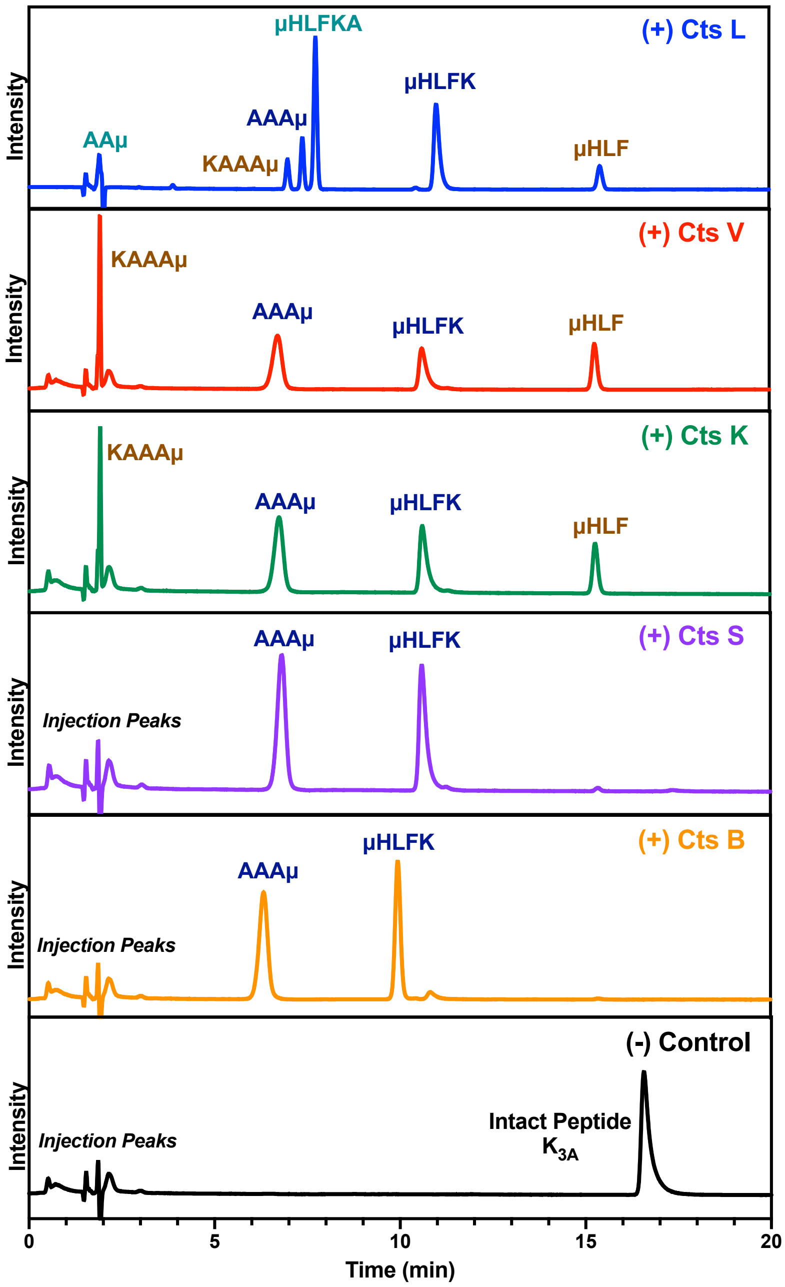

Figure S7. HPLC Analysis (325 nm) of K_{3A} Peptide (μHLFKAAAμ) Cleaved by Different Cathepsins. Reaction mixtures from fluorescence assays were analyzed by HPLC at 325 nm after completion of the reactions.

Table S7. Masses Identified from Protease Assays with K^{S}_{3A} Peptide (µHLFK^{S}AAAµ).

**Protease**	**Fragment**	**[M+H]$^+$**		**[M+Na]$^+$**		**[M+2Na-H]$^+$**	
		**Calculated**	**Observed**	**Calculated**	**Observed**	**Calculated**	**Observed**
**No protease** ***(Negative Control)***	µHLFK^{S}AAAµ *(Intact peptide)*	1263.55	1263.38	1285.53	1285.36	1307.51	1307.33
**Cts L**	µHLFK^{S}AAAµ	1263.55	1263.49	1285.53	1285.43	1307.51	1307.43
	µHLFK^{S}AA	947.44	947.36	969.43	969.33	991.41	991.31
	Aµ	335.12	335.15	357.11	357.26	379.09	379.14
**Cts V**	µHLFK^{S}AAAµ	1263.55	1263.44	1285.53	1285.42	1307.51	1307.39
**Cts K**	µHLFK^{S}AAAµ	1263.55	1263.42	1285.53	1285.39	1307.51	1307.39
**Cts S**	µHLFK^{S}AAAµ	1263.55	1263.41	1285.53	1285.40	1307.51	1307.39
**Cts B**	µHLFK^{S}AA	947.44	947.32	969.43	969.31	991.41	991.29
	Aµ	335.12	335.33	357.11	-	379.09	-

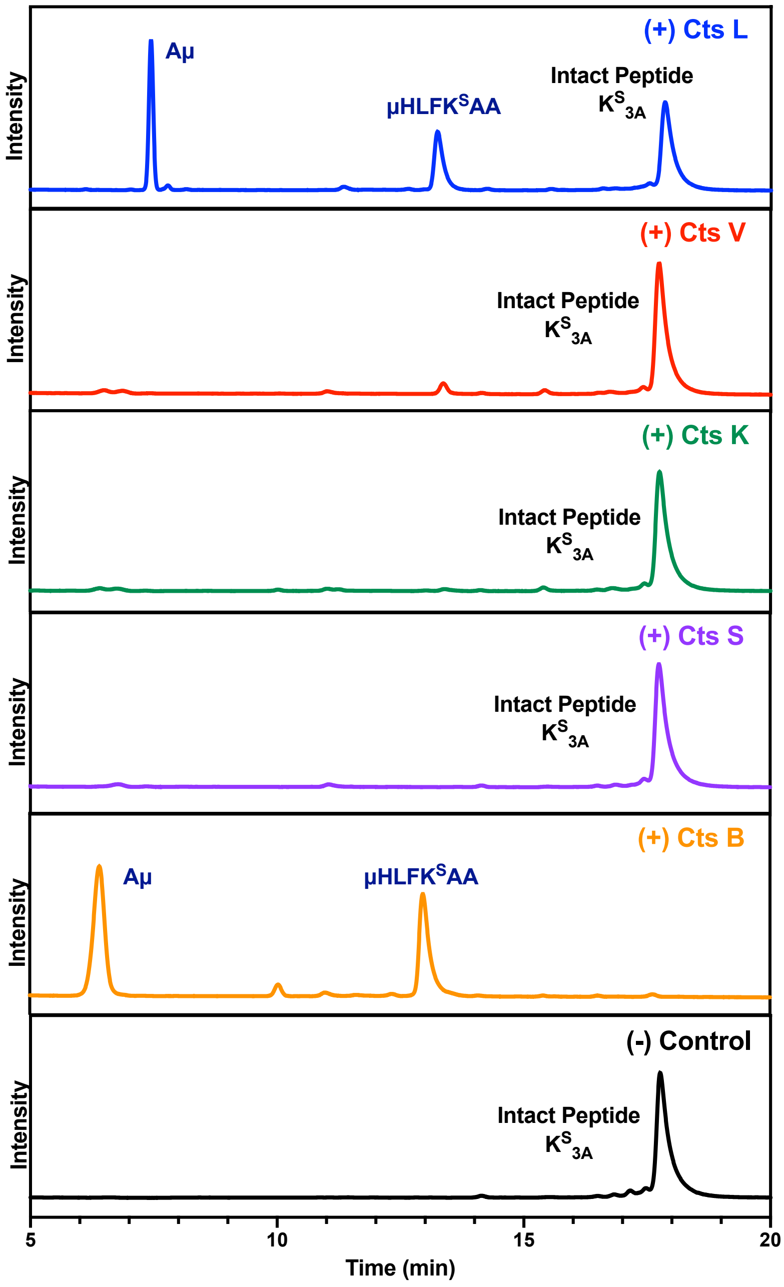

Figure S8. HPLC Analysis (325 nm) of K^{S}_{3A} Peptide (μHLFK^{S}AAAμ) Cleaved by Different Cathepsins. Reaction mixtures from fluorescence assays were analyzed by HPLC after completion of the reactions.

Table S8. Masses Identified from Protease Assays with R_{3A} Peptide (μHLFRAAAμ).

**Protease**	**Fragment**	**[M+H]⁺**		**[M+Na]⁺**		**[M+2Na-H]⁺**	
		**Calculated**	**Observed**	**Calculated**	**Observed**	**Calculated**	**Observed**
**No protease** ***(Negative Control)***	μHLFRAAAμ *(Intact peptide)*	1275.58	1275.67	1297.56	1297.67	1319.54	1319.64
**Cts L**	μHLF	661.30	661.57	683.28	683.56	705.26	705.54
	RAAAμ	633.30	633.35	655.28	655.30	677.26	677.31
	μHLFR	817.40	817.46	839.38	839.45	861.36	861.43
	AAAμ	477.20	-	499.18	-	521.16	522.67
**Cts V**	μHLF	661.30	661.43	683.28	683.40	705.26	705.38
	RAAAμ	633.30	633.41	655.28	655.40	677.26	677.38
	μHLFR	817.40	817.52	839.38	839.52	861.36	861.50
	AAAμ	477.20	-	499.18	-	521.16	522.77
**Cts K**	μHLF	661.30	661.55	683.28	683.54	705.26	705.78
	RAAAμ	633.30	633.39	655.28	655.38	677.26	677.37
	μHLFR	817.40	817.49	839.38	839.49	861.36	861.47
	AAAμ	477.20	-	499.18	-	521.16	522.75
**Cts S**	μHLFR	817.40	817.35	839.38	839.46	861.36	861.44
	AAAμ	477.20	-	499.18	-	521.16	522.28
**Cts B**	μHLFR	817.40	817.50	839.38	839.49	861.36	861.48
	AAAμ	477.20	-	499.18	-	521.16	522.76

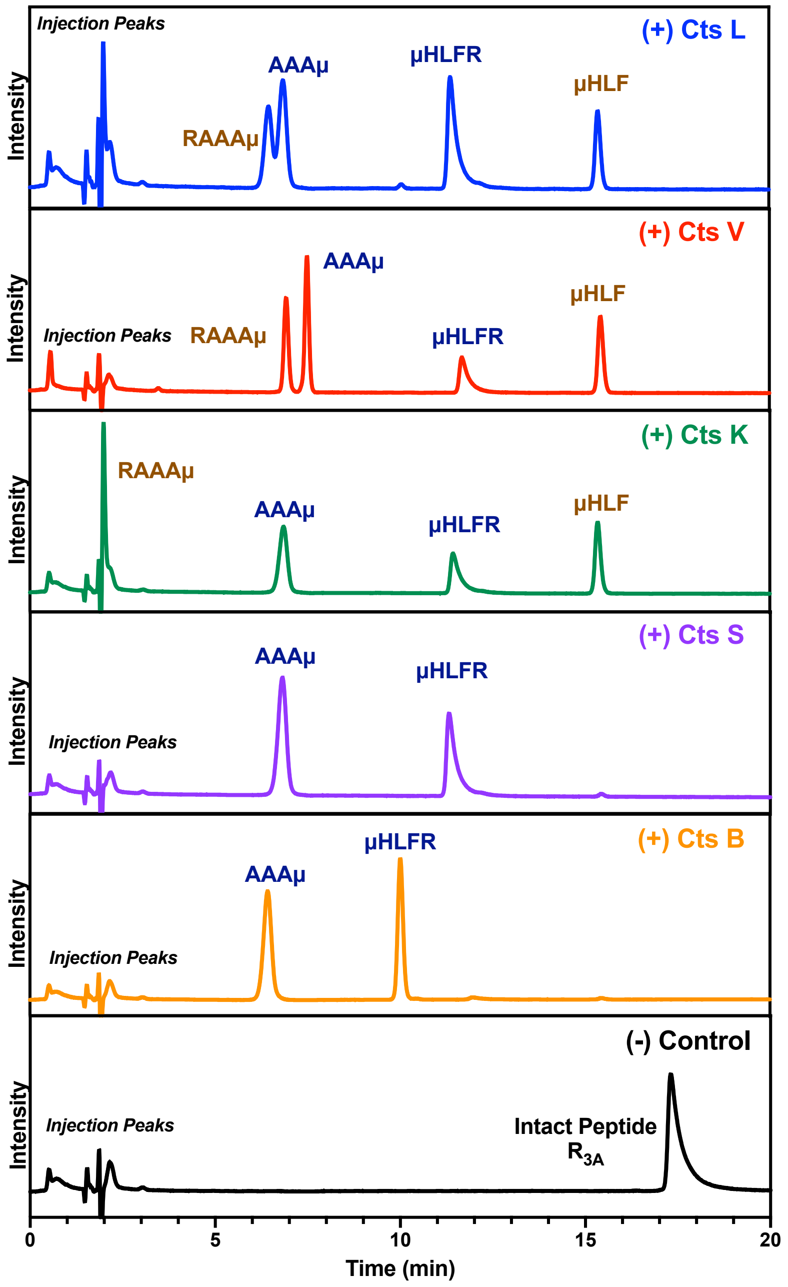

Figure S9. HPLC Analysis (325 nm) of R_{3A} Peptide (µHLFRAAAµ) Cleaved by Different Cathepsins. Reaction mixtures from fluorescence assays were analyzed by HPLC after completion of the reactions.

Table S9. Masses Identified from Protease Assays with R^{S}_{3A} Peptide (μHLFR^{S}AAAμ).

**Protease**	**Fragment**	**[M+H]$^{+}$**		**[M+Na]$^{+}$**		**[M+2Na-H]$^{+}$**	
		**Calculated**	**Observed**	**Calculated**	**Observed**	**Calculated**	**Observed**
**No protease** ***(Negative Control)***	μHLFR^{S}AAAμ *(Intact peptide)*	1291.56	1291.36	1313.54	1313.36	1335.52	1335.37
**Cts L**	μHLFR^{S}AAAμ	1291.56	1291.28	1313.54	1313.23	1335.52	1335.90
	μHLFR^{S}AA	975.45	975.81	997.43	997.78	1019.41	1019.83
	Aμ	335.12	335.08	357.11	-	379.09	379.10
**Cts V**	μHLFR^{S}AAAμ	1291.56	1291.30	1313.54	1313.27	1335.52	1335.26
**Cts K**	μHLFR^{S}AAAμ	1291.56	1291.32	1313.54	1313.34	1335.52	1335.26
**Cts S**	μHLFR^{S}AAAμ	1291.56	1291.32	1313.54	1313.30	1335.52	1335.22
**Cts B**	μHLFR^{S}AA	975.45	975.27	997.43	997.25	1019.41	1019.24
	Aμ	335.12	335.20	357.11	357.23	379.09	379.21

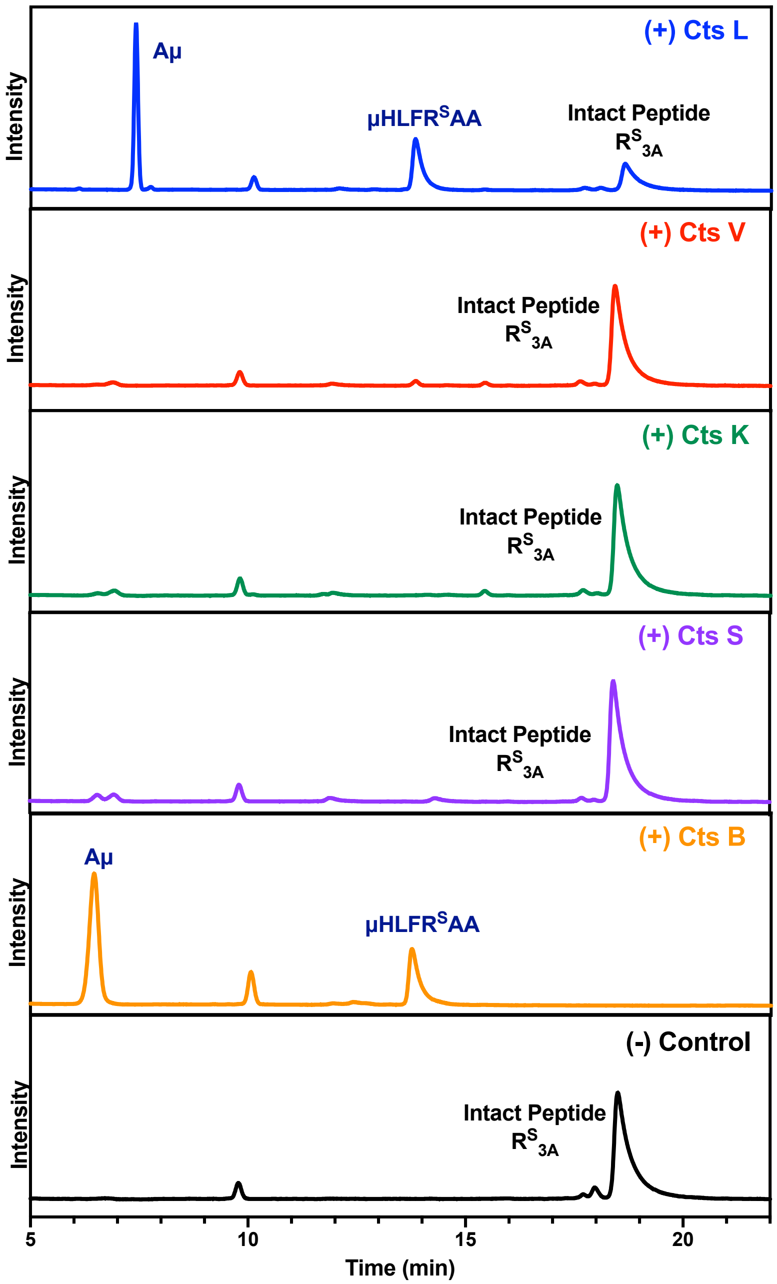

Figure S10. HPLC Analysis (325 nm) of R^{S}_{3A} Peptide (μHLFR^{S}AAAμ) Cleaved by Different Cathepsins. Reaction mixtures from fluorescence assays were analyzed by HPLC after completion of the reactions.

Table S10. Masses Identified from Protease Assays with R_{1A} Peptide (μHLFRAμ).

Protease	Fragment	[M+H]$^+$		[M+Na]$^+$		[M+2Na-H]$^+$	
		**Calculated**	**Observed**	**Calculated**	**Observed**	**Calculated**	**Observed**
**No protease** *(Negative Control)*	μHLFRAμ *(Intact peptide)*	1133.51	1133.57	1155.49	1155.57	1177.47	1177.56
**Cts L**	μHLF	661.30	661.34	683.28	683.37	705.26	705.31
	RAμ	491.23	491.52	513.21	513.56	535.19	535.63
	μHLFR	817.40	817.42	839.38	839.47	861.36	861.44
	Aμ	335.12	335.27	357.11	357.30	379.09	379.28
**Cts V**	μHLF	661.30	661.38	683.28	683.38	705.26	705.34
	RAμ	491.23	491.49	513.21	513.58	535.19	533.60
	μHLFR	817.40	817.45	839.38	839.43	861.36	861.38
	Aμ	335.12	335.26	357.11	357.29	379.09	379.27
**Cts K**	μHLF	661.30	661.40	683.28	683.37	705.26	705.36
	RAμ	491.23	491.52	513.21	513.61	535.19	533.64
	μHLFR	817.40	817.53	839.38	839.56	861.36	861.53
	Aμ	335.12	335.29	357.11	361.31	379.09	379.33
**Cts S**	μHLF	661.30	661.36	683.28	683.34	705.26	705.32
	RAμ	491.23	491.52	513.21	-	535.19	535.30
	μHLFR	817.40	817.45	839.38	839.46	861.36	861.43
	Aμ	335.12	335.27	357.11	357.31	379.09	379.29
**Cts B**	μHLFR	817.40	817.45	839.38	839.45	861.36	861.44
	Aμ	335.12	335.27	357.11	361.34	379.09	379.31

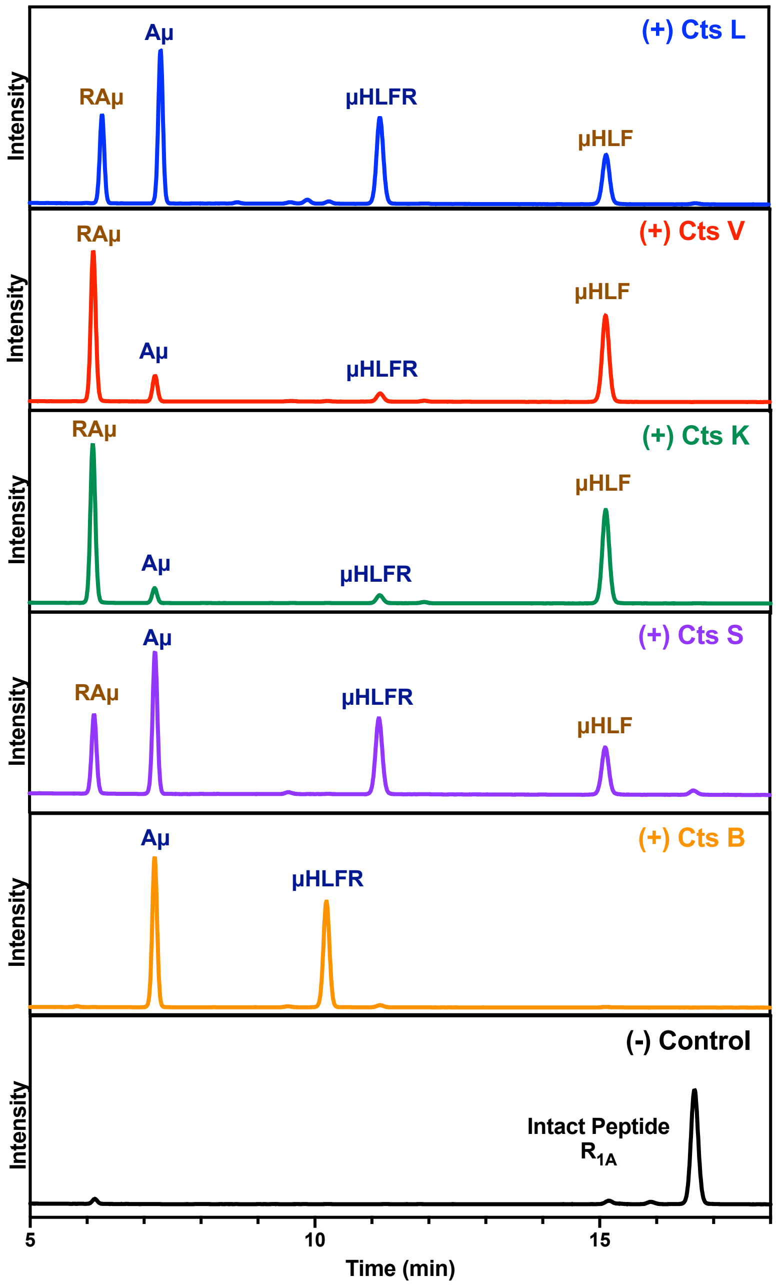

Figure S11. HPLC Analysis (325 nm) of R_{1A} Peptide (μHLFRAμ) Cleaved by Different Cathepsins. Reaction mixtures from fluorescence assays were analyzed by HPLC after completion of the reactions.

Table S11. Masses Identified from Protease Assays with $R^{S}{}_{1A}$ Peptide (μHLFR^{S}Aμ).

Protease	Fragment	[M+H]$^{+}$		[M+Na]$^{+}$		[M+2Na-H]$^{+}$	
		**Calculated**	**Observed**	**Calculated**	**Observed**	**Calculated**	**Observed**
**No protease** ***(Negative Control)***	μHLFR^{S}Aμ *(Intact peptide)*	1149.48	1149.40	1171.46	1171.38	1193.44	1193.35
**Cts L**	μHLFR^{S}Aμ	1149.48	1149.39	1171.46	1171.40	1193.44	1193.36
**Cts V**	μHLFR^{S}Aμ	1149.48	1149.39	1171.46	1171.51	1193.44	1193.48
**Cts K**	μHLFR^{S}Aμ	1149.48	1149.45	1171.46	1171.45	1193.44	1193.41
**Cts S**	μHLFR^{S}Aμ	1149.48	1149.38	1171.46	1171.40	1193.44	1193.39
**Cts B**	μHLFR^{S}Aμ	1149.48	1149.37	1171.46	1171.40	1193.44	1193.26

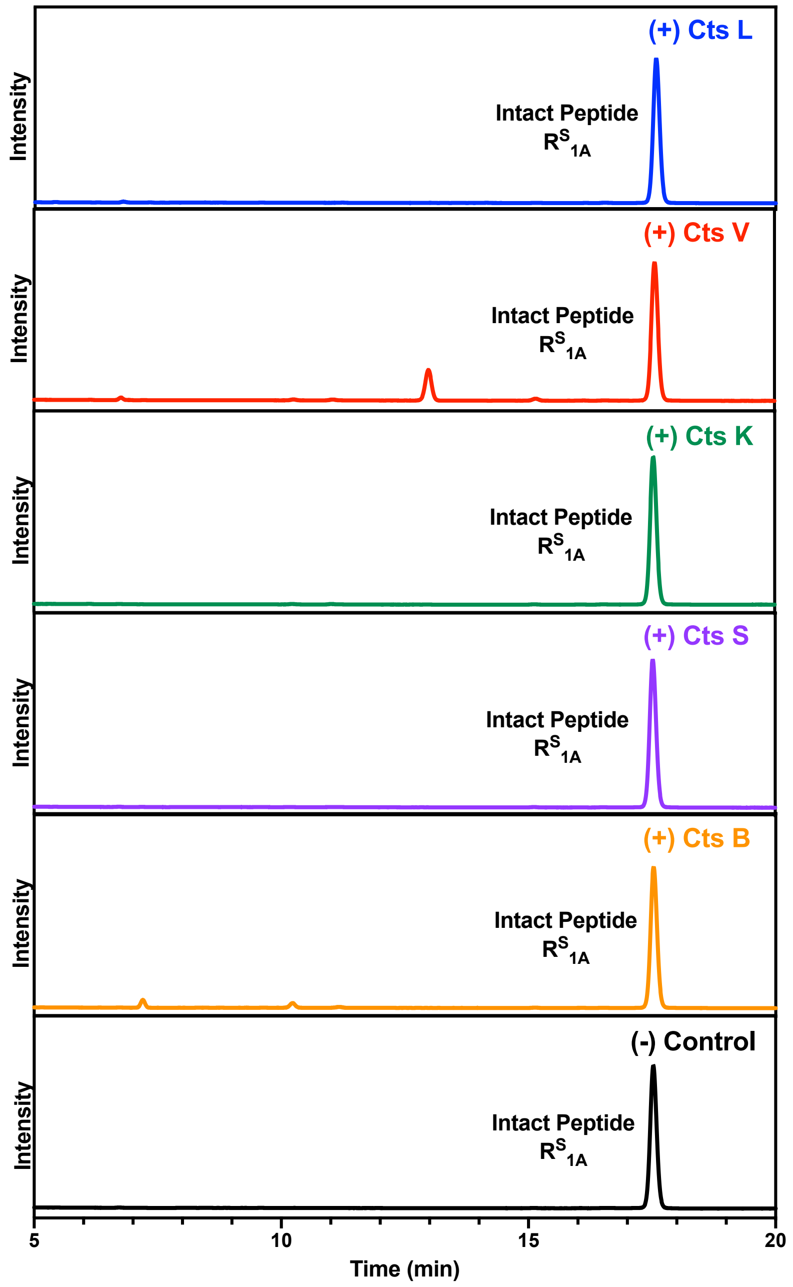

Figure S12. HPLC Analysis (325 nm) of R^{S}_{1A} Peptide (μHLFR^{S}Aμ) Cleaved by Different Cathepsins. Reaction mixtures from fluorescence assays were analyzed by HPLC after completion of the reactions.

Cathepsin L Inhibition Assay. For cathepsin L (Cts L, ≥ 0.5 units/mg protein, solution; from human liver), various concentrations of the substrate Z-FR-AMC (1 µM, 2 µM, 3 µM, 5 µM, 7 µM, 10 µM, 15 µM, and 20 µM) were reacted with 37.9 nM Cts L in a 96-well plate at 27 ºC. Assay buffer consisted of 100 mM sodium acetate, 100 mM NaCl, 1 mM EDTA, and 5 mM DTT (pH 5.5), except for the preliminary data with the µLLK^{S}AAAµ peptide (K^S$_{P1}$). Inhibition assays with this K^S$_{P1}$ peptide were conducted in 12.3 mM Na_2HPO_4, 87.7 mM NaH_2PO_4, 100 mM NaCl, 5 mM DTT, 1 mM EDTA, pH 6.0 at 27 ºC – the buffer condition used for this peptide construct in our previously published protease scanning study for thioamide positional effects (**Figure S13** & **Table S12**).[3] A stock solution of 220 µg/mL enzyme in 20 mM malonate, 1 mM EDTA, 400 mM NaCl, pH 5.5 was diluted with the assay buffer. Peptide (inhibitor) stock solutions were prepared in a minimal amount of Milli-Q water then diluted to the desired concentrations with the assay buffer. A 5 mM stock solution of the fluorogenic substrate Z-FR-AMC (Bachem; Torrance, CA, USA) was initially prepared in DMSO and diluted with assay buffer. The diluted solution of Cts L was incubated in the assay buffer with or without the peptide inhibitor at room temperature for 10 min. For each 50 µL reaction, 25 µL of this Cts L solution (either with or without the peptide inhibitor) was added to 25 µL diluted Z-FR-AMC substrate to initiate the reaction. The fluorescence of the reaction was monitored as a function of time at 460 nm with an excitation wavelength of 380 nm on the Tecan M1000 plate reader. Three independent trials were performed for each assay to ensure reproducibility. For analysis, the fluorescence data was converted to rates of proteolysis in terms of concentration (µM/min) by a conversion factor calculated from a linear fit of the endpoint fluorescence at complete hydrolysis versus substrate concentration. The initial reaction rate was then calculated by extracting the slope of a linear fit of the first 2 min of the progress curve (**Figures S13-S19**). These curves were fit to a Michaelis-Menten enzyme kinetic

model in GraphPad Prism 8.4.3 (GraphPad Software; La Jolla, CA) using **Eq. S1** to determine V_{max} and K_M (**Tables S12-S18**).[5] Turnover number (k_{cat}) was calculated by dividing V_{max} by the enzyme concentration (**Tables S12-S18**).

$$v_0 = \frac{V_{max}[S]}{K_M + [S]} \qquad \textbf{(Eq. S1)}$$

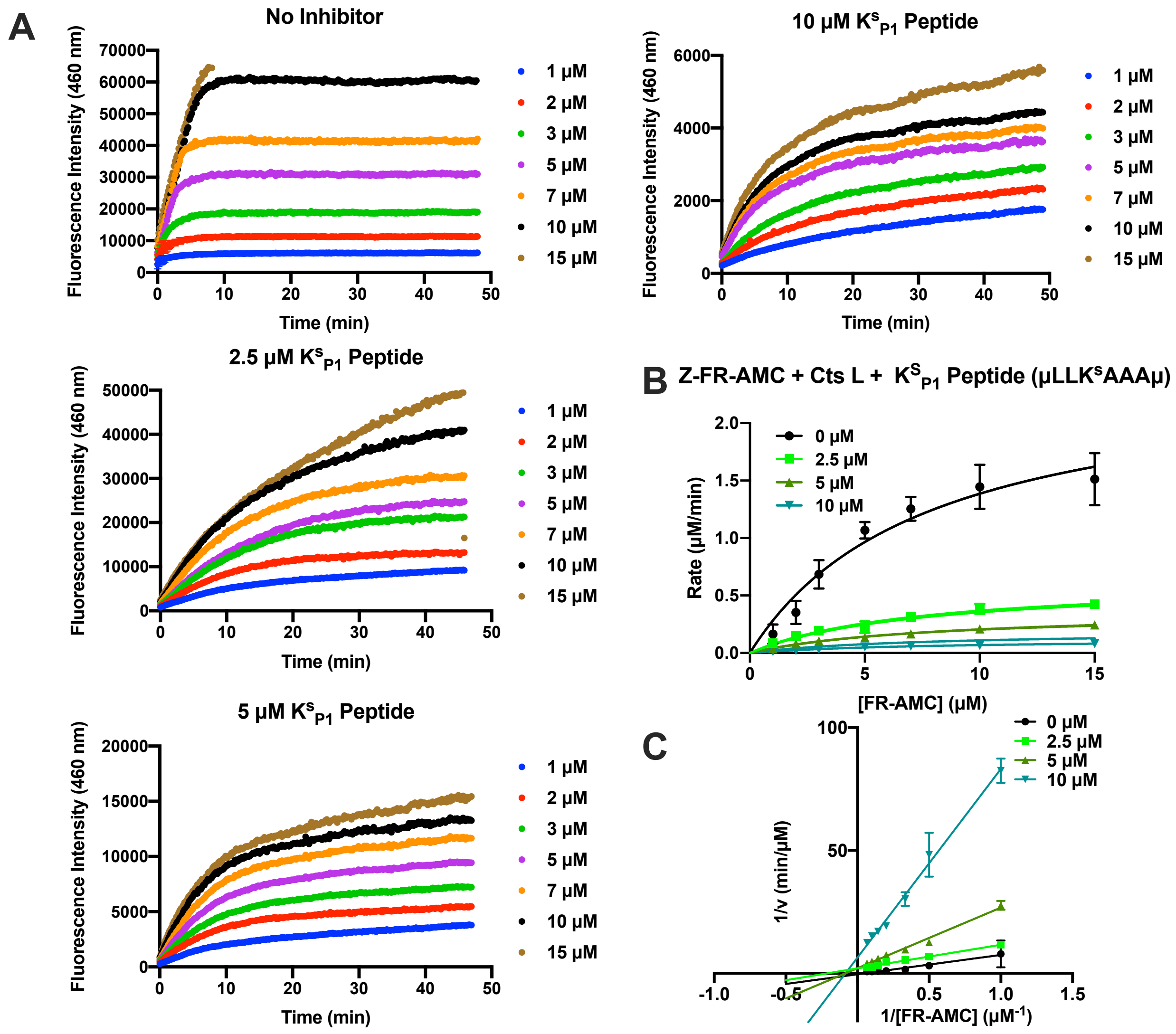

Figure S13. Preliminary data of Cts L Inhibition with K^{S}_{P1} Peptide (µLLK^{S}AAAµ). **(A)** Various concentration of Z-FR-AMC substrate were incubated with 12.3 mM Na_2HPO_4, 87.7 mM NaH_2PO_4, 100 mM NaCl, 5 mM DTT, 1 mM EDTA, pH 6.0 at 27ºC in the absence or presence of 2.5 µM, 5 µM, or 10 µM K^{S}_{P1} peptide. **(B)** Michaelis-Menten and **(C)** Lineweaver-Burk Analysis.

Table S12. Kinetic Parameters for Cts L Inhibition by K^{S}_{P1} Peptide (µLLK^{S}AAAµ).

Peptide Concentration (µM)	K_M (µM)	V_{max} (µM · min^{-1})	k_{cat} (min^{-1})	k_{cat}/K_M (µM^{-1} · min^{-1})
0	7.72 ± 1.92	2.45 ± 0.30	64.70 ± 7.92	8.38 ± 2.32
2.5	7.24 ± 1.39	0.63 ± 0.06	16.71 ± 1.54	2.31 ± 0.49
5	8.48 ± 0.69	0.38 ± 0.02	10.00 ± 0.41	1.18 ± 0.11
10	8.01 ± 0.94	0.12 ± 0.01	3.26 ± 0.19	0.41 ± 0.05

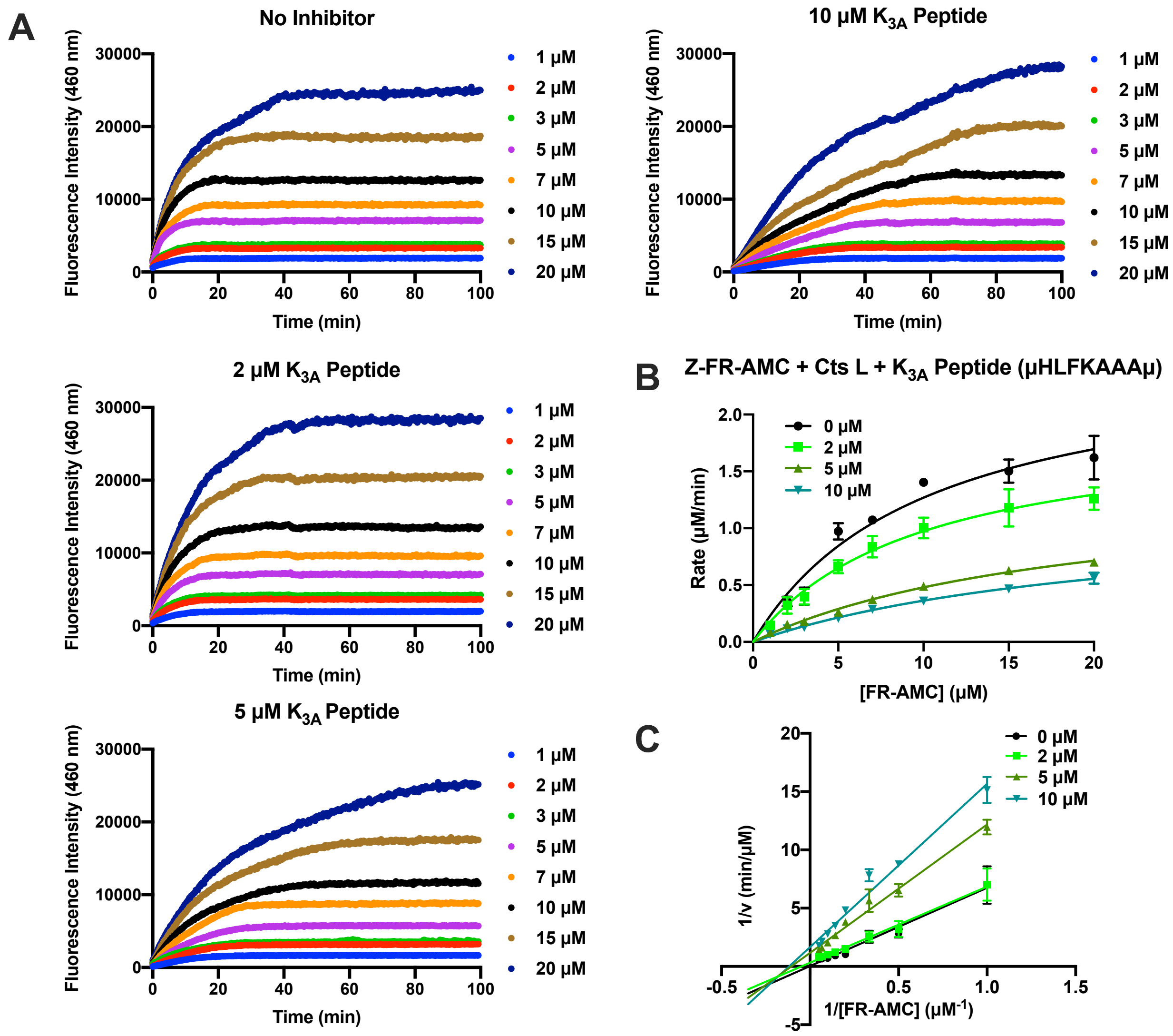

Figure S14. Cts L Proteolysis Inhibition Analysis with K_{3A} Peptide (μHLFKAAAμ). **(A)** Various concentration of Z-FR-AMC substrate were incubated with 37.9 nM Cts L in 100 mM sodium acetate, 100 mM NaCl, 1 mM EDTA, 5 mM DTT, pH 5.5 at 27ºC in the absence or presence of 2 μM, 5 μM, or 10 μM K_{3A} peptide. **(B)** Michaelis-Menten and **(C)** Lineweaver-Burk Analysis.

Table S13. Kinetic Parameters for Cts L Inhibition by K_{3A} Peptide (μHLFKAAAμ).

**Peptide Concentration (μM)**	K_M **(μM)**	V_{max} **(μM · min^{-1})**	k_{cat} **(min^{-1})**	k_{cat}/K_M **(μM^{-1} · min^{-1})**
0	10.13 ± 1.86	2.55 ± 0.23	67.33 ± 6.02	6.65 ± 1.36
2	9.86 ± 1.50	1.93 ± 0.14	50.99 ± 3.74	5.17 ± 0.87
5	19.05 ± 1.75	1.39 ± 0.08	36.75 ± 2.04	1.93 ± 0.21
10	22.40 ± 2.70	1.18 ± 0.09	31.03 ± 2.38	1.39 ± 0.20

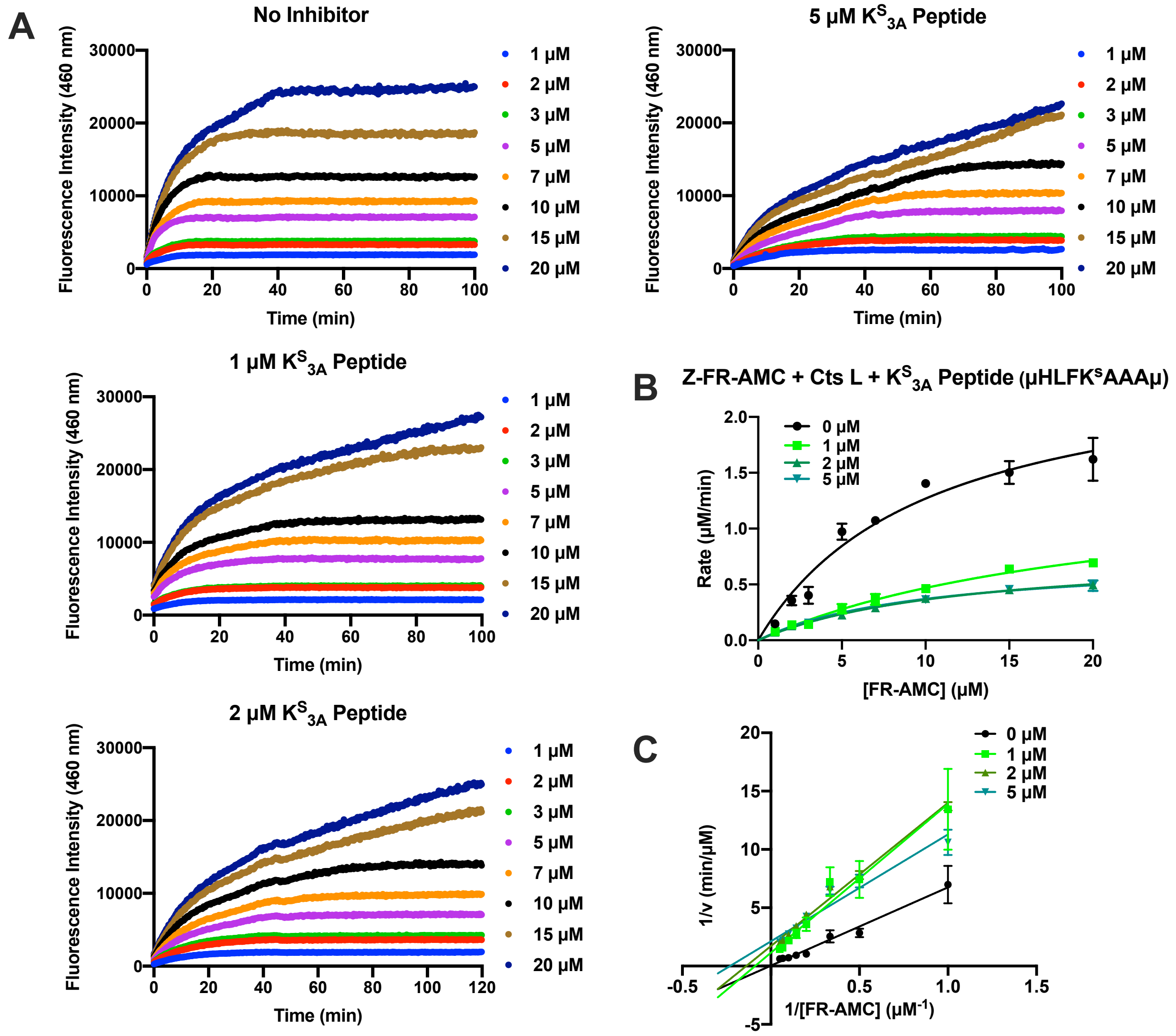

Figure S15. Cts L Proteolysis Inhibition Analysis with K^{S}_{3A} Peptide (μHLFK^{S}AAAμ). **(A)** Various concentration of Z-FR-AMC substrate were incubated with 37.9 nM Cts L in 100 mM sodium acetate, 100 mM NaCl, 1 mM EDTA, 5 mM DTT, pH 5.5 at 27ºC in the absence (previously shown in **Fig. S14**) or presence of 1 μM, 2 μM, or 5 μM K^{S}_{3A} peptide. **(B)** Michaelis-Menten and **(C)** Lineweaver-Burk Plot Analysis.

Table S14. Kinetic Parameters for Cts L Inhibition by K^{S}_{3A} Peptide (μHLFK^{S}AAAμ).

Peptide Concentration (μM)	K_M (μM)	V_{max} (μM · min^{-1})	k_{cat} (min^{-1})	k_{cat}/K_M (μM^{-1} · min^{-1})
0 (*)	10.13 ± 1.86	2.55 ± 0.23	67.33 ± 6.02	6.65 ± 1.36
1	21.76 ± 3.53	1.49 ± 0.15	39.23 ± 4.01	1.80 ± 0.35
2	12.12 ± 0.77	0.81 ± 0.03	21.26 ± 0.70	1.75 ± 0.13
5	9.95 ± 1.14	0.74 ± 0.04	19.47 ± 1.08	1.96 ± 0.25

(*) Previously shown in **Table S13**

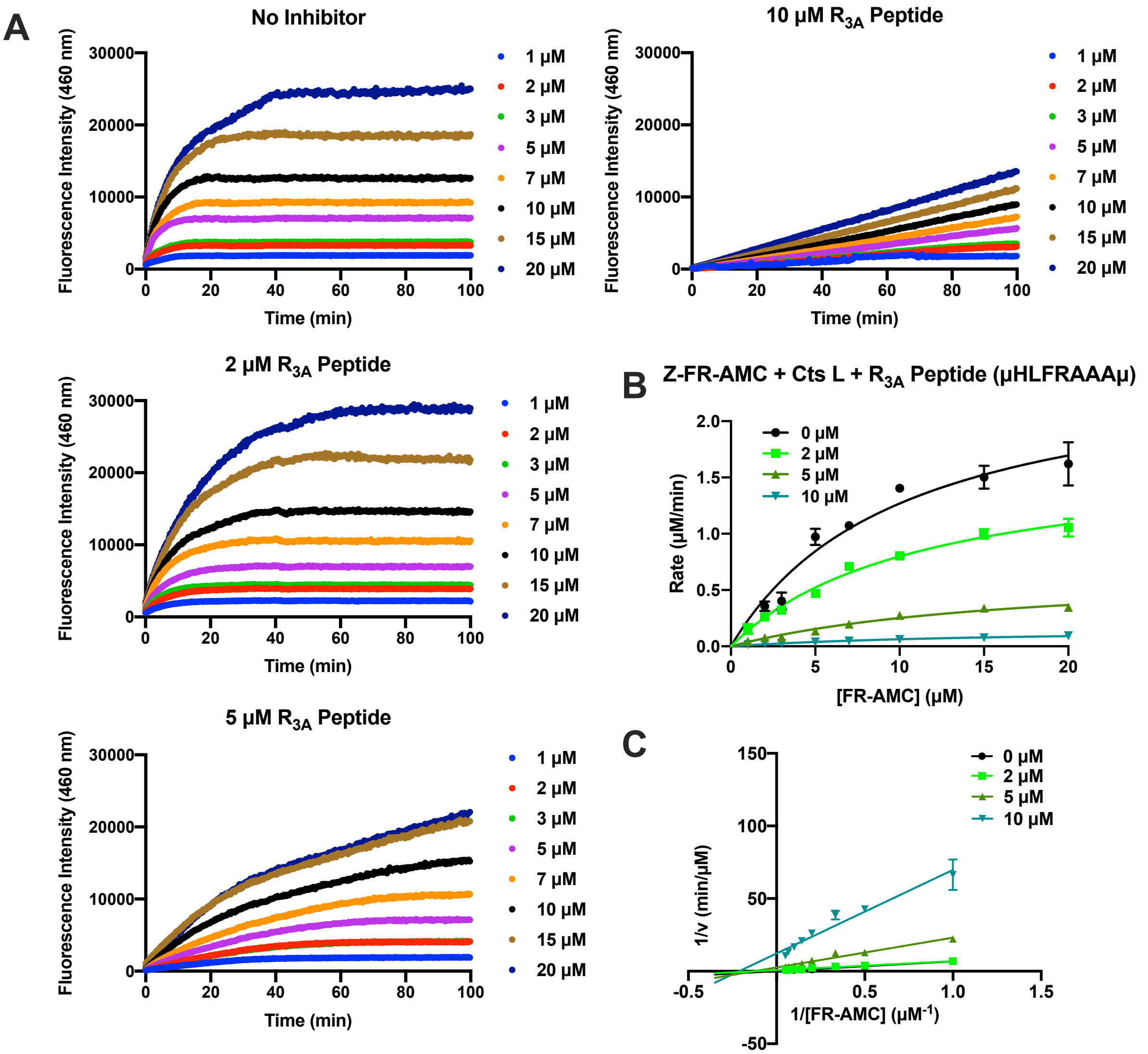

Figure S16. Cts L Proteolysis Inhibition Analysis with R_{3A} Peptide (μHLFRAAAμ). **(A)** Various concentration of Z-FR-AMC substrate were incubated with 37.9 nM Cts L in 100 mM sodium acetate, 100 mM NaCl, 1 mM EDTA, 5 mM DTT, pH 5.5 at 27ºC in the absence (previously shown in **Fig. S14**) or presence of 2 μM, 5 μM, or 10 μM R_{3A} peptide. **(B)** Michaelis-Menten and **(C)** Lineweaver-Burk Analysis.

Table S15. Kinetic Parameters for Cts L Inhibition by R_{3A} Peptide (μHLFRAAAμ).

Peptide Concentration (μM)	K_M (μM)	V_{max} (μM · min^{-1})	k_{cat} (min^{-1})	k_{cat}/K_M (μM^{-1} · min^{-1})
0 (*)	10.13 ± 1.86	2.55 ± 0.23	67.33 ± 6.02	6.65 ± 1.36
2	11.27 ± 1.26	1.70 ± 0.10	44.79 ± 2.53	3.97 ± 0.50
5	17.06 ± 2.76	0.68 ± 0.06	17.95 ± 1.69	1.05 ± 0.20
10	16.03 ± 2.08	0.16 ± 0.01	4.31 ± 0.32	0.27 ± 0.04

(*) Previously shown in **Table S13**

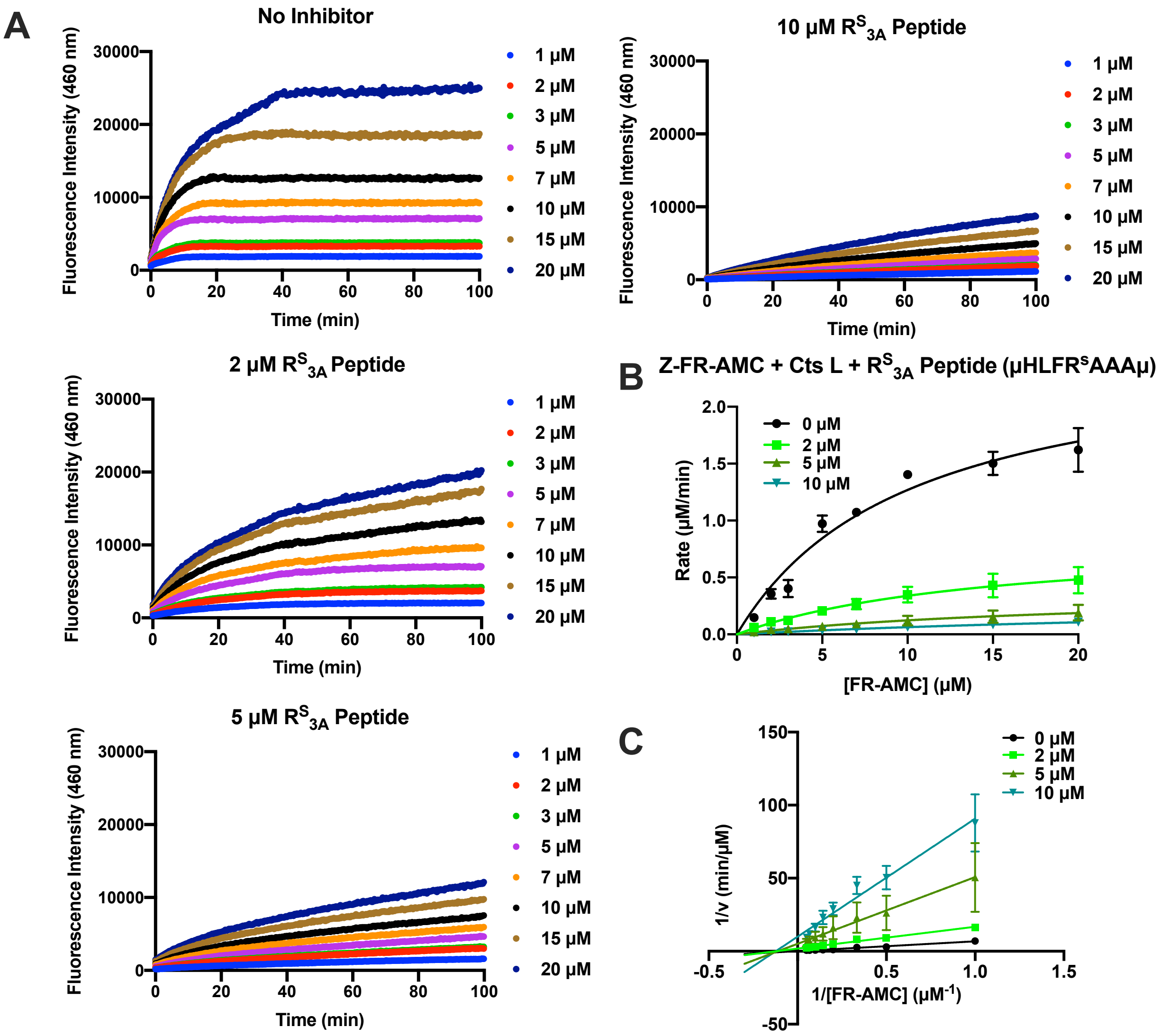

Figure S17. Cts L Proteolysis Inhibition Analysis with R^{S}_{3A} Peptide (μHLFR^{S}AAAμ). **(A)** Various concentration of Z-FR-AMC substrate were incubated with 37.9 nM Cts L in 100 mM sodium acetate, 100 mM NaCl, 1 mM EDTA, 5 mM DTT, pH 5.5 at 27ºC in the absence (previously shown in **Fig. S14**) or presence of 2 μM, 5 μM, or 10 μM R^{S}_{3A} peptide. **(B)** Michaelis-Menten and **(C)** Lineweaver-Burk Analysis.

Table S16. Kinetic Parameters for Cts L Inhibition by R^{S}_{3A} Peptide (μHLFR^{S}AAAμ).

Peptide Concentration (μM)	K_M (μM)	V_{max} (μM · min^{-1})	k_{cat} (min^{-1})	k_{cat}/K_M (μM^{-1} · min^{-1})
0 (*)	10.13 ± 1.86	2.55 ± 0.23	67.33 ± 6.02	6.65 ± 1.36
2	14.92 ± 4.62	0.84 ± 0.15	22.24 ± 3.85	1.49 ± 0.53
5	21.49 ± 12.37	0.39 ± 0.14	10.22 ± 3.69	0.48 ± 0.32
10	40.20 ± 12.73	0.32 ± 0.07	8.38 ± 1.97	0.21 ± 0.08

(*) Previously shown in **Table S13**

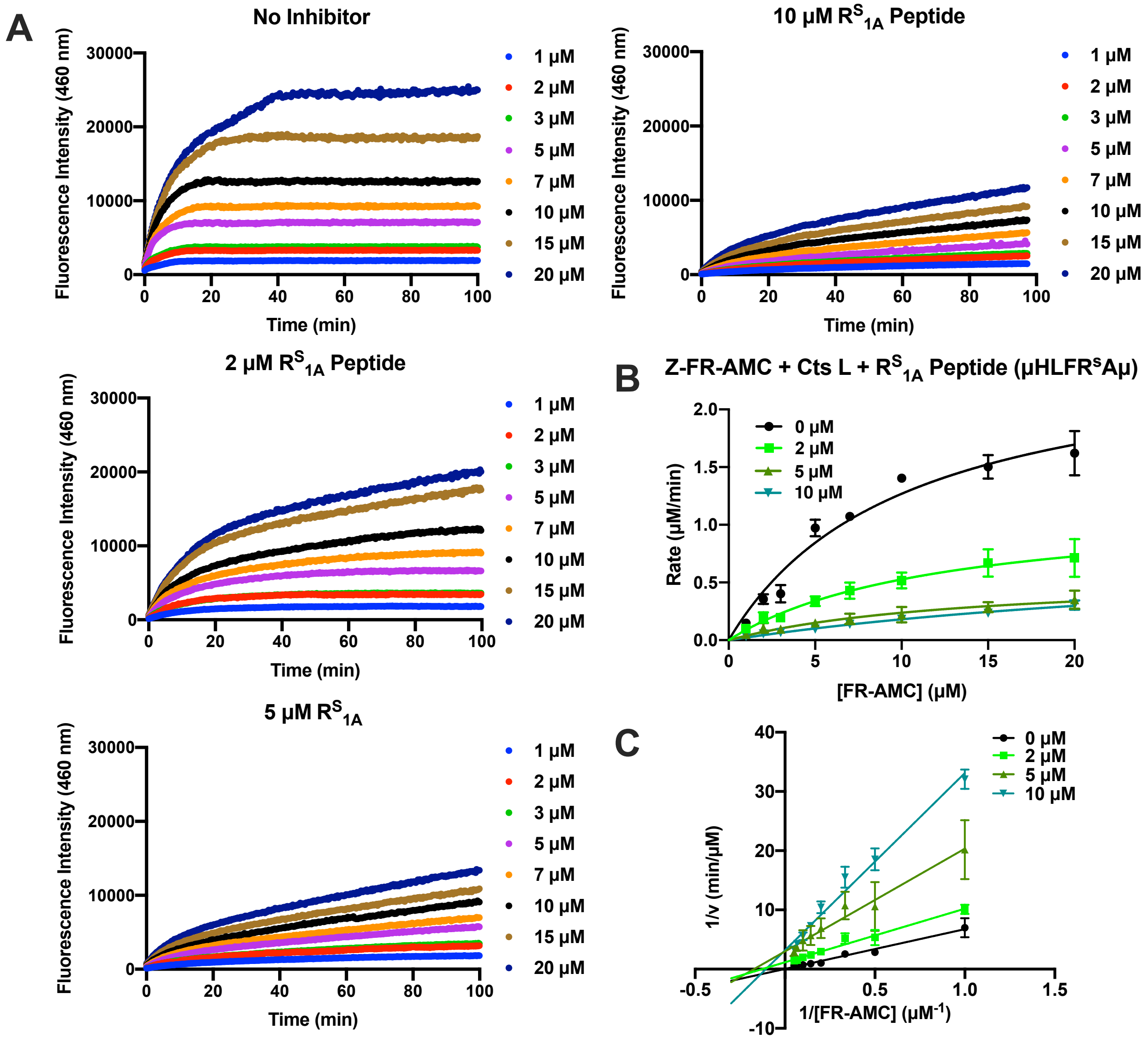

Figure S18. Cts L Proteolysis Inhibition Analysis with R^{S}_{1A} Peptide (μHLFR^{S}Aμ). **(A)** Various concentration of Z-FR-AMC substrate were incubated with 37.9 nM Cts L in 100 mM sodium acetate, 100 mM NaCl, 1 mM EDTA, 5 mM DTT, pH 5.5 at 27ºC in the absence (previously shown in **Fig. S14**) or presence of 2 μM, 5 μM, or 10 μM R^{S}_{1A} peptide. **(B)** Michaelis-Menten and **(C)** Lineweaver-Burk Analysis.

Table S17. Kinetic Parameters for Cts L Inhibition by R^{S}_{1A} Peptide (μHLFR^{S}Aμ).

**Peptide Concentration (μM)**	K_M **(μM)**	V_{max} **(μM · min^{-1})**	k_{cat} **(min^{-1})**	k_{cat}/K_M **(μM^{-1} · min^{-1})**
0 (*)	10.13 ± 1.86	2.55 ± 0.23	67.33 ± 6.02	6.65 ± 1.36
2	12.23 ± 3.14	1.17 ± 0.16	30.85 ± 4.13	2.52 ± 0.73
5	13.94 ± 4.81	0.57 ± 0.11	15.01 ± 2.83	1.08 ± 0.42
10	36.19 ± 9.44	0.84 ± 0.16	22.02 ± 4.16	0.61 ± 0.20

(*) Previously shown in **Table S13**

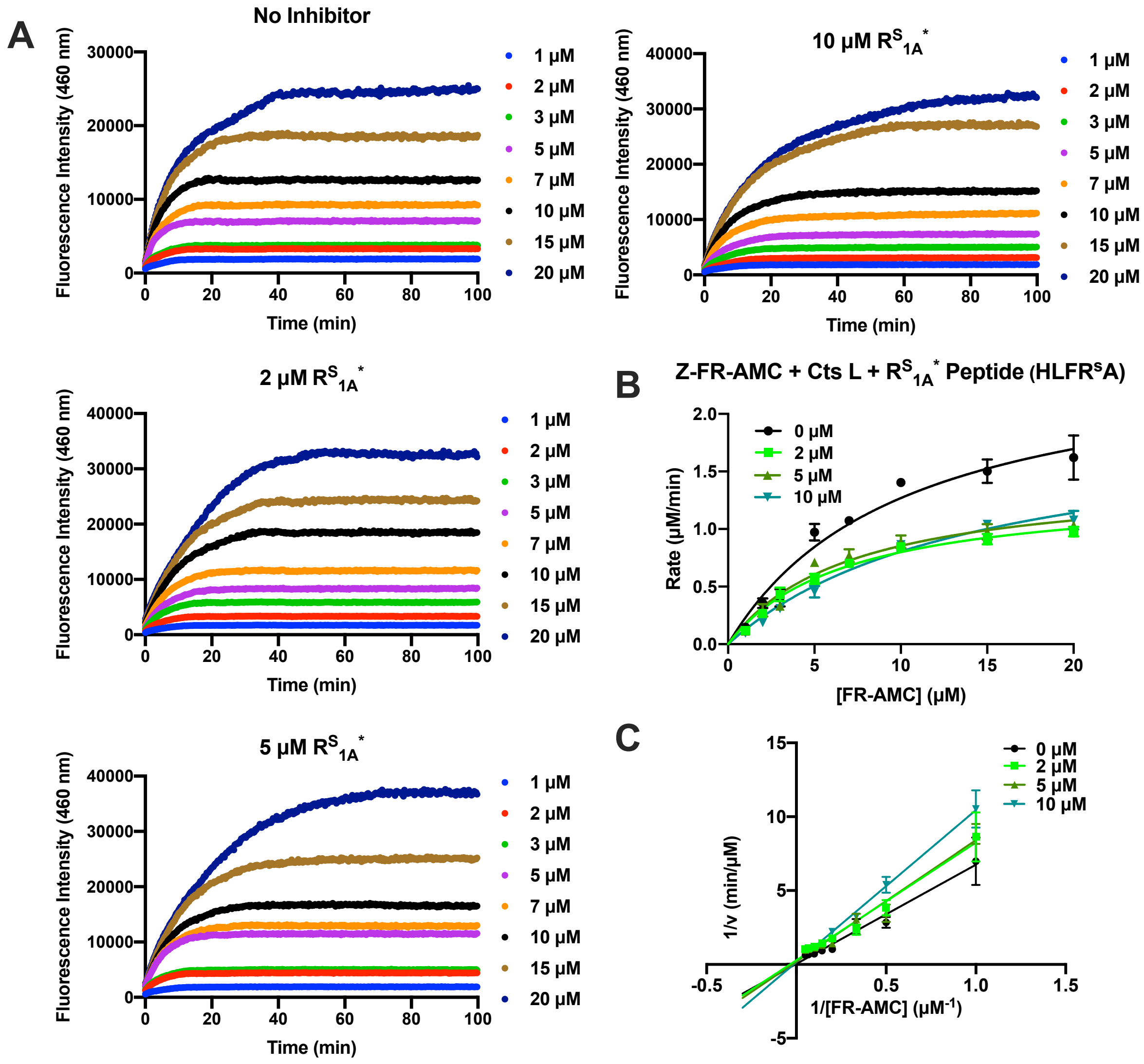

Figure S19. Cts L Proteolysis Inhibition Analysis with $R^{S}_{1A}{}^{*}$ Peptide ($HLFR^{S}A$). **(A)** Various concentration of Z-FR-AMC substrate were incubated with 37.9 nM Cts L in 100 mM sodium acetate, 100 mM NaCl, 1 mM EDTA, 5 mM DTT, pH 5.5 at 27ºC in the absence (previously shown in **Fig. S14**) or presence of 2 μM, 5 μM, or 10 μM $R^{S}_{1A}{}^{*}$ Peptide. **(B)** Michaelis-Menten and **(C)** Lineweaver-Burk Analysis.

Table S18. Kinetic Parameters for Cts L Inhibition by $R^{S}_{1A}{}^{*}$ Peptide ($HLFR^{S}A$).

Peptide Concentration (μM)	K_M (μM)	V_{max} (μM · min^{-1})	k_{cat} (min^{-1})	k_{cat}/K_M (μM^{-1} · min^{-1})
0 (*)	10.13 ± 1.86	2.55 ± 0.23	67.33 ± 6.02	6.65 ± 1.36
2	6.85 ± 0.67	1.35 ± 0.06	35.51 ± 1.46	5.18 ± 0.55
5	7.01 ± 1.06	1.45 ± 0.09	38.28 ± 2.46	5.46 ± 0.90
10	14.40 ± 2.08	1.96 ± 0.16	51.75 ± 4.12	3.59 ± 0.59

(*) Previously shown in **Table S13**

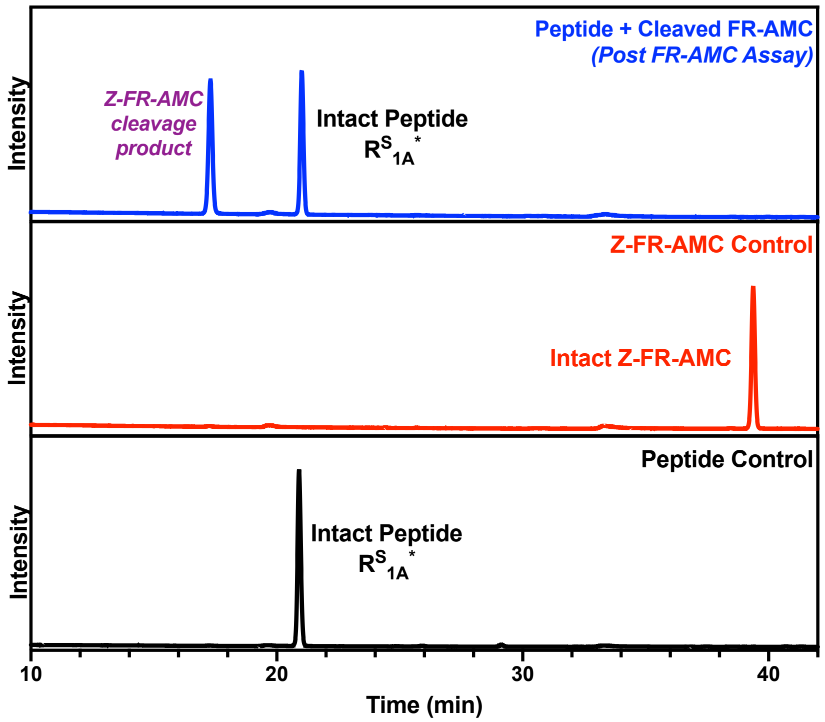

Figure S20. Representative HPLC Analysis (monitored at 275 nm) of $R^{S}_{1A}{}^{*}$ Peptide (HLFR^{S}A) at the End of the Inhibition Assay with the Substrate Z-Phe-Arg-AMC (Z-FR-AMC) and 37.9 nM Cts L. The inhibitor peptide remained intact after the 2-hour assay while the Cts L substrate (Z-FR-AMC) was completely cleaved by the protease by the end of the assay. The $R^{S}_{1A}{}^{*}$ peptide shows resistance to proteolysis; however, it does not inhibit Cts L as effectively as the R^{S}_{1A} Peptide (μHLFR^{S}Aμ).

Cathepsin V Inhibition Assay. For cathepsin V (Cts V, 0.38 mg/mL, solution; recombinant, expressed in FreeStyle™ 293-F cells, ≥90% pure by SDS-PAGE), various concentrations of the substrate Z-LR-AMC (1 µM, 3 µM, 5 µM, 7 µM, 10 µM, 15 µM, 20 µM, and 25 µM) were reacted with 19.3 nM Cts V in a 96-well plate at 27 ºC. Assay buffer consisted of 100 mM sodium acetate, 100 mM NaCl, 1 mM EDTA, and 5 mM DTT (pH 5.5). A stock solution of 0.35 mg/mL enzyme in aqueous buffer solution was diluted with the assay buffer. Peptide (inhibitor) stock solutions were prepared in a minimal amount of Milli-Q water then diluted to the desired concentrations with the assay buffer. A 20 mM stock solution of the fluorogenic substrate Z-LR-AMC (Bachem; Torrance, CA, USA) was initially prepared in DMSO and diluted with assay buffer. The diluted solution of Cts V was incubated in the assay buffer with or without the peptide inhibitor at room temperature for 10 min. For each 50 µL reaction, 25 µL of this Cts V solution (either with or without the peptide inhibitor) was added to 25 µL diluted Z-LR-AMC substrate to initiate the reaction. The fluorescence of the reaction was monitored as a function of time at 460 nm with an excitation wavelength of 380 nm on the Tecan plate reader. Three independent trials were performed for each assay to ensure reproducibility. For analysis, the fluorescence data was converted to rates of proteolysis in terms of concentration (µM/min) by a conversion factor calculated from a linear fit of the endpoint fluorescence at complete hydrolysis versus substrate concentration. The initial reaction rate was then calculated by extracting the slope of a linear fit of the first 2 min of the progress curve (**Figure S21**). Similar to the analysis with Cts L, these curves were fit to a Michaelis-Menten enzyme kinetic model in Prism 8 (GraphPad Software; La Jolla, CA) using **Eq. S1** to determine V_{max} and K_M (**Table S19**).[5] Turnover number (k_{cat}) was calculated by dividing V_{max} by the enzyme concentration (**Table S19**).

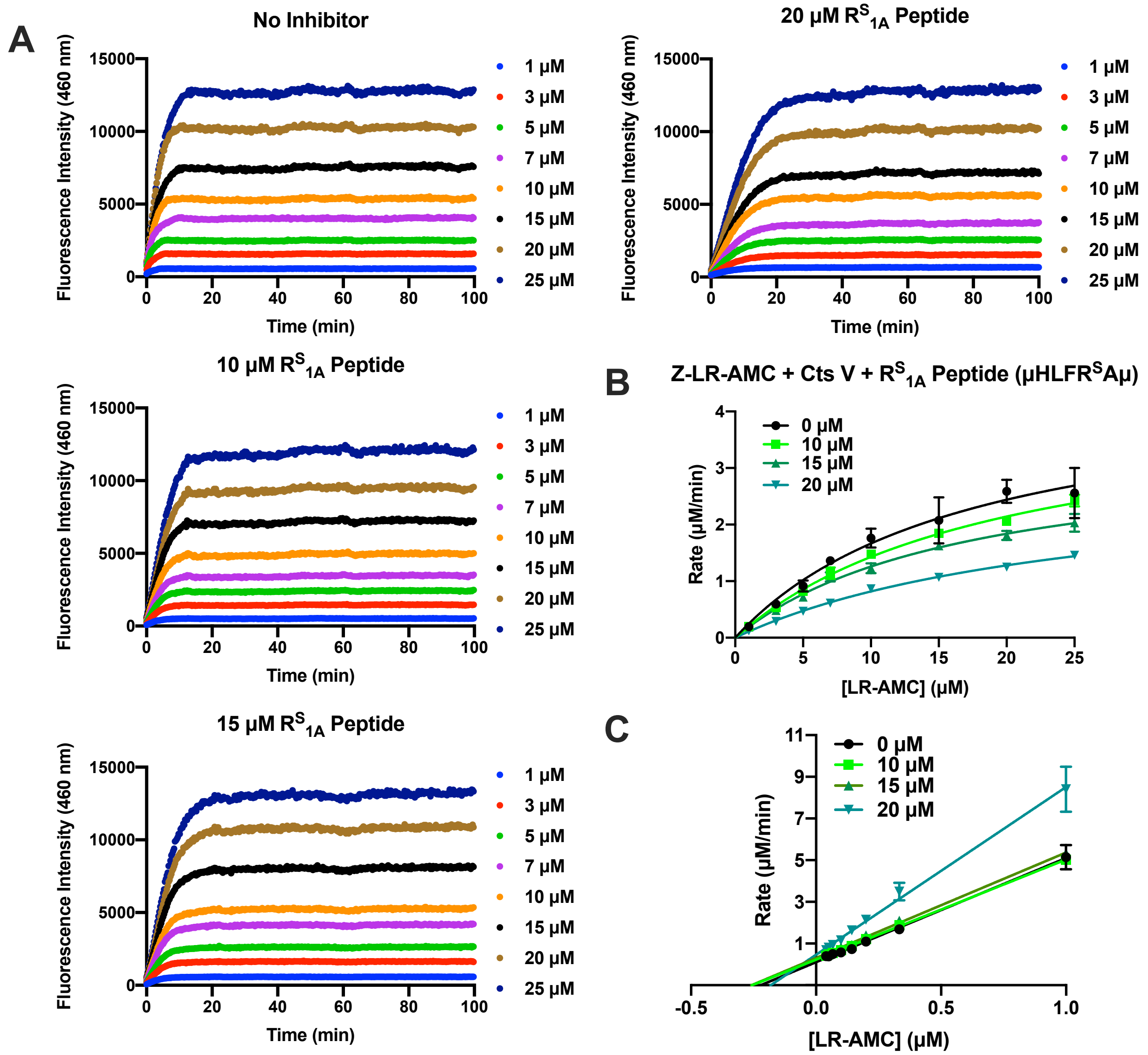

Figure S21. Cts V Proteolysis Inhibition Analysis with R^S_{1A} Peptide (μHLFR^{S}Aμ). **(A)** Various concentration of Z-LR-AMC substrate were incubated with 19.3 nM Cts V in 100 mM sodium acetate, 100 mM NaCl, 1 mM EDTA, 5 mM DTT, pH 5.5 at 27°C in the absence or presence of 10 μM, 15 μM, or 20 μM R^S_{1A} peptide. **(B)** Michaelis-Menten and **(C)** Lineweaver-Burk Analysis.

Table S19. Kinetic Parameters for Cts V Inhibition by R^S_{1A} Peptide (μHLFR^{S}Aμ).

Peptide Concentration (μM)	K_M (μM)	V_{max} (μM · min^{-1})	k_{cat} (min^{-1})	k_{cat}/K_M (μM^{-1} · min^{-1})
0	17.38 ± 3.90	4.56 ± 0.55	236.27 ± 28.35	13.59 ± 3.46
10	19.99 ± 1.64	4.28 ± 0.20	221.97 ± 10.26	11.10 ± 1.05
15	17.12 ± 1.82	3.41 ± 0.19	176.79 ± 10.01	10.33 ± 1.24
20	25.78 ± 2.46	2.93 ± 0.17	151.71 ± 8.88	5.88 ± 0.66

Cathepsin K Inhibition Assay. For cathepsin K (Cts K, 0.5 mg/mL, solution; recombinant, expressed in *E. coli*, ≥90% pure by SDS-PAGE), various concentrations of the substrate Z-LR-AMC (10 µM, 15 µM, 20 µM, 30 µM, 40 µM, 60 µM, 80 µM, and 100 µM) were reacted with 53.2 nM Cts K in a 96-well plate at 27ºC. Assay buffer consisted of 100 mM sodium acetate, 100 mM NaCl, 1 mM EDTA, and 5 mM DTT (pH 5.5). A stock solution of 0.5 mg/mL enzyme in 25 mM Na_2HPO_4 and 500 mM NaCl with 50% glycerol was diluted with the assay buffer. Peptide (inhibitor) stock solutions were prepared in a minimal amount of Milli-Q water then diluted to the desired concentrations with the assay buffer. A 20 mM stock solution of the chromogenic substrate Z-LR-AMC (Bachem; Torrance, CA, USA) was initially prepared in DMSO and diluted with assay buffer. Diluted solution of Cts K was incubated in the assay buffer with or without the peptide inhibitor at room temperature for 10 min. For each 50 µL reaction, 25 µL of this Cts K solution (either with or without the peptide inhibitor) was added to 25 µL diluted Z-LR-AMC substrate to initiate the reaction. The fluorescence of the reaction was monitored as a function of time at 460 nm with an excitation wavelength of 380 nm on the Tecan plate reader. Three independent trials were performed for each assay to ensure reproducibility. For analysis, the fluorescence data was converted to rates of proteolysis in terms of concentration (µM/min) by a conversion factor calculated from a linear fit of the endpoint fluorescence at complete hydrolysis versus substrate concentration. The initial reaction rate was then calculated by extracting the slope of a linear fit of the first 2 min of the progress curve (**Figure S22**). Similar to the analysis with Cts L, these curves were fit to Michaelis-Menten enzyme kinetic model in Prism 8 (GraphPad Software; La Jolla, CA) using **Eq. S1** to determine V_{max} and K_M (**Table S20**).[5] Turnover number (k_{cat}) was calculated by dividing V_{max} by the enzyme concentration (**Table S20**).

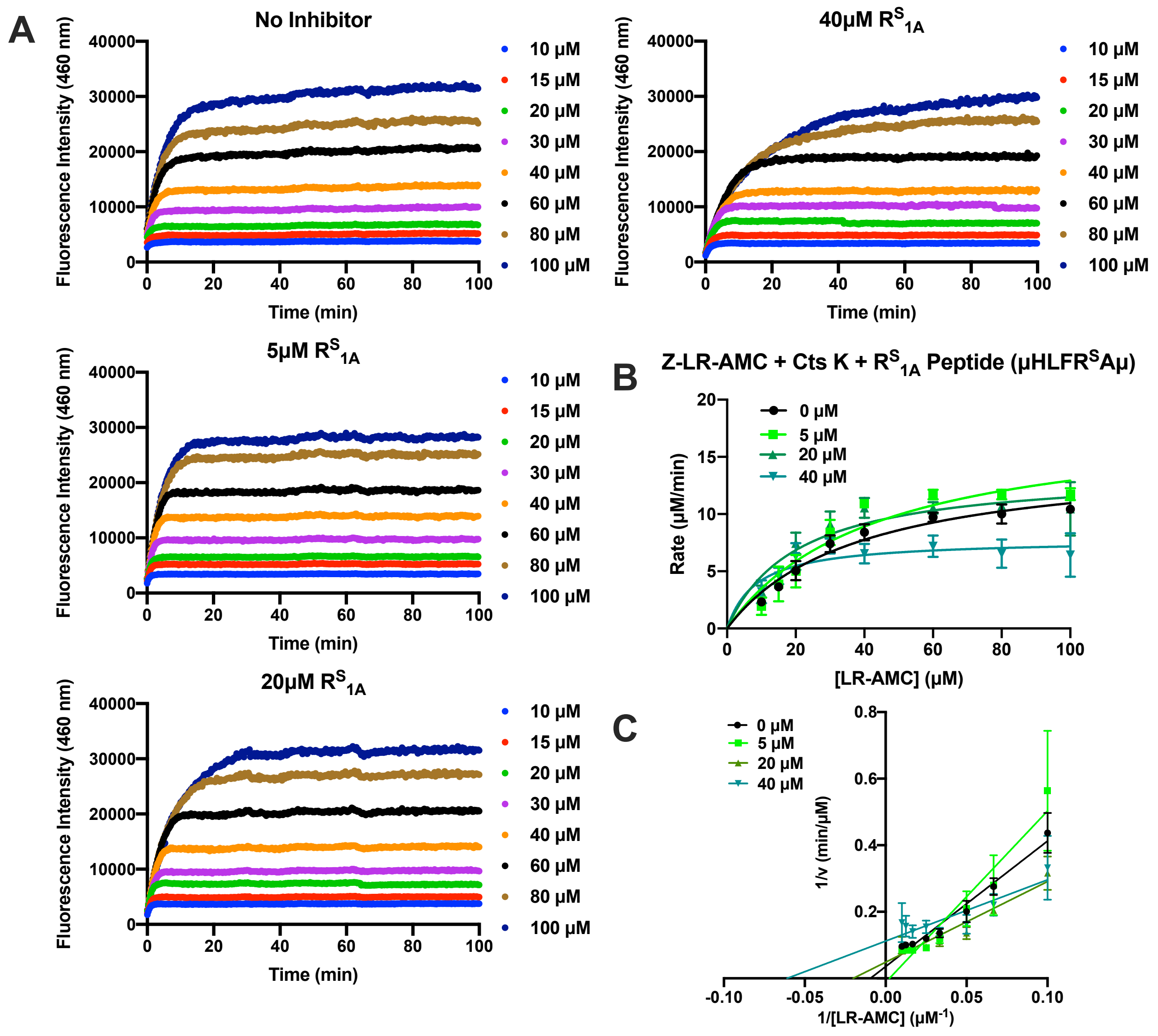

Figure S22. Cts K Proteolysis Inhibition Analysis with R^{S}_{1A} Peptide (μHLFR^{S}Aμ). **(A)** Various concentration of Z-LR-AMC substrate were incubated with 53.2 nM Cts K in 100 mM sodium acetate, 100 mM NaCl, 1 mM EDTA, 5 mM DTT, pH 5.5 at 27ºC in the absence or presence of 5 μM, 20 μM, or 40 μM R^{S}_{1A} peptide. **(B)** Michaelis-Menten and **(C)** Lineweaver-Burk Analysis.

Table S20. Kinetic Parameters for Cts K Inhibition by R^{S}_{1A} Peptide (μHLFR^{S}Aμ).

Peptide Concentration (μM)	K_M (μM)	V_{max} (μM · min^{-1})	k_{cat} (min^{-1})	k_{cat}/K_M (μM^{-1} · min^{-1})
0	37.98 ± 5.73	15.12 ± 0.99	284.21 ± 18.57	7.48 ± 1.23
5	42.91 ± 10.38	18.47 ± 2.03	347.18 ± 38.14	8.09 ± 2.15
20	20.33 ± 4.52	13.80 ± 1.03	259.40 ± 19.30	12.76 ± 2.99
40	8.51 ± 3.11	7.78 ± 0.62	146.30 ± 11.67	17.18 ± 6.42

Cathepsin S Inhibition Assay. For cathepsin S (Cts S, 2 mg/mL, solution; recombinant, expressed in FreeStyle™ 293-F cells, ≥90% pure by SDS-PAGE), various concentrations of the substrate Z-LR-AMC (15 μM, 20 μM, 25 μM, 30 μM, 40 μM, 60 μM, 80 μM, and 100 μM) were reacted with 33.8 nM Cts S in a 96-well plate at 27ºC. Assay buffer consisted of 100 mM sodium acetate, 100 mM NaCl, 1 mM EDTA, and 5 mM DTT (pH 5.5). A stock solution of 2 mg/mL enzyme in 45 mM Tris-HCl, pH 8.0, 124 mM NaCl, 2.4 mM KCl, 225 mM imidazole, 3 mM DTT, and 10% glycerol was diluted with assay buffer containing 5 mM DTT. Peptide (inhibitor) stock solutions were prepared in a minimal amount of Milli-Q water then diluted to the desired concentrations with the assay buffer. A 20 mM stock solution of the chromogenic substrate Z-LR-AMC (Bachem; Torrance, CA, USA) was initially prepared in DMSO and diluted with assay buffer. Diluted solution of Cts S was incubated in the assay buffer with or without the peptide inhibitor at 37ºC for 10 min. For each 50 μL reaction, 25 μL of this Cts S solution (either with or without the peptide inhibitor) was added to 25 μL diluted Z-LR-AMC substrate to initiate the reaction. The fluorescence of the reaction was monitored as a function of time at 460 nm with an excitation wavelength of 380 nm on the Tecan plate reader. Three independent trials were performed for each assay to ensure reproducibility. For analysis, the fluorescence data was converted to rates of proteolysis in terms of concentration (μM/min) by a conversion factor calculated from a linear fit of the endpoint fluorescence at complete hydrolysis versus substrate concentration. The initial reaction rate was then calculated by extracting the slope of a linear fit of the first 20 min of the progress curve (**Figure S23**). Similar to the analysis with Cts L, these curves were fit to Michaelis-Menten enzyme kinetic model in Prism 8 (GraphPad Software; La Jolla, CA) using **Eq. S1** to determine V_{max} and K_M (**Table S21**).[5] Turnover number (k_{cat}) was calculated by dividing V_{max} by the enzyme concentration (**Table S21**).

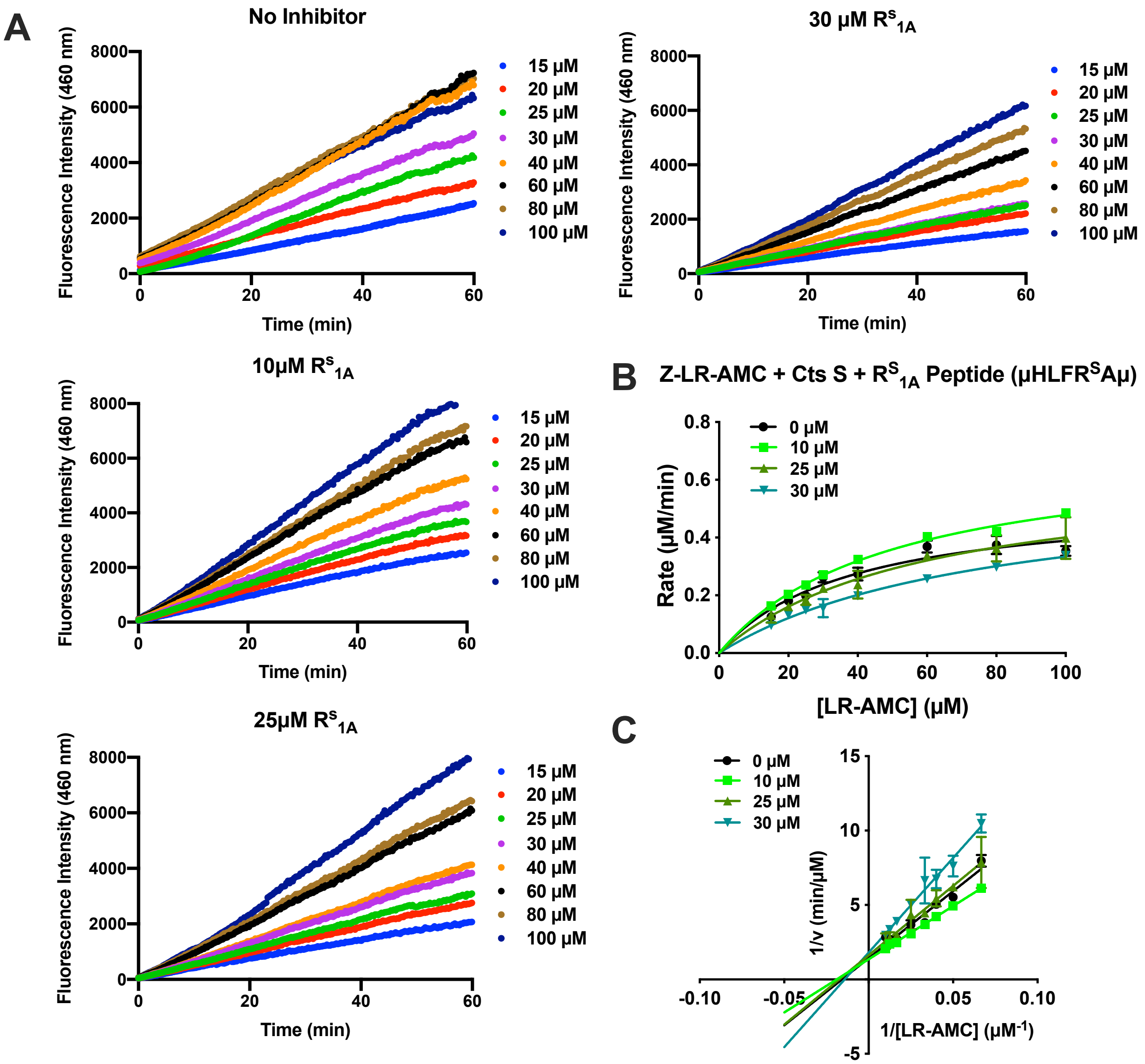

Figure S23. Cts S Proteolysis Inhibition Analysis with R$^S_{1A}$ Peptide (μHLFR^{S}Aμ). **(A)** Various concentration of Z-LR-AMC substrate were incubated with 33.8 nM Cts S in 100 mM sodium acetate, 100 mM NaCl, 1 mM EDTA, 5 mM DTT, pH 5.5 at 27°C in the absence or presence of 10 μM, 25 μM, or 30 μM R$^S_{1A}$ peptide. **(B)** Michaelis-Menten and **(C)** Lineweaver-Burk Analysis.

Table S21. Kinetic Parameters for Cts S Inhibition by R$^S_{1A}$ Peptide (μHLFR^{S}Aμ).

Peptide Concentration (μM)	K_M (μM)	V_{max} (μM · min^{-1})	k_{cat} (min^{-1})	k_{cat}/K_M (μM^{-1} · min^{-1})
0	37.68 ± 5.95	0.54 ± 0.04	15.85 ± 1.09	0.42 ± 0.07
10	49.79 ± 2.82	0.72 ± 0.02	21.19 ± 0.58	0.43 ± 0.03
25	59.58 ± 11.46	0.64 ± 0.06	18.91 ± 1.89	0.32 ± 0.07
30	78.02 ± 8.22	0.59 ± 0.04	17.59 ± 1.06	0.23 ± 0.03

Cathepsin B Inhibition Assay. For cathepsin B (Cts B, ≥1,500 units/mg protein, solution; from human liver), various concentrations of the substrate Z-RR-AMC (40 μM, 100 μM, 200 μM, 400 μM, 600 μM, 800 μM, 1000 μM, and 1200 μM) were reacted with 40.9 nM Cts B in a 96-well plate at 27 ºC. Assay buffer consisted of 100 mM sodium acetate, 100 mM NaCl, 1 mM EDTA, and 5 mM DTT (pH 5.5). A stock solution of enzyme in 50 mM sodium acetate, pH 5.0, with 1 mM EDTA was diluted with the assay buffer. Peptide (inhibitor) stock solutions were prepared in a minimal amount of Milli-Q water then diluted to the desired concentrations with the assay buffer. A 20 mM stock solution of the fluorogenic substrate Z-RR-AMC (Bachem; Torrance, CA, USA) was initially prepared in DMSO and diluted with assay buffer. Diluted solution of Cts B was incubated in the assay buffer with or without the peptide inhibitor at 37 ºC for 10 min. For each 50 μL reaction, 25 μL of this Cts B solution (either with or without the peptide inhibitor) was added to 25 μL diluted Z-RR-AMC substrate to initiate the reaction. The fluorescence of the reaction was monitored as a function of time at 460 nm with an excitation wavelength of 380 nm on the Tecan M1000 plate reader. Three independent trials were performed for each assay to ensure reproducibility. For analysis, the fluorescence data was converted to rates of proteolysis in terms of concentration (μM/min) by a conversion factor calculated from a linear fit of the endpoint fluorescence at complete hydrolysis versus substrate concentration. The initial reaction rate was then calculated by extracting the slope of a linear fit of the first 2 min of the progress curve (**Figure S24**). Similar to the analysis with Cts L, these curves were fit to a Michaelis-Menten enzyme kinetic model in Prism 8 (GraphPad Software; La Jolla, CA) using **Eq. S1** to determine V_{max} and K_M (**Table S22**).[5] Turnover number (k_{cat}) was calculated by dividing V_{max} by the enzyme concentration (**Table S22**).

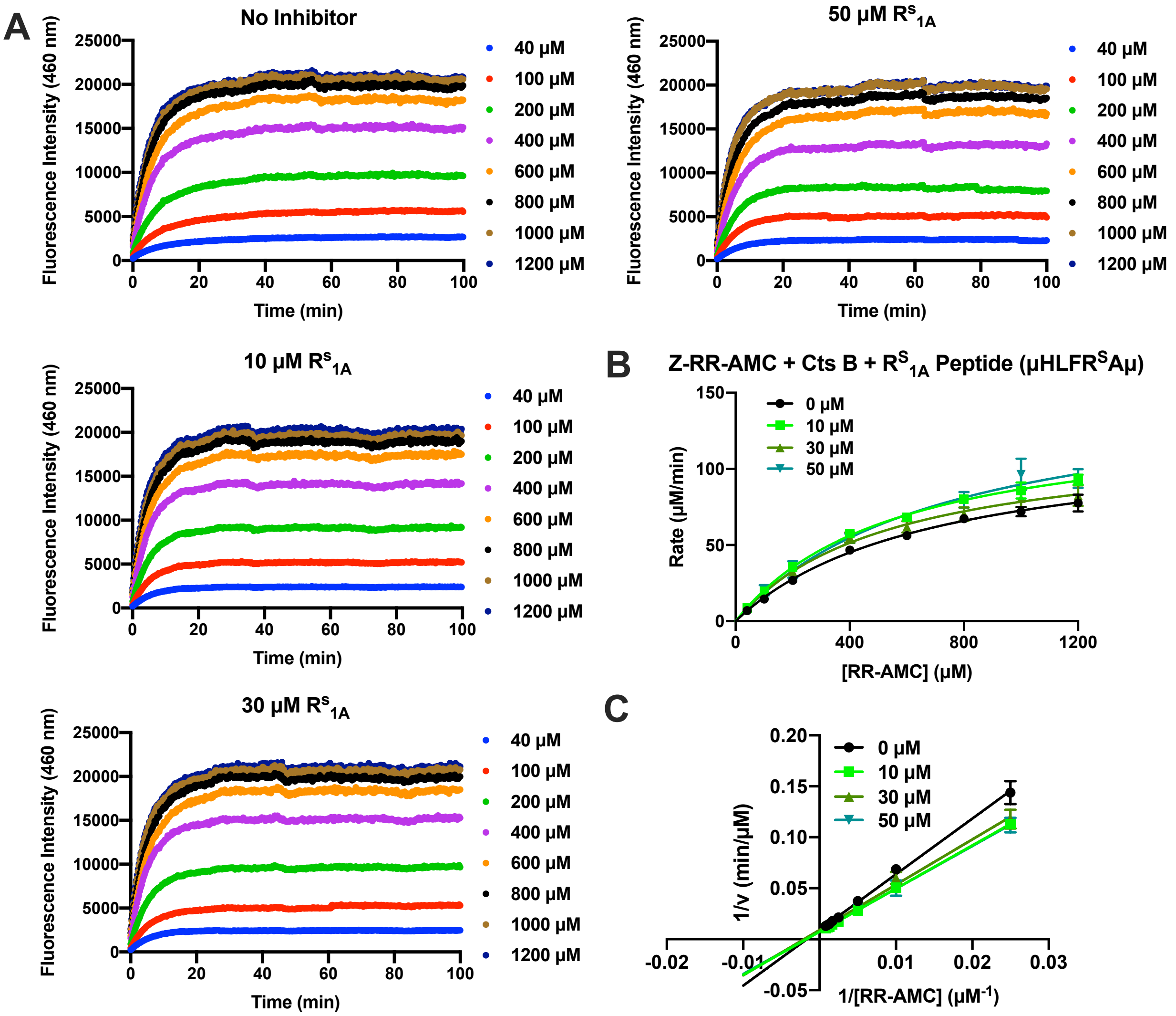

Figure S24. Cts B Proteolysis Inhibition Analysis with R$^S{}_{1A}$ Peptide (μHLFR^{S}Aμ). **(A)** Various concentration of Z-RR-AMC substrate were incubated with 40.9 nM Cts B in 100 mM sodium acetate, 100 mM NaCl, 1 mM EDTA, 5 mM DTT, pH 5.5 at 27ºC in the absence or presence of 10 μM, 30 μM, or 50 μM R$^S{}_{1A}$ peptide. **(B)** Michaelis-Menten and **(C)** Lineweaver-Burk Analysis.

Table S22. Kinetic Parameters for Cts B Inhibition by R$^S{}_{1A}$ Peptide (μHLFR^{S}Aμ).

Peptide Concentration (μM)	K_M (μM)	V_{max} (μM · min^{-1})	k_{cat} (min^{-1})	k_{cat}/K_M (μM^{-1} · min^{-1})
0	684.97 ± 56.82	122.39 ± 4.85	2992.42 ± 118.46	4.37 ± 0.40
10	566.77 ± 37.68	135.78 ± 3.98	3319.80 ± 97.24	5.86 ± 0.43
30	537.81 ± 63.32	120.62 ± 6.12	2949.14 ± 149.59	5.48 ± 0.70
50	742.41 ± 117.46	156.45 ± 12.21	3825.18 ± 298.56	5.15 ± 0.91

Kinetics Analysis of the Peptide Inhibitors of Cathepsin L, V, K, B, and S

For Cts L, from initial evaluation of the kinetic parameters obtained from fitting to Michaelis-Menten model (**Tables S13-18**) and Lineweaver-Burk plots (**Figs. S14-19**), there was a decrease in V_{max} but an increase or no significant change in K_M as the concentration of the inhibitors was increased. This suggested that the peptides are likely mixed-type inhibitors (**Fig. S25**). Kinetics data from Z-FR-AMC inhibition assays with Cts L was thus further fitted to a general Mixed Inhibition Model in GraphPad Prism version 8.4.3 (GraphPad Software; La Jolla, CA), using **Equations S2-S4**.[5]

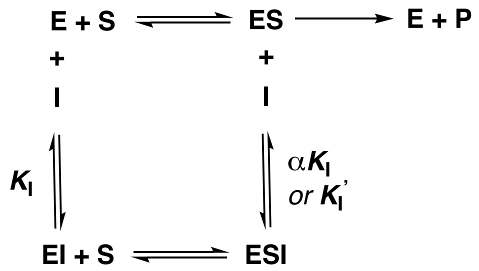

Figure S25. A mixed Inhibition Mechanism for Reversible Inhibitors. Abbreviations: E: enzymes; S: substrate; I: inhibitor; P: product; EI: enzyme-inhibitor complex; ESI: enzyme-substrate-inhibitor complex; K_I: dissociation constant for the EI complex; αK_I (also known as K_I'): dissociation constant for the ESI complex.

$$K_{M,app} = \frac{K_M(1+\frac{[I]}{K_I})}{1+\frac{[I]}{\alpha K_I}} \quad \textbf{(Eq. S2)}$$

$$V_{max,app} = \frac{V_{max}}{1+\frac{[I]}{\alpha K_I}} \quad \textbf{(Eq. S3)}$$

$$V_0 = \frac{V_{max,app}[S]}{K_{M,app}+[S]} \quad \textbf{(Eq. S4)}$$

This general mixed-inhibition model uses a global fitting in which V_{max}, K_M, K_I, constant α and are shared. The constant α here determines the degree to which inhibitor binding changes the affinity of the enzyme for substrate; the value of α is always greater than zero.[5, 6] An $\alpha = 1$ ($K_I = \alpha K_I$) suggests that there is no change in the affinity of the enzyme for substrate upon binding of the inhibitor or the inhibitor has the same affinity for the enzyme (E) and the enzyme-substrate complex (ES); the model then becomes identical to a noncompetitive inhibition model.[5, 6] When $\alpha > 1$, the inhibitor binds preferentially to the free enzyme (E); if $\alpha = \infty$, the inhibitor binds almost exclusively the free enzyme and this model becomes identical to competitive inhibition.[5, 6] If $0 < \alpha < 1$, the inhibitor binds preferentially to the enzyme-substrate complex (ES) and the model approaches an uncompetitive model.[5, 6] The kinetic parameters resulted from fitting to this model for our peptides are summarized in **Table S23** (or **Table 2** of the main text). All of the values for α were close to and larger than 1, thereby suggesting these peptides are likely to be mixed-type inhibitors of Cts L with element of competitive inhibition since a value of $\alpha > 1$ suggests tighter binding to the free enzyme. This was also consistent with the aforementioned preliminary evaluation of the kinetic parameters obtained from fitting to Michaelis-Menten model (**Tables S13-18**) and Lineweaver-Burk plots (**Figs. S14-19**).

Table S23. Kinetic Parameters for Cts L Inhibition by Peptides.

Peptide	Sequence	α	K_I (μM)	αK_I or K_I' (μM)
K_{3A}	μHLFKAAAμ	1.91 ± 0.92	3.05 ± 0.64	5.81 ± 3.06
K^S_{3A}	μHLFK^{S}AAAμ	2.18 ± 1.35	0.60 ± 0.15	1.30 ± 0.87
R_{3A}	μHLFRAAAμ	1.33 ± 0.78	1.68 ± 0.49	2.23 ± 1.47
R^S_{3A}	μHLFR^{S}AAAμ	1.83 ± 0.99	0.52 ± 0.12	0.95 ± 0.56
R^S_{1A}	μHLFR^{S}Aμ	1.61 ± 0.69	1.11 ± 0.22	1.79 ± 0.85
R^S_{1A}'	HLFR^{S}A	1.71 ± 1.89	13.23 ± 6.89	22.58 ± 27.61

Similar analysis was done for Cts V, Cts K, Cts S, and Cts B to evaluate and approximate the inhibitory effects of the thiopeptide R^{S}_{1A}. Initial evaluation of the kinetic parameters were obtained from fitting to Michaelis-Menten model (**Tables S19-22**) and Lineweaver-Burk plots (**Figs. S21-24**), then the data were further analyzed with the non-linear regression method with a mixed-inhibition model in GraphPad Prism version 8.4.3 (GraphPad Software; La Jolla, CA) to determine the mechanism with the parameter α. Further fitting to an appropriate model (*e.g.* uncompetitive, competitive, or non-competitive model) was performed if needed. The approximate kinetic parameters resulted from best fit models for the R^{S}_{1A} peptide and the corresponding proteases are summarized in **Table S24**.

Table S24. Approximate Kinetic Parameters for Cts L, V, K, S, and B Inhibition by R^{S}_{1A} Peptide (μHLFR^{S}Aμ).

Protease	α (Best Fitted Model)	K_I (μM)	αK_I or K_I' (μM)
Cts L	1.61 (Mixed)	1.11 ± 0.22	1.79 ± 0.85
Cts V	1.55 (Mixed)	26.22 ± 8.42	40.64 ± 34.91
Cts K	0.01 (Uncompetitive)	-	85.00 ± 21.87
Cts S	Very large (Competitive)	53.47 ± 11.42	-
Cts B	Very large (Competitive)	>> 50	-

Cathepsin L (Cts L) Activity Assay with HepG2 Whole Cell Lysate

Cts L activity in human hepatocellular carcinoma HepG2 whole cell lysate (200 µg at 2.5 mg/ml; ab166833; Abcam, Cambridge, MA, USA) was evaluated using a fluorometric Cathepsin L Activity Assay Kit (ab65306; Abcam) following the manufacturer's protocols. Briefly, in each well of a 96-well plate, 50 µL of the HepG2 cell lysate diluted in the manufacturer's CL buffer (to a final concentration of 0.05 mg/mL HepG2) was incubated with 50 µL of CL buffer without (Control) or with different concentrations (15 µM, 20 µM, 30 µM, 50 µM, 70 µM, 80 µM, 100 µM, and 120 µM) of the peptide inhibitor R^{S}_{1A}. Different concentrations (56 nM, 560 nM, and 1 µM) of SID 26681509, a known Cts L inhibitor, were used as the positive control.[7] The cell lysate and the peptide inhibitor were incubated at room temperature for 10 min. A total of 2 µL of 10mM CL substrate Ac-FR-AFC substrate (to a final concentration of 200 µM) was then added to each well, except the Lysate Background Control wells. The samples were mixed and the plate was sealed to avoid evaporation and incubated at 37 ºC for 1 h. The fluorescence of each sample was measured at 505 nm with an excitation wavelength of 400 nm on the Tecan M1000 plate reader. The fluorescence data shown in **Figure 4** in the main text are the average of three replicates. The Lysate Background Controls indicated that there was no significant inherent fluorescence at 505 nm from the cell lysate mixture. Representative Cts L activity data (% of the Control's fluorescence signal) in HepG2 whole cell lysate was fitted to a dose-response curve equation in Prism, [Inhibitor] vs. response (three parameters), to obtain the IC_{50} value.[5]

Stability of Peptides in HepG2 Whole Cell Lysate

Using the same conditions as the previous Cts L Activity Assay in HepG2 Whole Cell Lysate, 50 µL peptide (either R_{1A} or R^{S}_{1A}; final concentration 20 µM) was incubated in 50 µL HepG2 whole cell lysate (to a final concentration of 0.05 mg/mL) in the assay buffer for different time points. The all-amide peptide, R_{1A}, was incubated for t = 0, 5, 10, 15, 20, and 30 minutes. The thioamide peptide, R^{S}_{1A}, was incubated for t = 0, 2, 6, 8, 10, and 24 hours. After incubation for the desired amount of time, 100 µL methanol was added to the mixture and incubated at -20ºC for 10 minutes before spinning down at 13,000 rpm for 10 minutes. Then, 180 µL of each mixture was further diluted with 220 µL Milli-Q water to a total volume of 400 µL before adding 1 µL 0.42 mM coumarin internal standard for HPLC analysis. The coumarin internal standard was used to normalize the amount of intact peptide in each sample. A Phenomenex Luna® Omega 5 µm PS C18 100 Å analytical column was used to analyze all samples using the same gradient of 20-40 % B over 20 minutes (Solvent A: 0.1% TFA in Milli-Q water; Solvent B: 0.1% TFA in acetonitrile). Representative HPLC spectra (325 nm) at different time points were also shown to confirm the stability of the peptides in HepG2 whole cell lysate (**Figure S26 A & B**). MALDI MS was used to confirm the mass from each fraction from the HPLC from experiment with the all-amide peptide (R_{1A} – expected $[M+H]^+$ = 1133.51 m/z) and the thioamide peptide (R^{S}_{1A} – expected $[M+H]^+$ = 1149.48 m/z). To calculate the percent intact peptide for each sample, the average ratio of intact peptide to internal standard was compared to that ratio at t = 0. For each time point, the experiment was done in triplicates. The percent of intact peptides is shown in **Figure 4C** for both peptides.

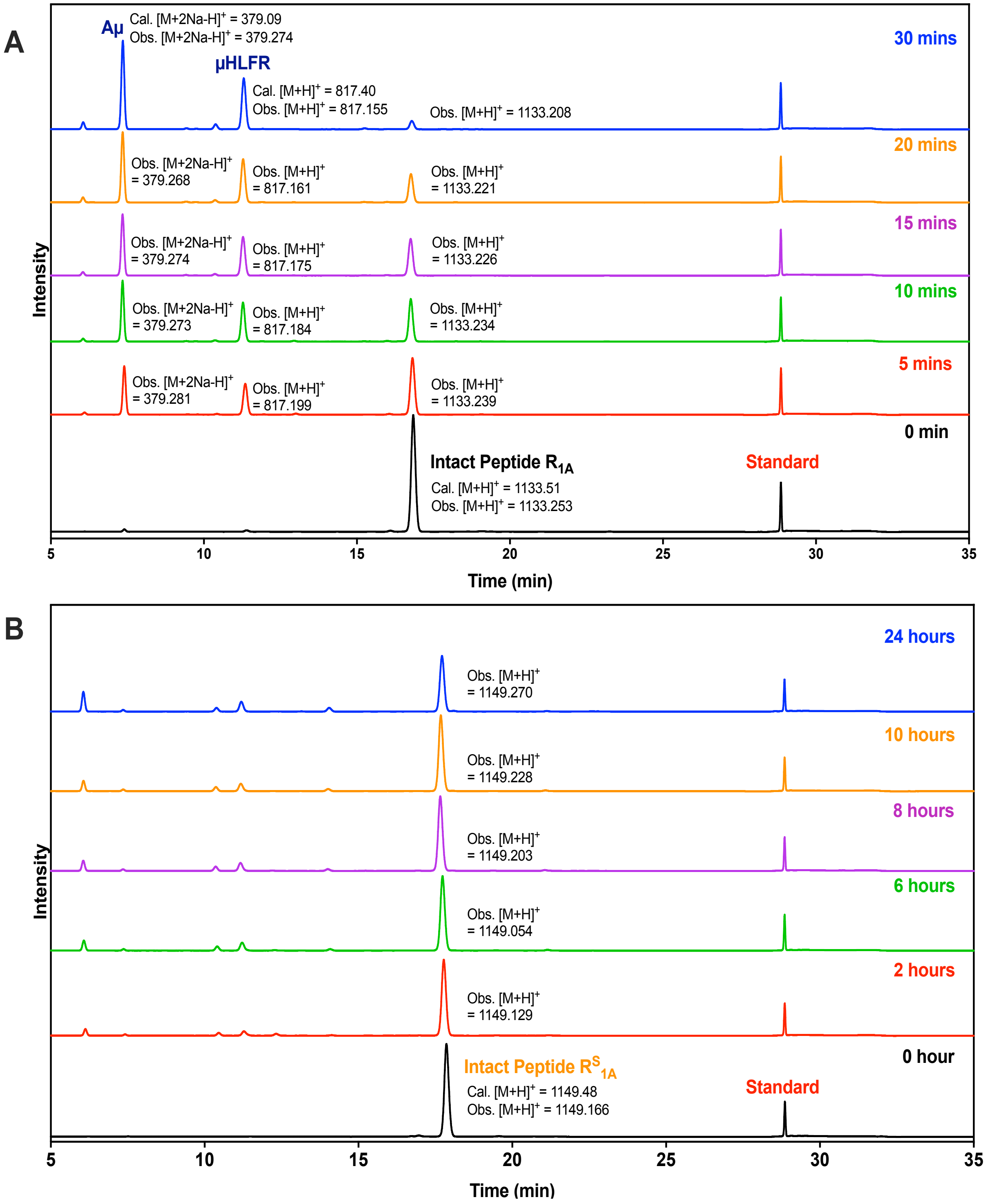

Figure S26. Stability of 20 µM R_{1A} Peptide (µHLFRAµ) and R^{S}_{1A} Peptide (µHLFR^{S}Aµ) in 0.05 mg/mL HepG2 Whole Cell Lysate. Representative HPLC traces of mixtures from (A) R_{1A} and (B) R^{S}_{1A} incubated in HepG2 cell lysate at 37 ºC for different time points. Coumarin was used as an internal standard. Masses of all major fractions were confirmed using MALDI MS.

Computational Modeling

Starting Structures. In order to simulate the complexes from this study (**Figures S27-46**) using the Rosetta FlexPepDock algorithm, the 1BP4 structure of the papain protease which contains a peptide-like covalent inhibitor[8] was used as a template in order to provide a reasonable starting orientation for simulation. Manual docking was performed by replacing the native covalent inhibitor with the WHLFRAAAW peptide which was prepared using pose_from_sequence in PyRosetta.[9] The peptide was oriented to maintain close proximity (~3 Å) of the scissile bond (K/A) to the active site Cys25 residue. The cathepsin proteases of interest were then aligned to the manually docked papain complex using PyMOL.[10] The starting structures that were aligned include Cts L (PDB ID 3HHA),[11] Cts V (PDB ID 1FH0),[12] Cts K (PDB ID 1BGO),[13] Cts S (PDB ID 1MS6),[14] Cts B (PDB ID 1GMY).[15]

FlexPepDock. The manually docked cathepsin protease starting complexes were formally docked by performing the FlexPepDock protocol in Rosetta in order to optimize the binding interaction between the proteases and peptides of interest.[17] The flexible peptide docking protocol optimizes the peptide backbone geometry as well as its rigid-body orientation relative to the protein partner iteratively. Additional "on-the-fly" side-chain optimizations are performed to refine the final docked complex output. In this study, for each of the initial cathepsin-peptide complexes, 100 trajectories of the FlexPepDock refine protocol were performed with an initial pre-pack step. The resultant structures were sorted based on the Rosetta full atom total score (ref2015). The lowest energy FlexPepDock complex for each cathepsin protease was moved into the next stage of docking workflow.

Introduction of Methoxycoumarinyl Alanine. Flexible peptide docking was performed with tryptophan residues at the N- and C-terminal positions rather than the experimental 7-

methoxycoumarinyl alanine amino acid residues because when the protocol was performed with unnatural amino acids, it produced structures with inappropriate bond lengths and angles. We therefore made the assumption that the terminal residues could be docked with tryptophan residues and then to methoxycoumarin residues following the flexible peptide docking. Tryptophan residues were mutated to methoxycoumarin residues using the MutateResidue tool from the PyRosetta toolbox utilizing a params and rotamer library generated in our previous publication.[18]

Complex Relaxation. Following mutation of the tryptophan residues to methoxycoumarin residues, a constrained FastRelax was performed in PyRosetta with the following parameters: the beta_nov16 score function,[16] a MoveMap set for both backbone and sidechain optimization, the dualspace and minimize_bond_angles flags set to False, and the lbfgs_armijo_nonmonotone minimizer. The constrained FastRelax was performed utilizing a custom fold tree and constraints to optimize the system following introduction of the methoxycoumarin amino acids.[17] Constraints were used to maintain proximity of the scissile bond to the active site cysteine residue, which may have been altered during FlexPepDock.

Thioamide Modeling and Local Relaxation. Thioamides were introduced into the relaxed complexes through patches written in our previous publication.[17] The thioamide containing peptides were simulated with five independent local relax trajectories and the following parameters: the beta_nov16 score function, a MoveMap set for both backbone and sidechain optimization of only residues that have a C-alpha atom within 8 Å of the C-alpha of the residue containing the thioamide, dualspace and minimize_bond_angles set to False, the lbfgs_armijo_nonmonotone minimizer, and the same foldtree as used previously, but without any constraints.

Table S25. Distances from the catalytic cysteine of the proteases to the P1 (Arg) carbonyl of R_{3A}, R^{S}_{3A}, R_{1A}, and R^{S}_{1A}.

**Peptide**	**Cts L Cys25 – C=O/S**	**Cts V Cys25 – C=O/S**	**Cts K Cys25 – C=O/S**	**Cts S Cys25 – C=O/S**	**Cts B Cys30 – C=O/S**
R_{3A}	3.5	3.5	3.5	3.5	3.5
R^{S}_{3A}	4.0	4.0	3.7	3.4	3.8
R_{1A}	3.8	3.8	3.7	3.3	3.6
R^{S}_{1A}	5.0	4.1	3.9	4.3	3.7

* All distances are reported in Å.

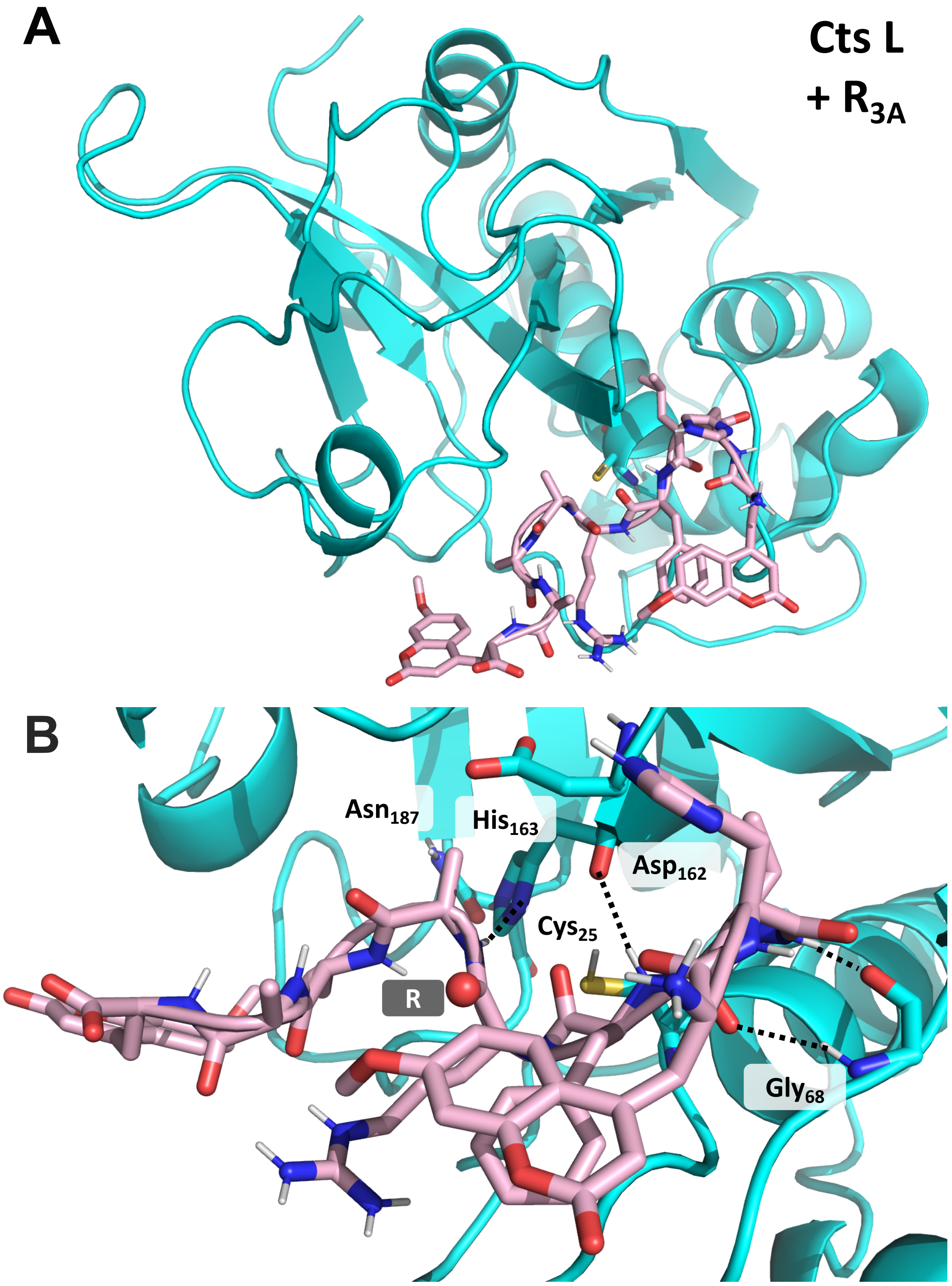

Figure S27. Structural Modeling of Cts L (cyan) with R_{3A} peptide (μHLFRAAAμ; light pink). (A) Cts L in complex with R_{3A} peptide. (B) Key interactions of R_{3A} peptide at the active site of Cts L. The catalytic triad of Cts L are shown: Cys_{25}, His_{163}, and Asn_{187}. The Arg carbonyl of R_{3A} peptide is shown as a red sphere. Key interactions are shown as black dashed lines.

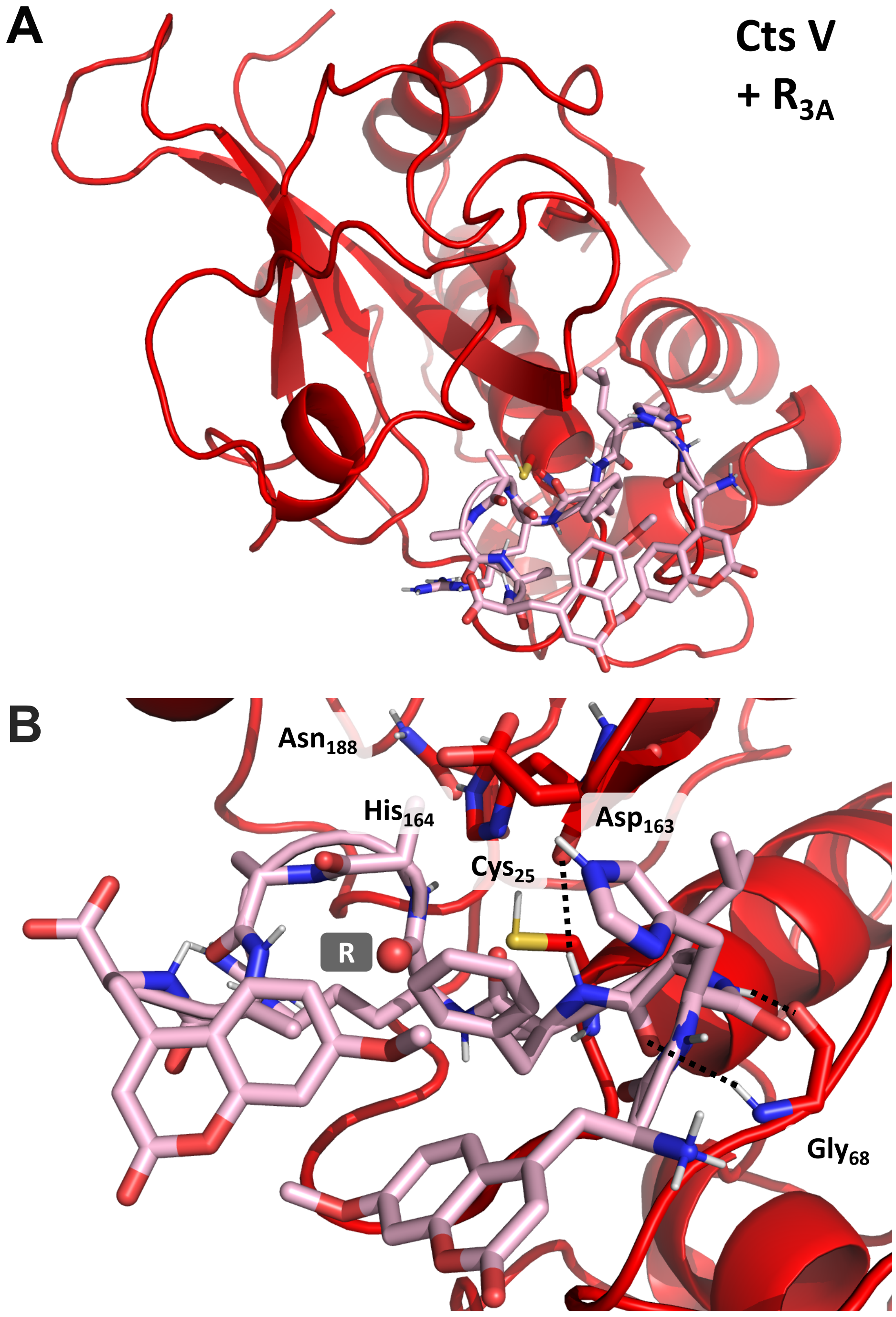

Figure S28. Structural Modeling of Cts V (red) with R_{3A} peptide (μHLFRAAAμ; light pink). (A) Cts V in complex with R_{3A} peptide. (B) Key interactions of R_{3A} peptide at the active site of Cts V. The catalytic triad of Cts V are shown: Cys_{25}, His_{164}, and Asn_{188}. The Arg carbonyl of R_{3A} peptide is shown as a red sphere. Key interactions are shown as black dashed lines.

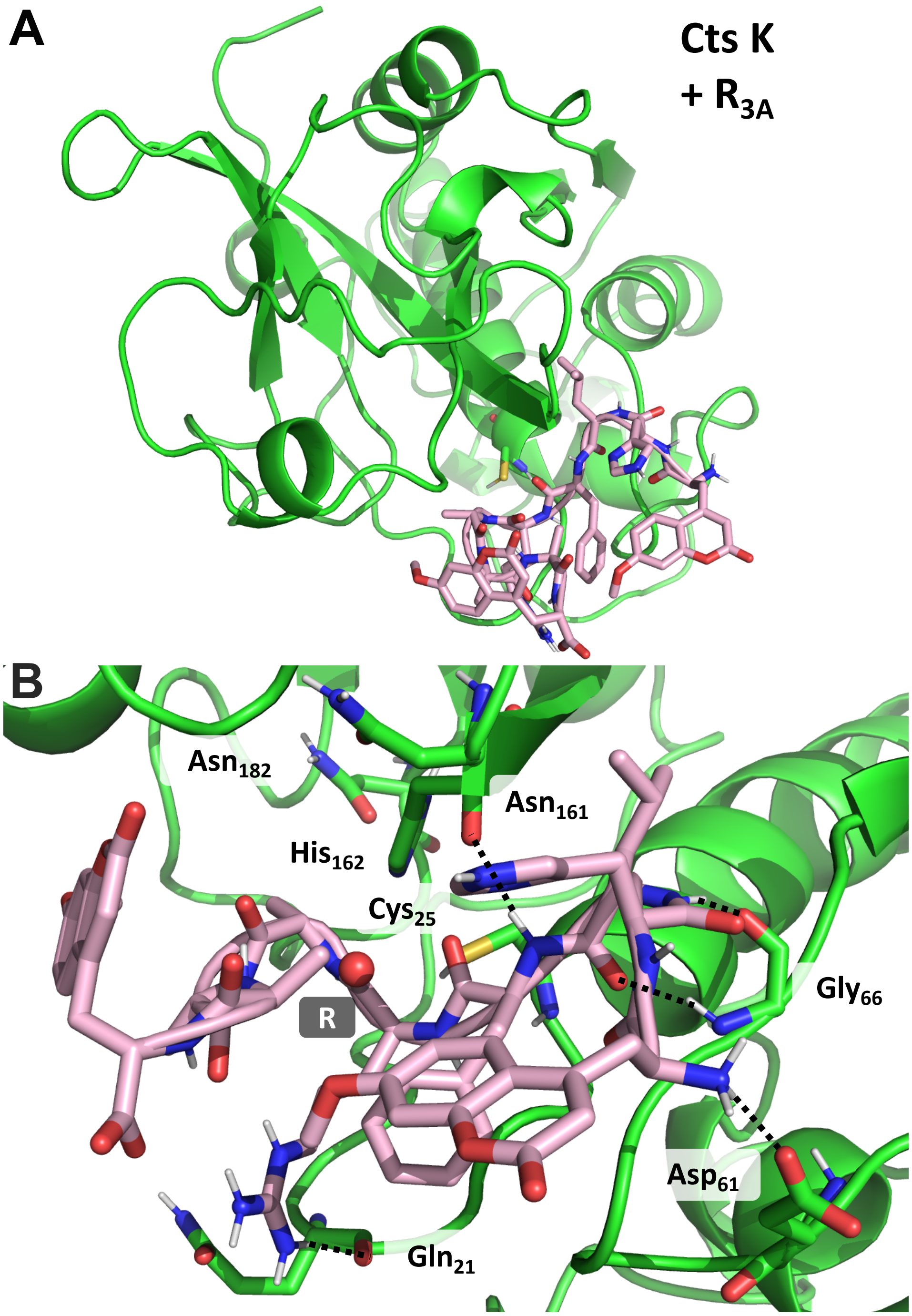

Figure S29. Structural Modeling of Cts K (green) with R_{3A} peptide (μHLFRAAAμ; light pink). (A) Cts K in complex with R_{3A} peptide. (B) Key interactions of R_{3A} peptide at the active site of Cts K. The catalytic triad of Cts K are shown: Cys_{25}, His_{162}, and Asn_{182}. The Arg carbonyl of R_{3A} peptide is shown as a red sphere. Key interactions are shown as black dashed lines.

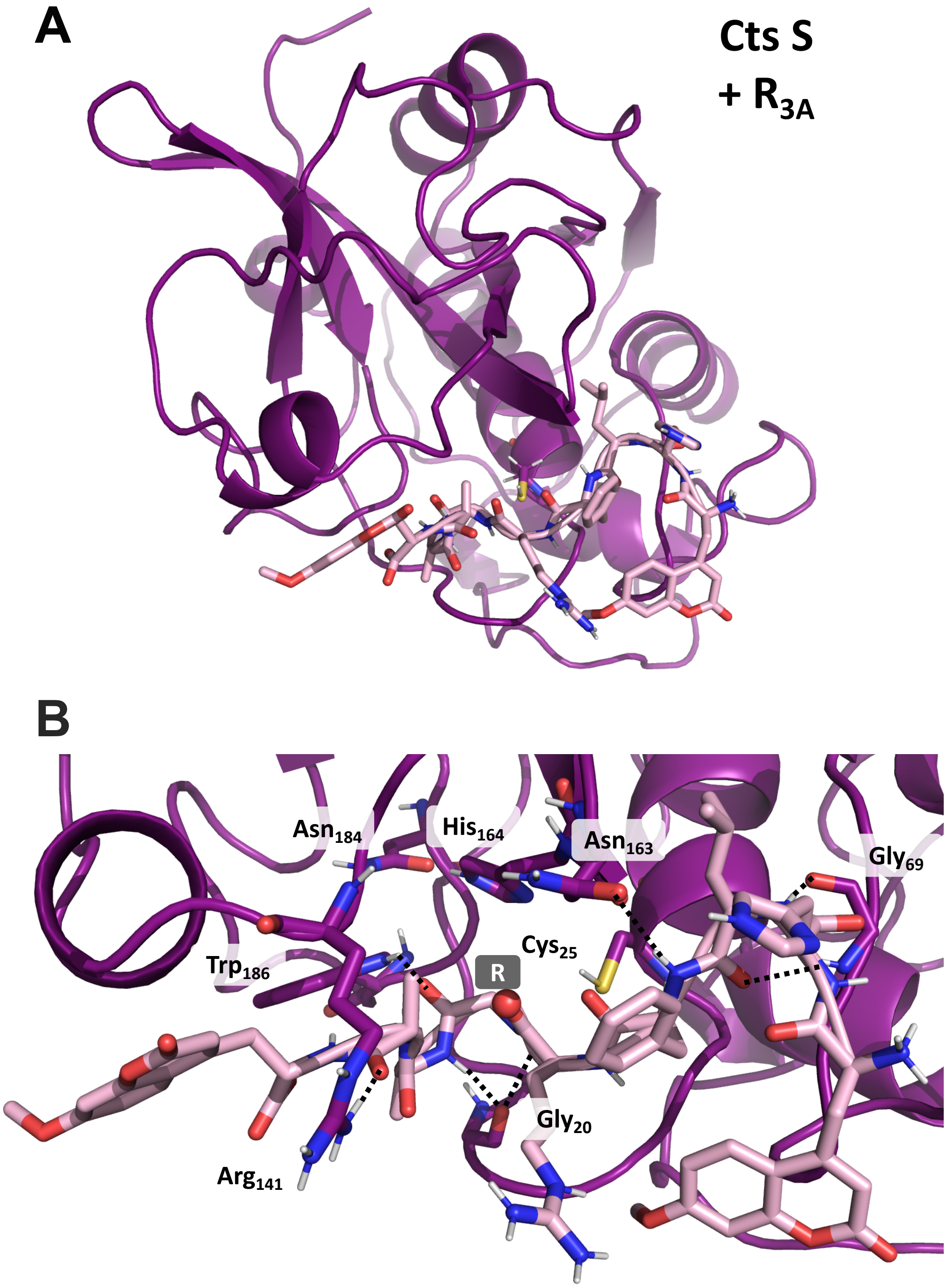

Figure S30. Structural Modeling of Cts S (deep purple) with R_{3A} peptide (µHLFRAAAµ; light pink). (A) Cts S in complex with R_{3A} peptide. (B) Key interactions of R_{3A} peptide at the active site of Cts S. The catalytic triad of Cts S are shown: Cys_{25}, His_{164}, and Asn_{184}. The Arg carbonyl of R_{3A} peptide is shown as a red sphere. Key interactions are shown as black dashed lines.

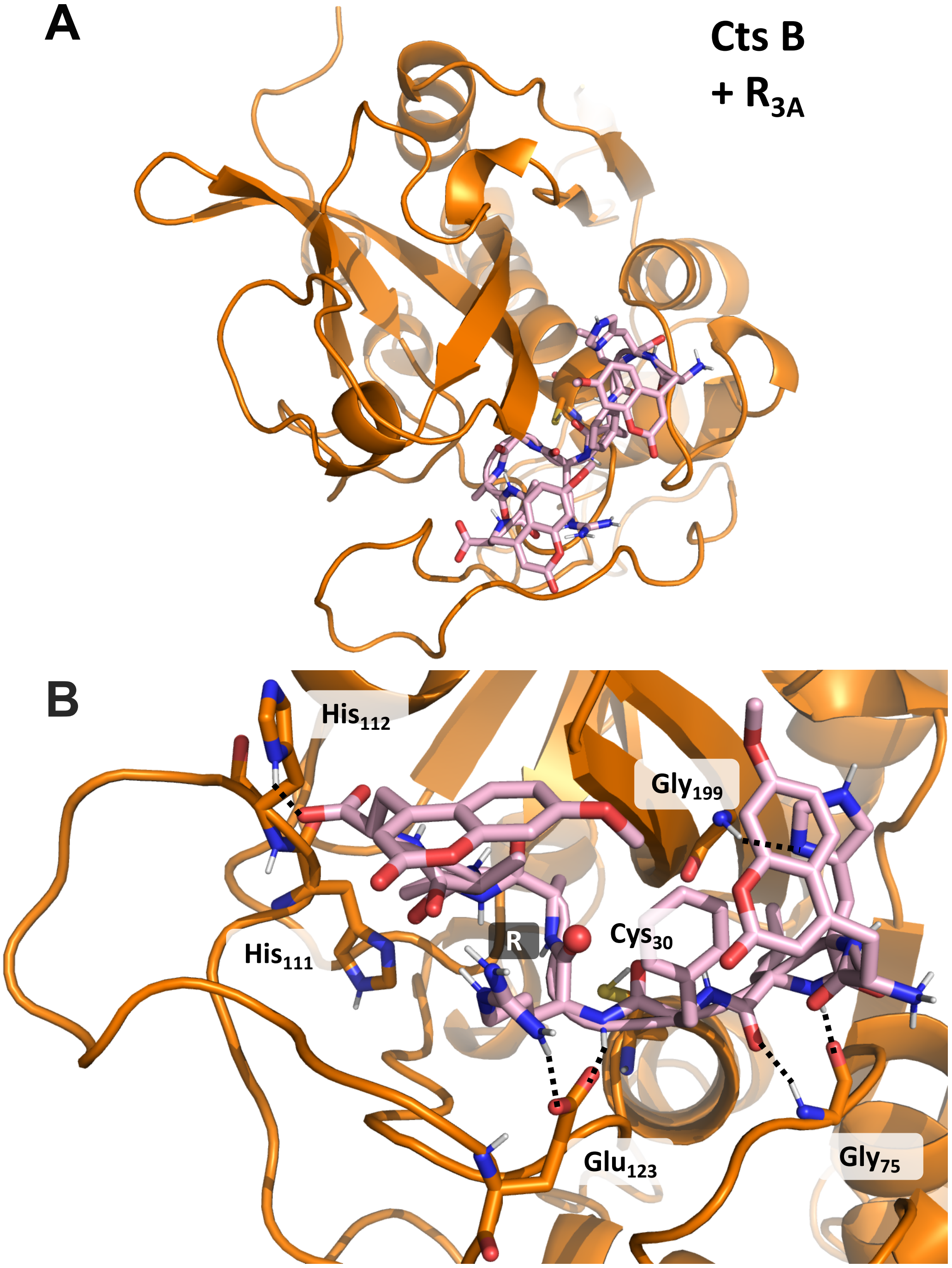

Figure S31. Structural Modeling of Cts B (orange) with R_{3A} peptide (μHLFRAAAμ; light pink). (A) Cts B in complex with R_{3A} peptide. (B) Key interactions of R_{3A} peptide at the active site of Cts B. The catalytic Cys_{30} of Cts B is shown along with His_{111} and His_{112} on the occluding loop – the two key His residues for Cts B's carboxydipeptidase property. The Arg carbonyl of R_{3A} peptide is shown as a red sphere. Key interactions are shown as black dashed lines.

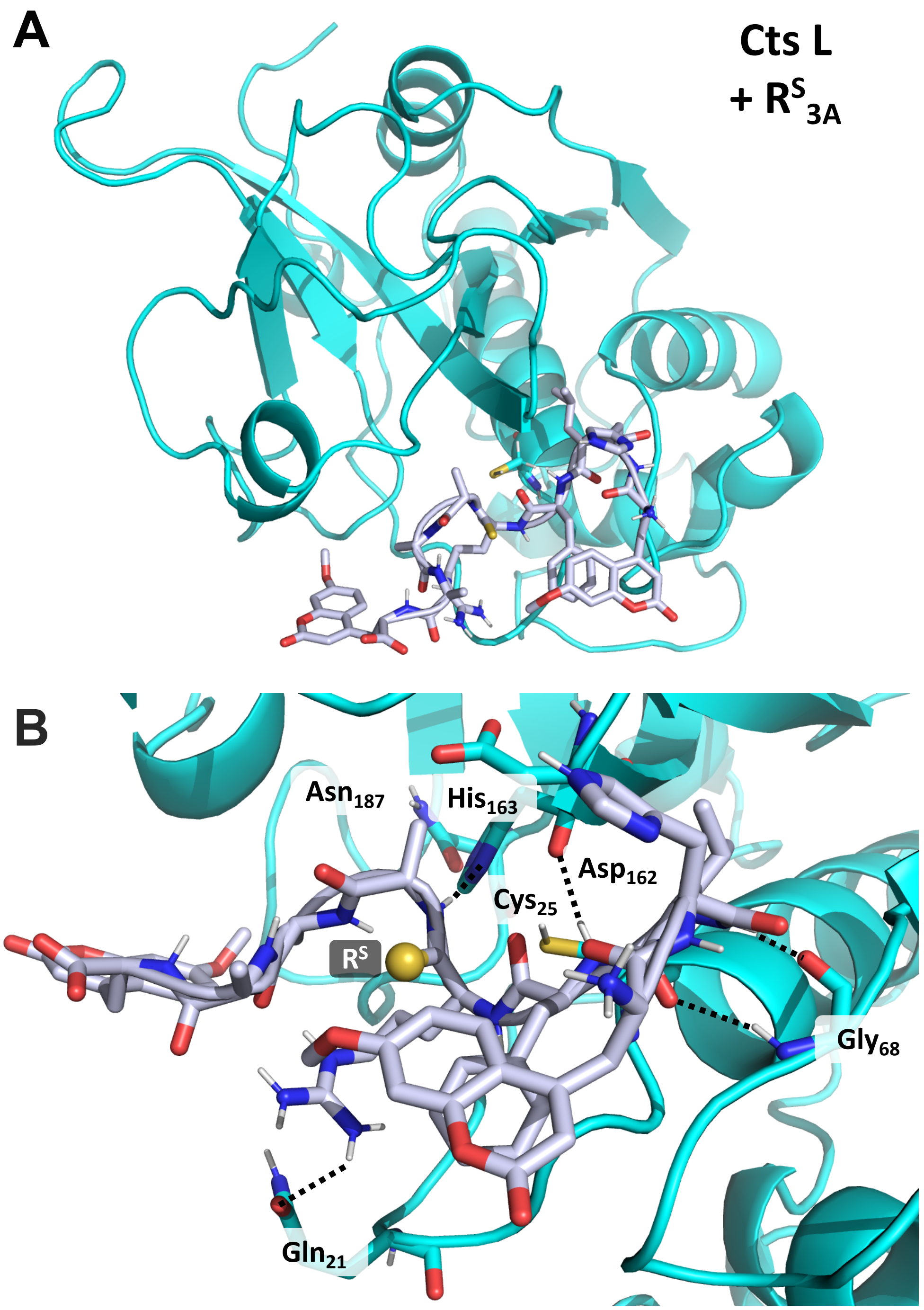

Figure S32. Structural Modeling of Cts L (cyan) with R^S_{3A} peptide (μHLFR^{S}AAAμ; blue white). (A) Cts L in complex with R^S_{3A} peptide. (B) Key interactions of R^S_{3A} peptide at the active site of Cts L. The catalytic triad of Cts L are shown: Cys_{25}, His_{163}, and Asn_{187}. The thioamide carbonyl of R^S_{3A} peptide is shown as a yellow sphere. Key interactions are shown as black dashed lines.

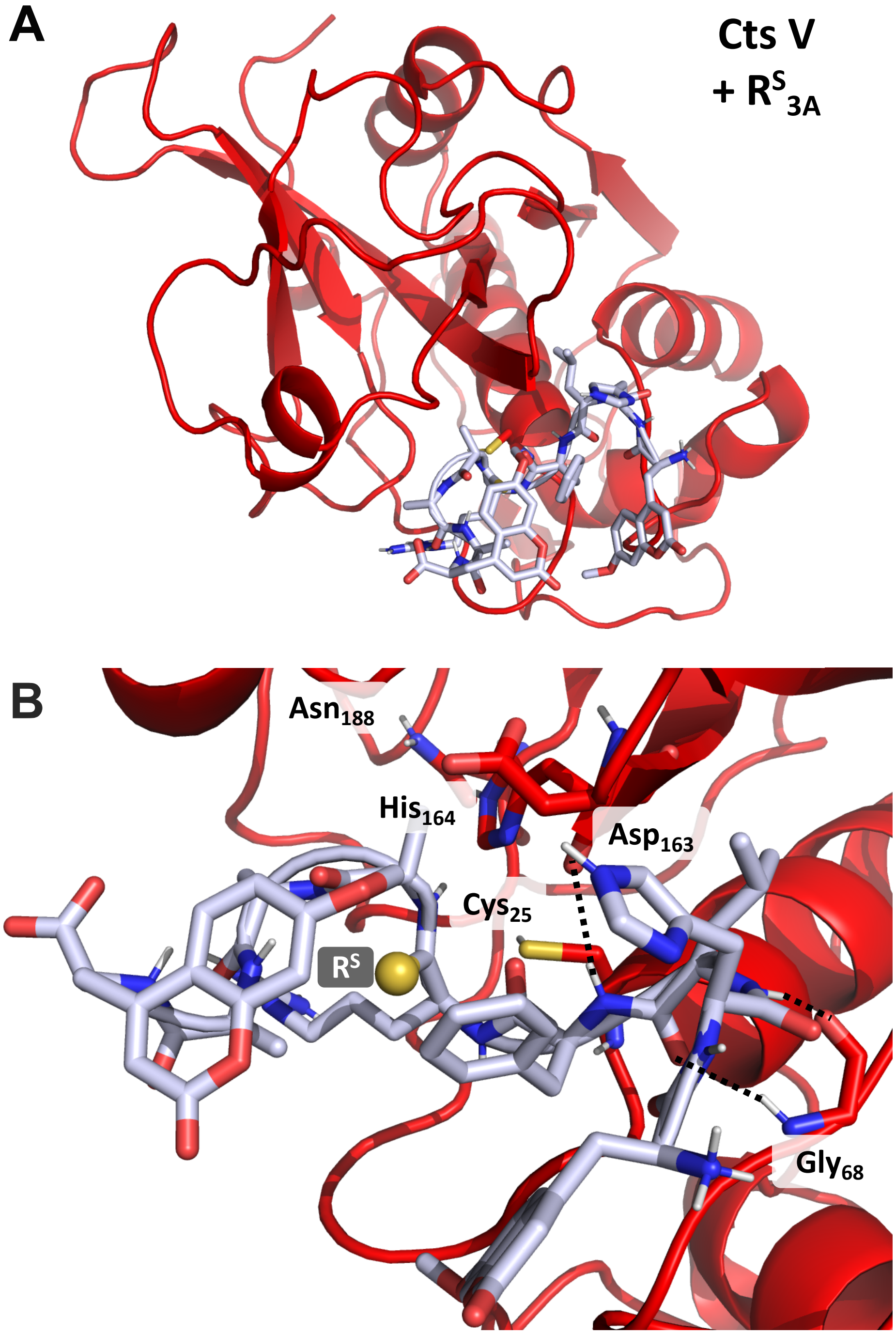

Figure S33. Structural Modeling of Cts V (red) with R^S_{3A} peptide (μHLFR^{S}AAAμ; blue white). (A) Cts V in complex with R^S_{3A} peptide. (B) Key interactions of R^S_{3A} peptide at the active site of Cts V. The catalytic triad of Cts V are shown: Cys_{25}, His_{164}, and Asn_{188}. The thioamide carbonyl of R^S_{3A} peptide is shown as a yellow sphere. Key interactions are shown as black dashed lines.

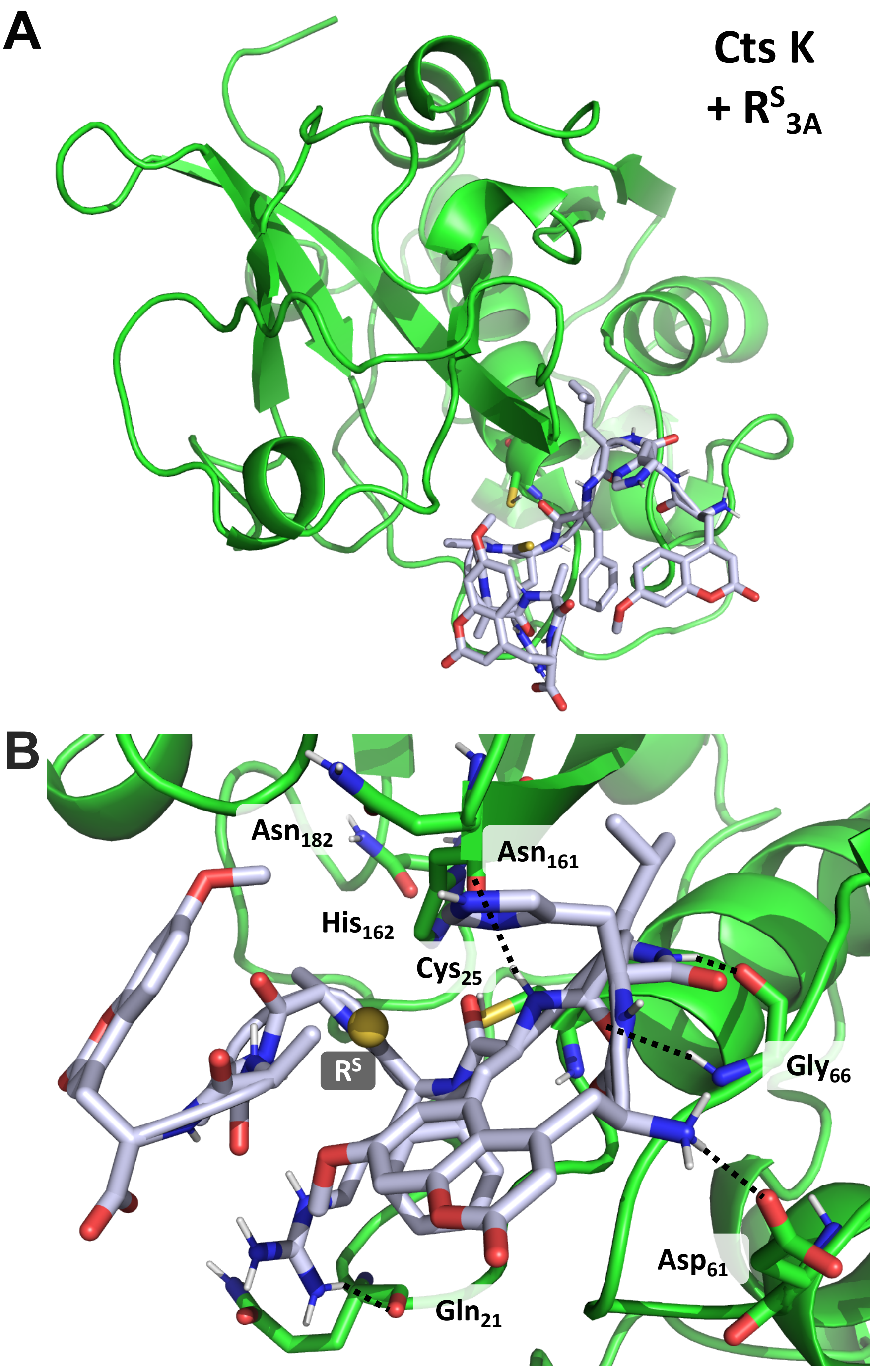

Figure S34. Structural Modeling of Cts K (green) with R^{S}_{3A} peptide (μHLFR^{S}AAAμ; blue white). (A) Cts K in complex with R^{S}_{3A} peptide. (B) Key interactions of R^{S}_{3A} peptide at the active site of Cts K. The catalytic triad of Cts K are shown: Cys_{25}, His_{162}, and Asn_{182}. The thioamide carbonyl of R^{S}_{3A} peptide is shown as a yellow sphere. Key interactions are shown as black dashed lines.

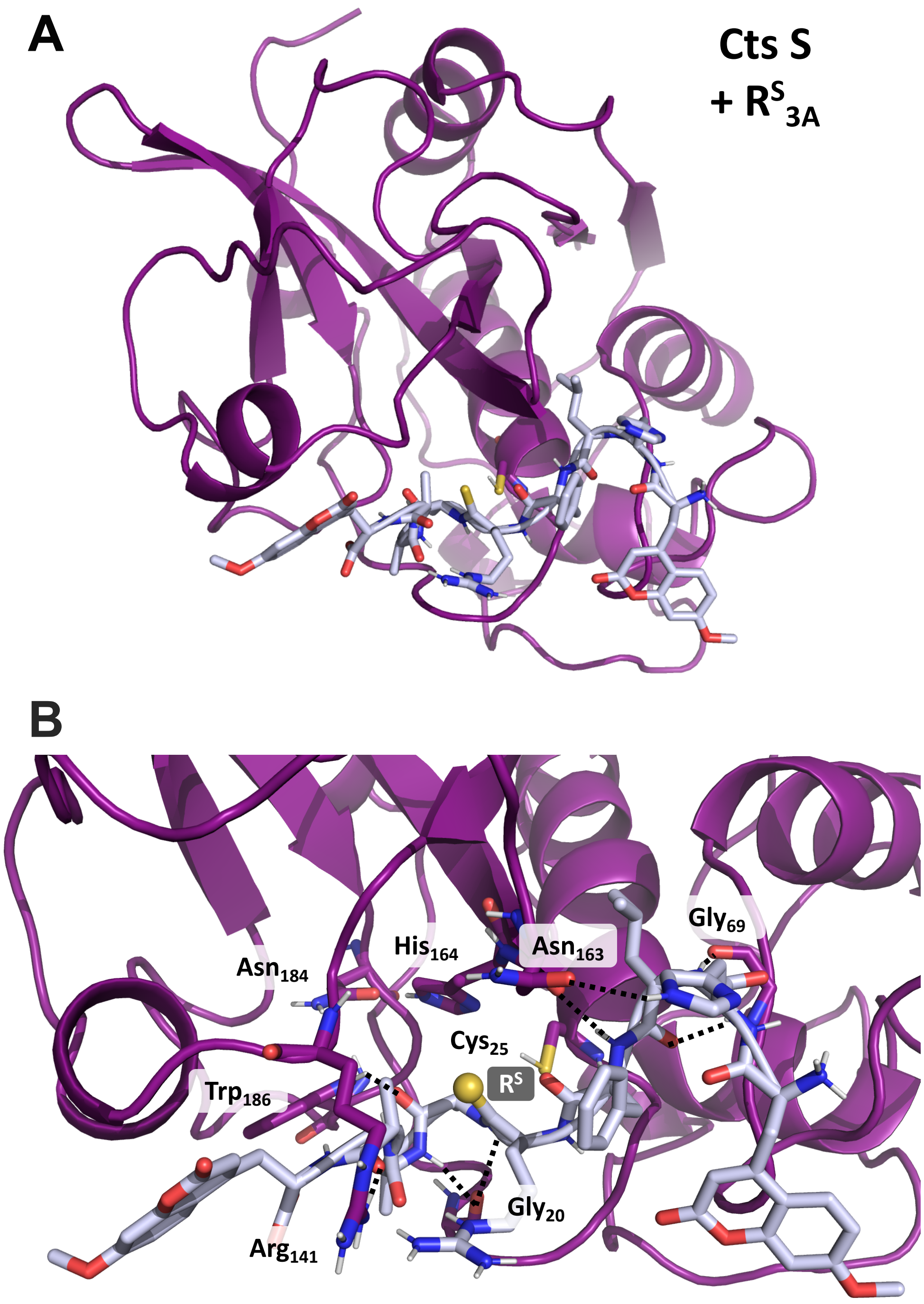

Figure S35. Structural Modeling of Cts S (deep purple) with R$^{S}_{3A}$ peptide (μHLFR^{S}AAAμ; blue white). (A) Cts S in complex with R$^{S}_{3A}$ peptide. (B) Key interactions of R$^{S}_{3A}$ peptide at the active site of Cts S. The catalytic triad of Cts S are shown: Cys$_{25}$, His$_{164}$, and Asn$_{184}$. The thioamide carbonyl of R$^{S}_{3A}$ peptide is shown as a yellow sphere. Key interactions are shown as black dashed lines.

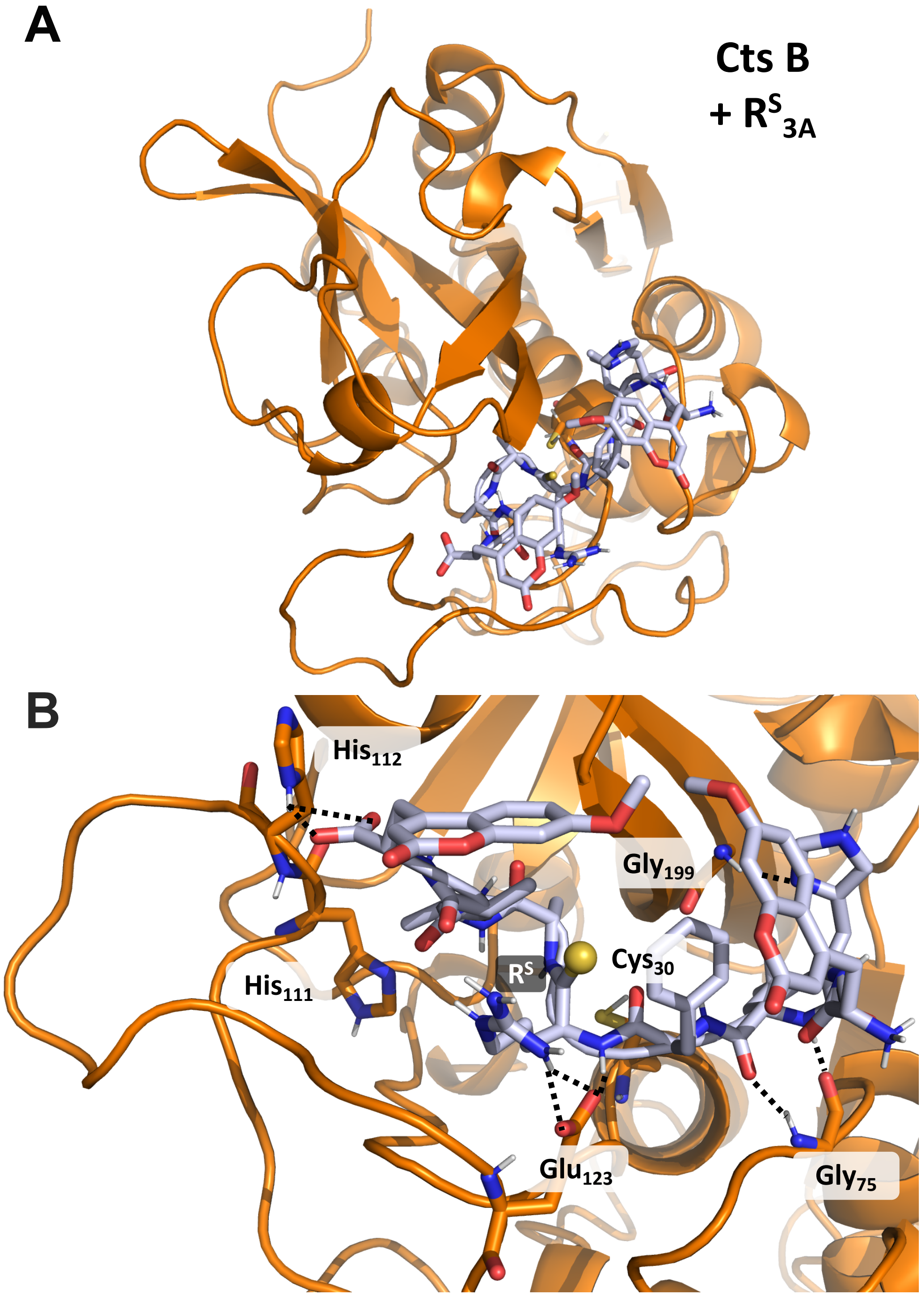

Figure S36. Structural Modeling of Cts B (orange) with R^{S}_{3A} peptide (μHLFR^{S}AAAμ; blue white). (A) Cts B in complex with R^{S}_{3A} peptide. (B) Key interactions of R^{S}_{3A} peptide at the active site of Cts B. The catalytic Cys_{30} of Cts B is shown along with His_{111} and His_{112} on the occluding loop – the two key His residues for Cts B's carboxydipeptidase property. The thioamide carbonyl of R^{S}_{3A} peptide is shown as a yellow sphere. Key interactions are shown as black dashed lines.

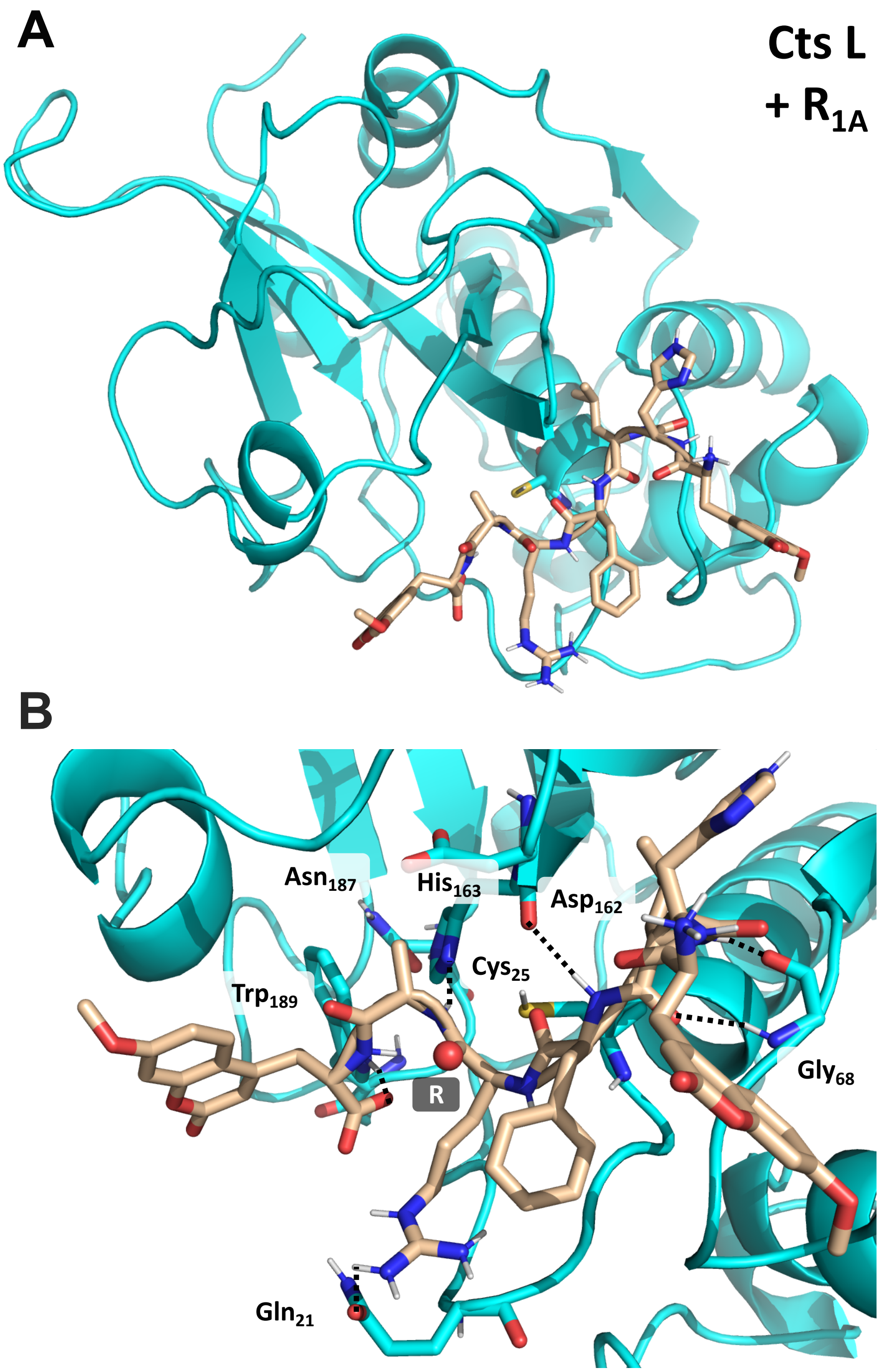

Figure S37. Structural Modeling of Cts L (cyan) with R_{1A} peptide (μHLFRAμ; wheat). (A) Cts L in complex with R_{1A} peptide. (B) Key interactions of R_{1A} peptide at the active site of Cts L. The catalytic triad of Cts L are shown: Cys_{25}, His_{163}, and Asn_{187}. The Arg carbonyl of R_{1A} peptide is shown as a red sphere. Key interactions are shown as black dashed lines.

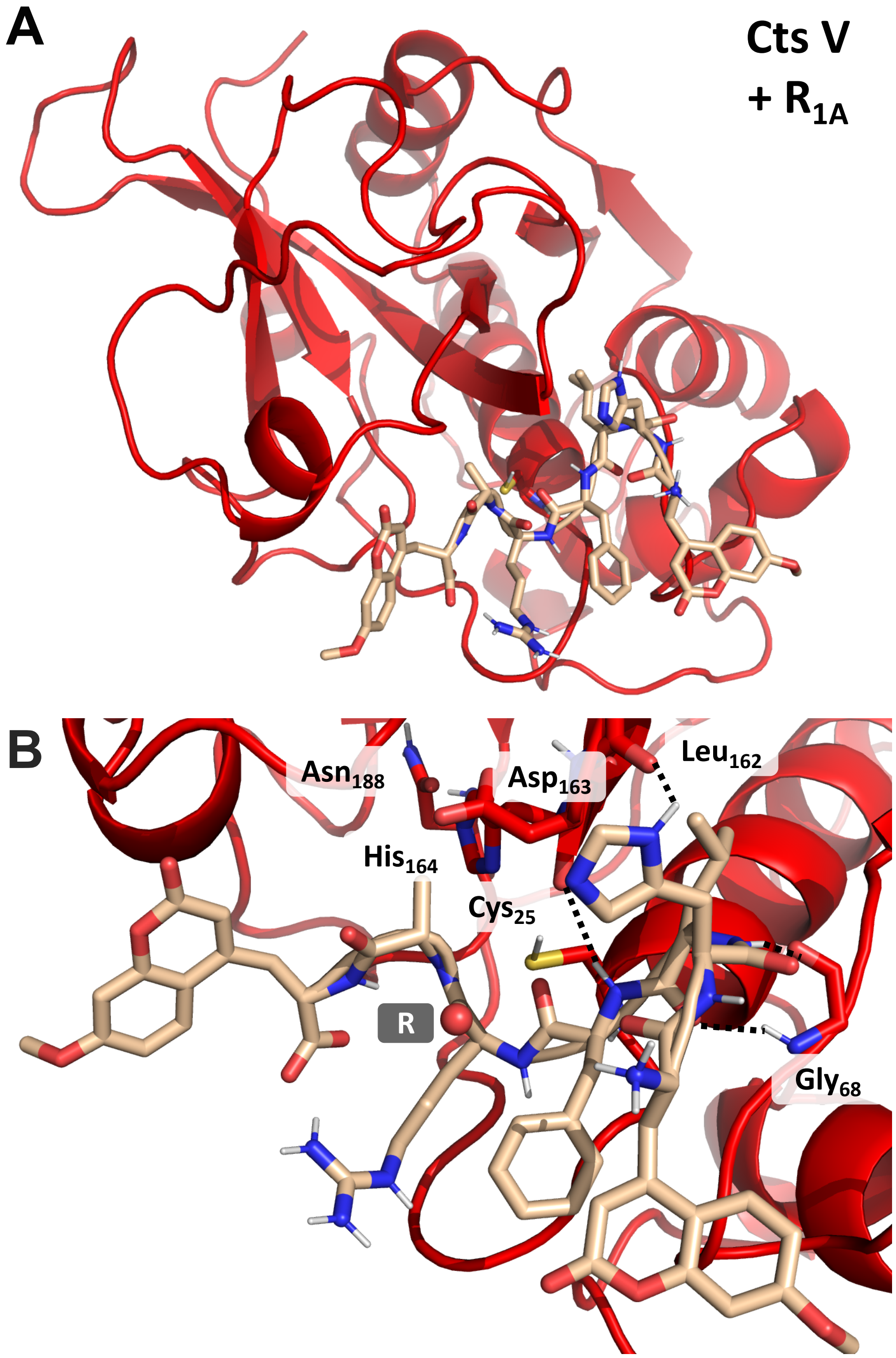

Figure S38. Structural Modeling of Cts V (red) with R_{1A} peptide (µHLFRAµ; wheat). (A) Cts V in complex with R_{1A} peptide. (B) Key interactions of R_{1A} peptide at the active site of Cts V. The catalytic triad of Cts V are shown: Cys_{25}, His_{164}, and Asn_{188}. The Arg carbonyl of R_{1A} peptide is shown as a red sphere. Key interactions are shown as black dashed lines.

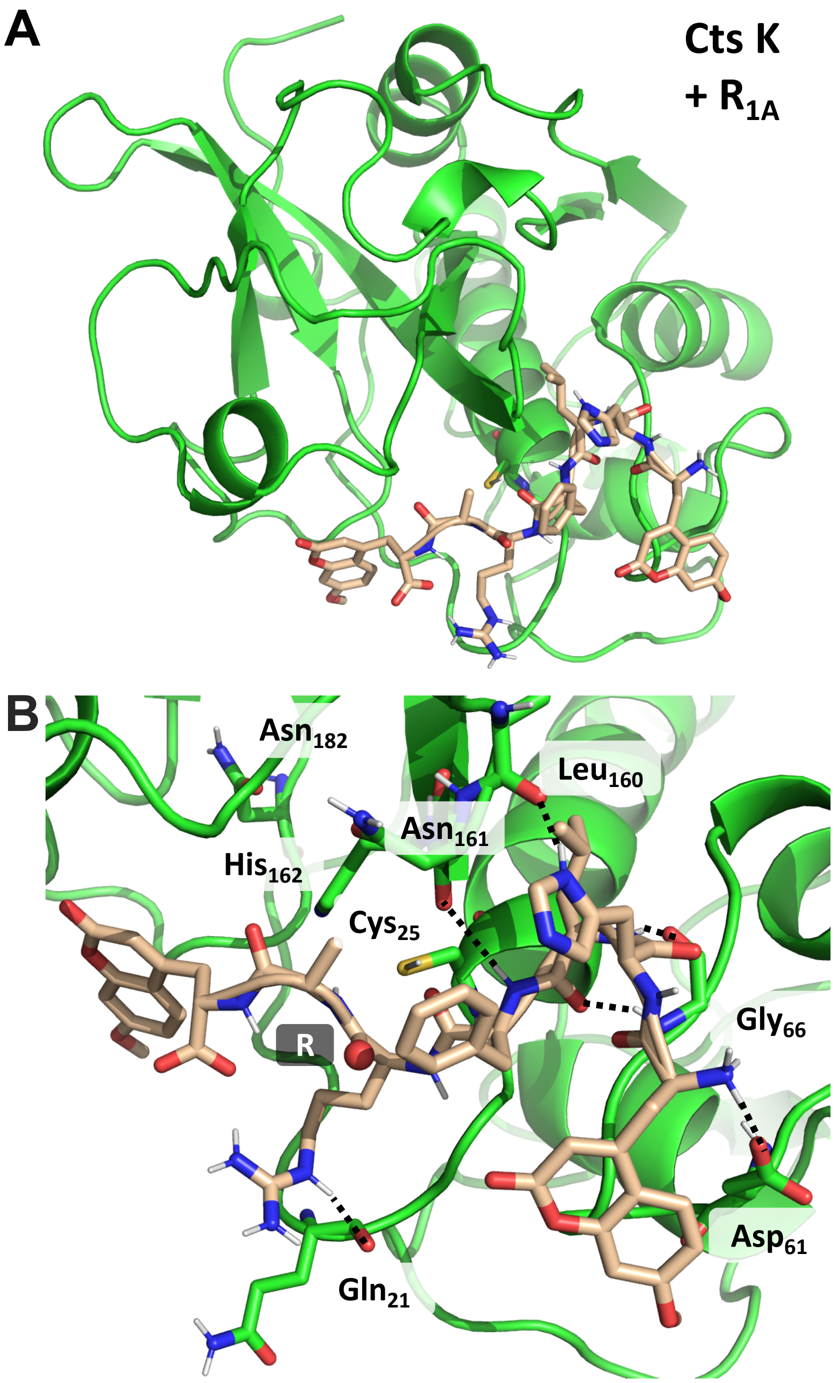

Figure S39. Structural Modeling of Cts K (green) with R_{1A} peptide (μHLFRAμ; wheat). (A) Cts K in complex with R_{1A} peptide. (B) Key interactions of R_{1A} peptide at the active site of Cts K. The catalytic triad of Cts K are shown: Cys_{25}, His_{162}, and Asn_{182}. The Arg carbonyl of R_{1A} peptide is shown as a red sphere. Key interactions are shown as black dashed lines.

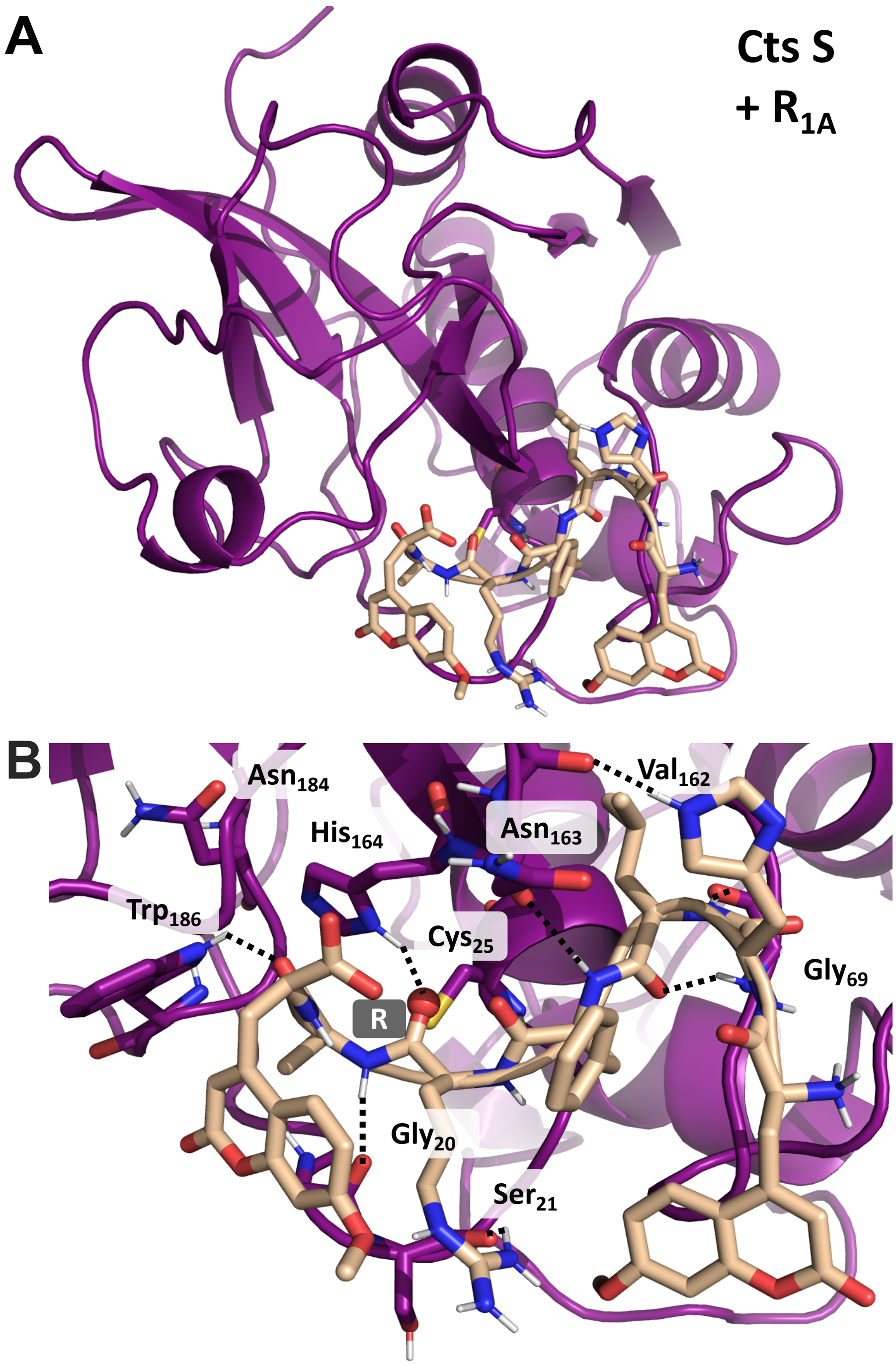

Figure S40. Structural Modeling of Cts S (deep purple) with R_{1A} peptide (µHLFRAµ; wheat). (A) Cts S in complex with R_{1A} peptide. (B) Key interactions of R_{1A} peptide at the active site of Cts S. The catalytic triad of Cts S are shown: Cys_{25}, His_{164}, and Asn_{184}. The Arg carbonyl of R_{1A} peptide is shown as a red sphere. Key interactions are shown as black dashed lines.

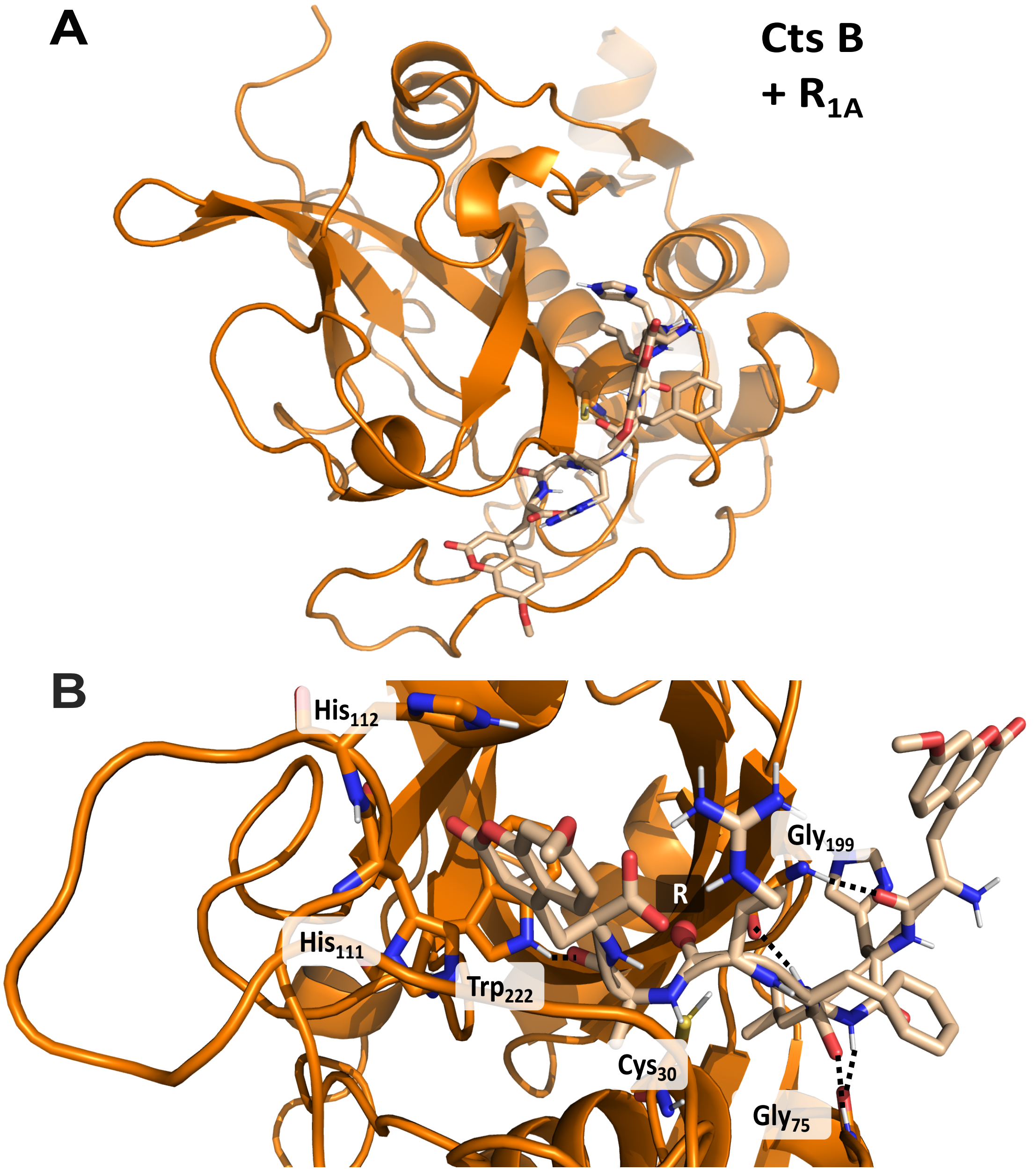

Figure S41. Structural Modeling of Cts B (orange) with R_{1A} peptide (μHLFRAμ; wheat). (A) Cts B in complex with R_{1A} peptide. (B) Key interactions of R_{1A} peptide at the active site of Cts B. The catalytic Cys_{30} of Cts B is shown along with His_{111} and His_{112} on the occluding loop – the two key His residues for Cts B's carboxydipeptidase property. The Arg carbonyl of R_{1A} peptide is shown as a red sphere. Key interactions are shown as black dashed lines.

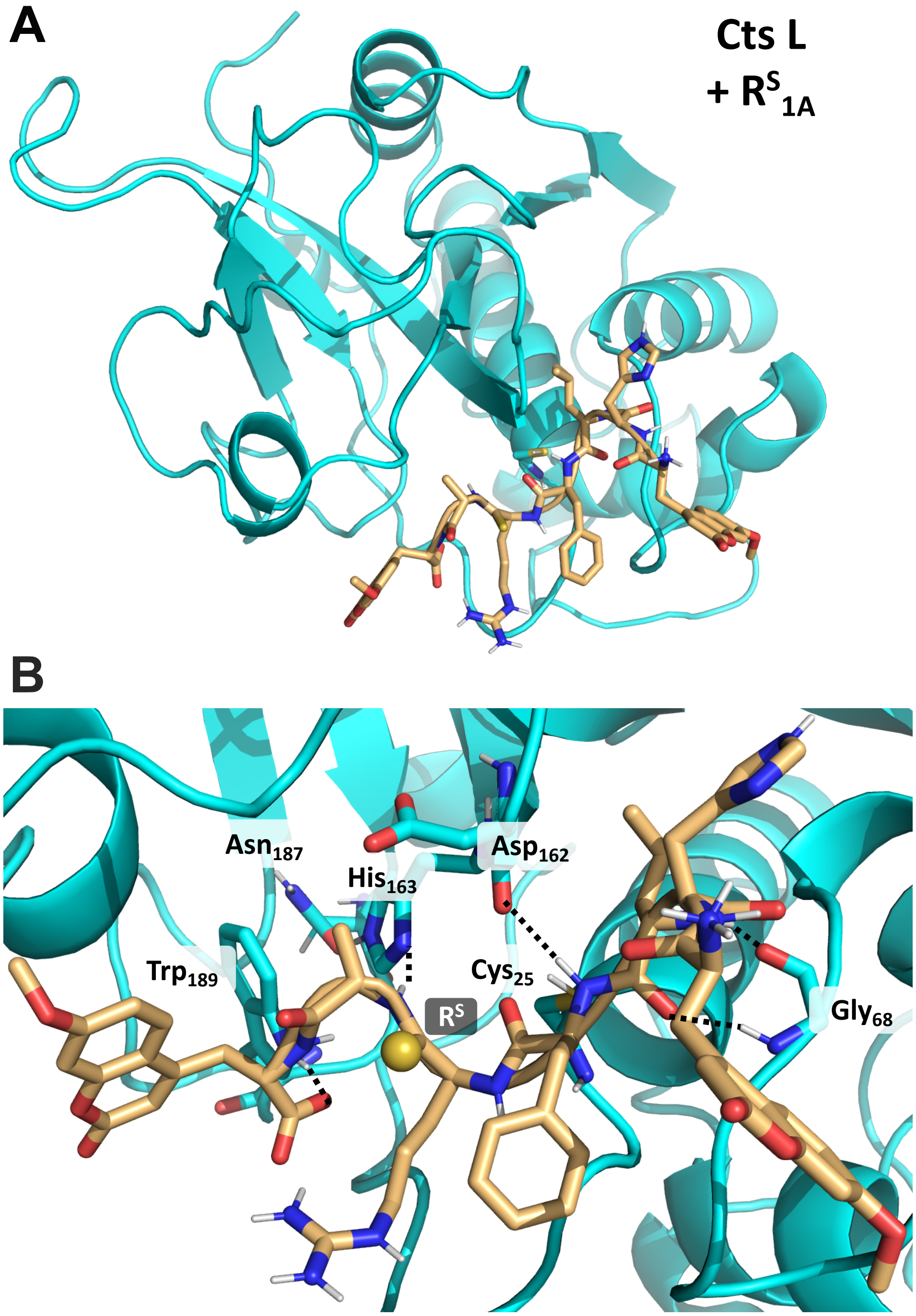

Figure S42. Structural Modeling of Cts L (cyan) with R$^{S}{}_{1A}$ peptide (μHLFR^{S}Aμ; light orange). (A) Cts L in complex with R$^{S}{}_{1A}$ peptide. (B) Key interactions of R$^{S}{}_{1A}$ peptide at the active site of Cts L. The catalytic triad of Cts L are shown: Cys$_{25}$, His$_{163}$, and Asn$_{187}$. The thioamide carbonyl of R$^{S}{}_{1A}$ peptide is shown as a yellow sphere. Key interactions are shown as black dashed lines.

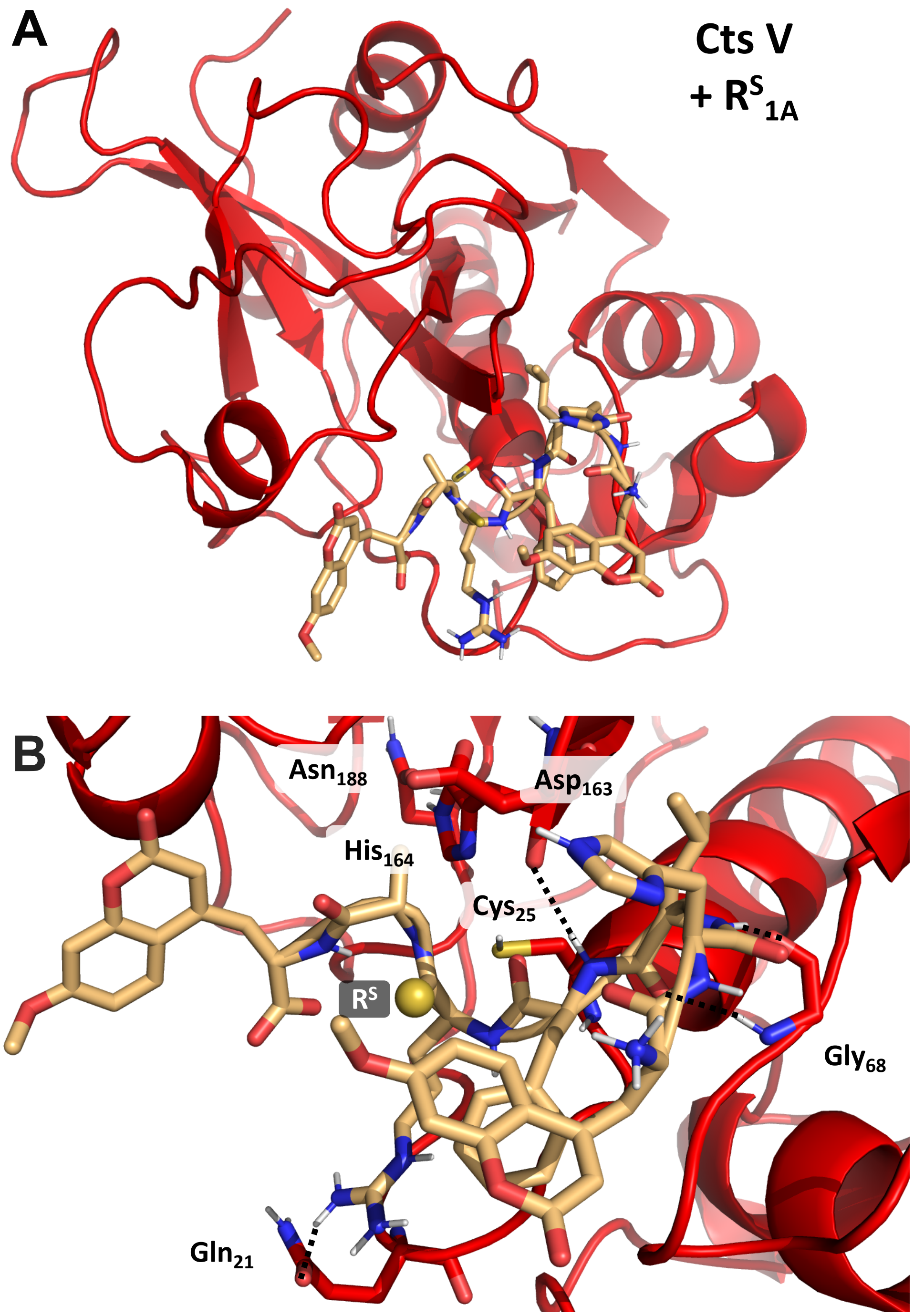

Figure S43. Structural Modeling of Cts V (red) with R^S_{1A} peptide (μHLFR^{S}Aμ; light orange). (A) Cts V in complex with R^S_{1A} peptide. (B) Key interactions of R^S_{1A} peptide at the active site of Cts V. The catalytic triad of Cts V are shown: Cys_{25}, His_{164}, and Asn_{188}. The thioamide carbonyl of R^S_{1A} peptide is shown as a yellow sphere. Key interactions are shown as black dashed lines.

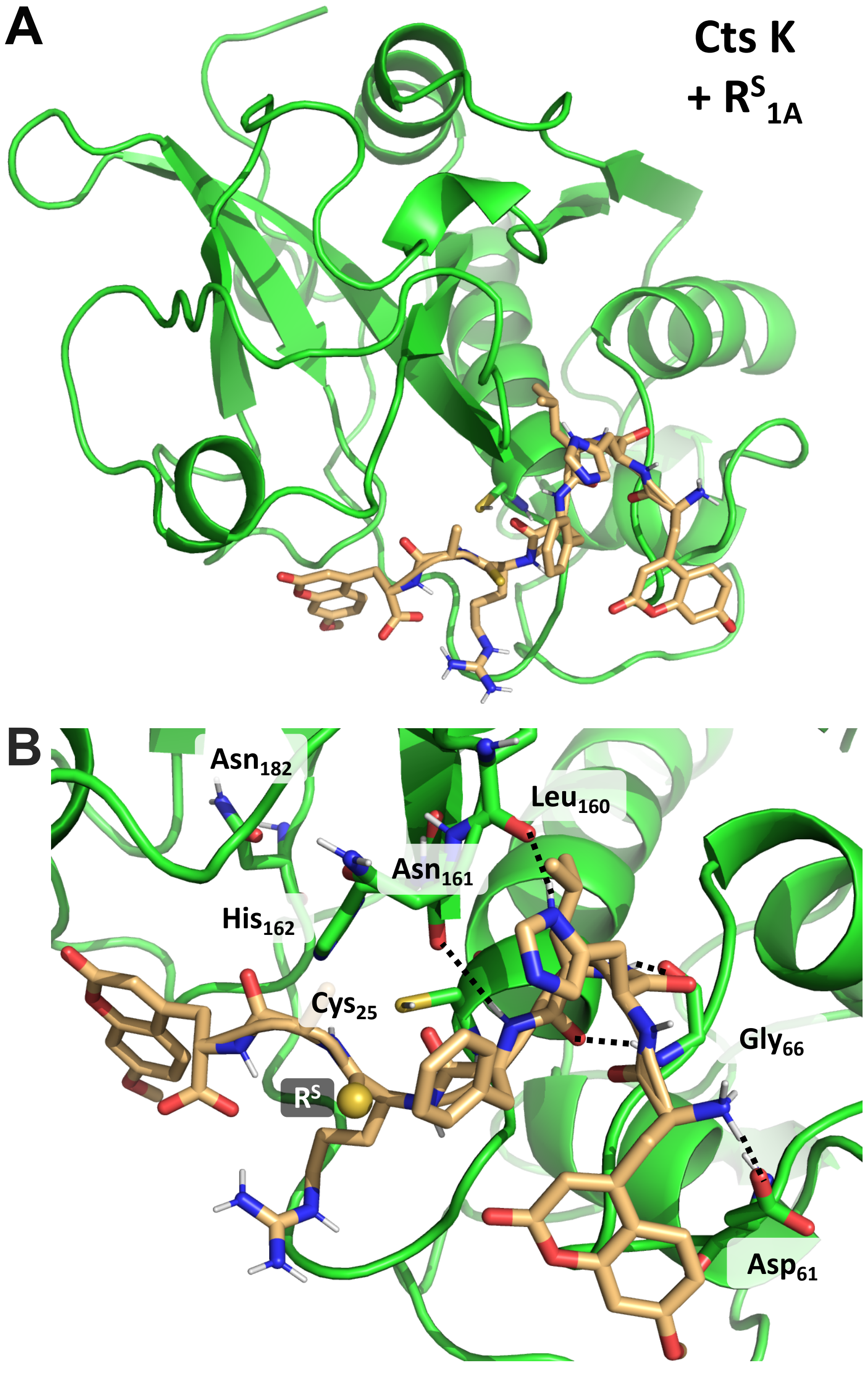

Figure S44. Structural Modeling of Cts K (green) with R^{S}_{1A} peptide (μHLFR^{S}Aμ; light orange). (A) Cts K in complex with R^{S}_{1A} peptide. (B) Key interactions of R^{S}_{1A} peptide at the active site of Cts K. The catalytic triad of Cts K are shown: Cys_{25}, His_{162}, and Asn_{182}. The thioamide carbonyl of R^{S}_{1A} peptide is shown as a yellow sphere. Key interactions are shown as black dashed lines.

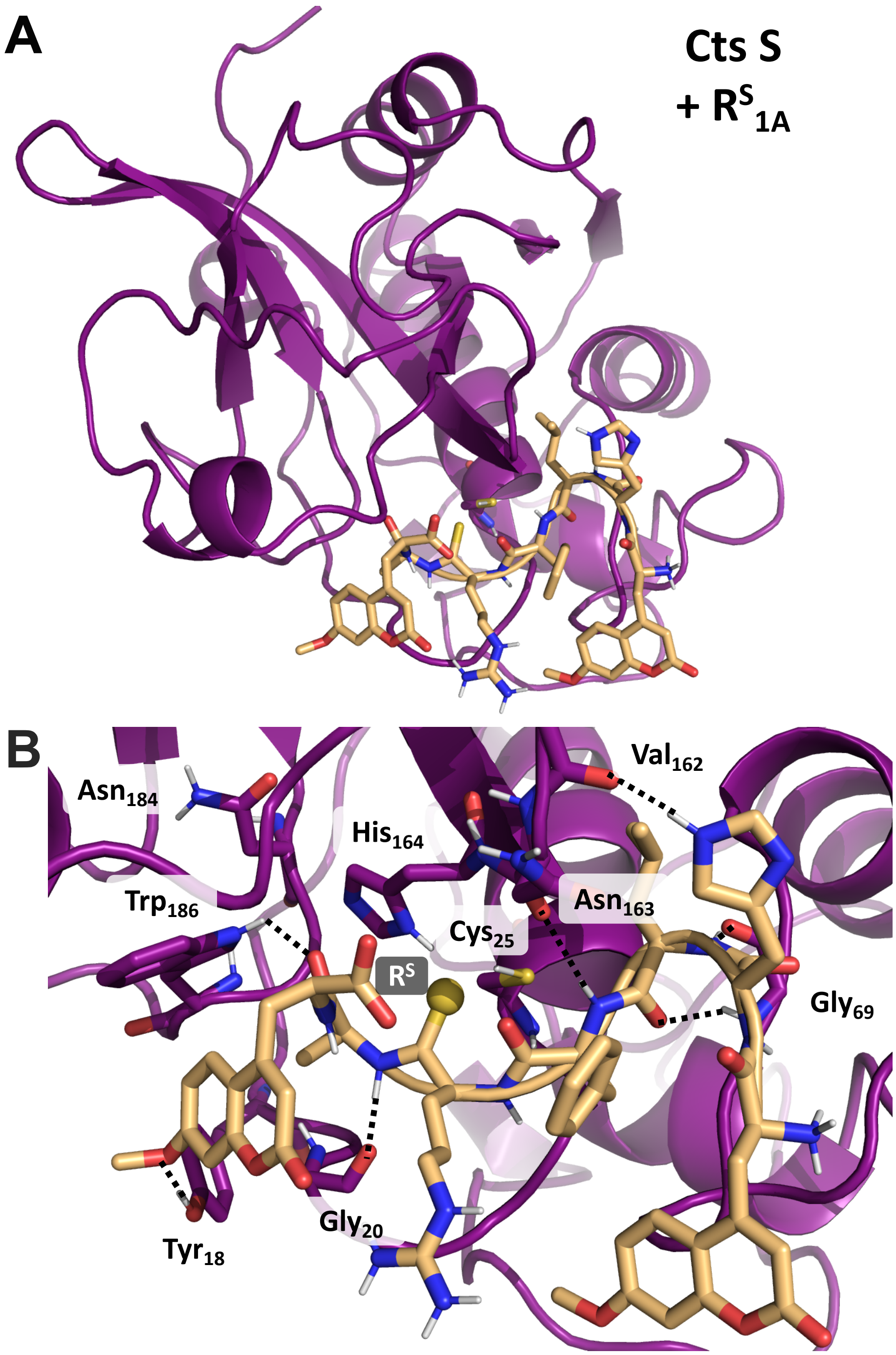

Figure S45. Structural Modeling of Cts S (deep purple) with R$^S_{1A}$ peptide (μHLFR^{S}Aμ; light orange). (A) Cts S in complex with R$^S_{1A}$ peptide. (B) Key interactions of R$^S_{1A}$ peptide at the active site of Cts S. The catalytic triad of Cts S are shown: Cys$_{25}$, His$_{164}$, and Asn$_{184}$. The thioamide carbonyl of R$^S_{1A}$ peptide is shown as a yellow sphere. Key interactions are shown as black dashed lines.

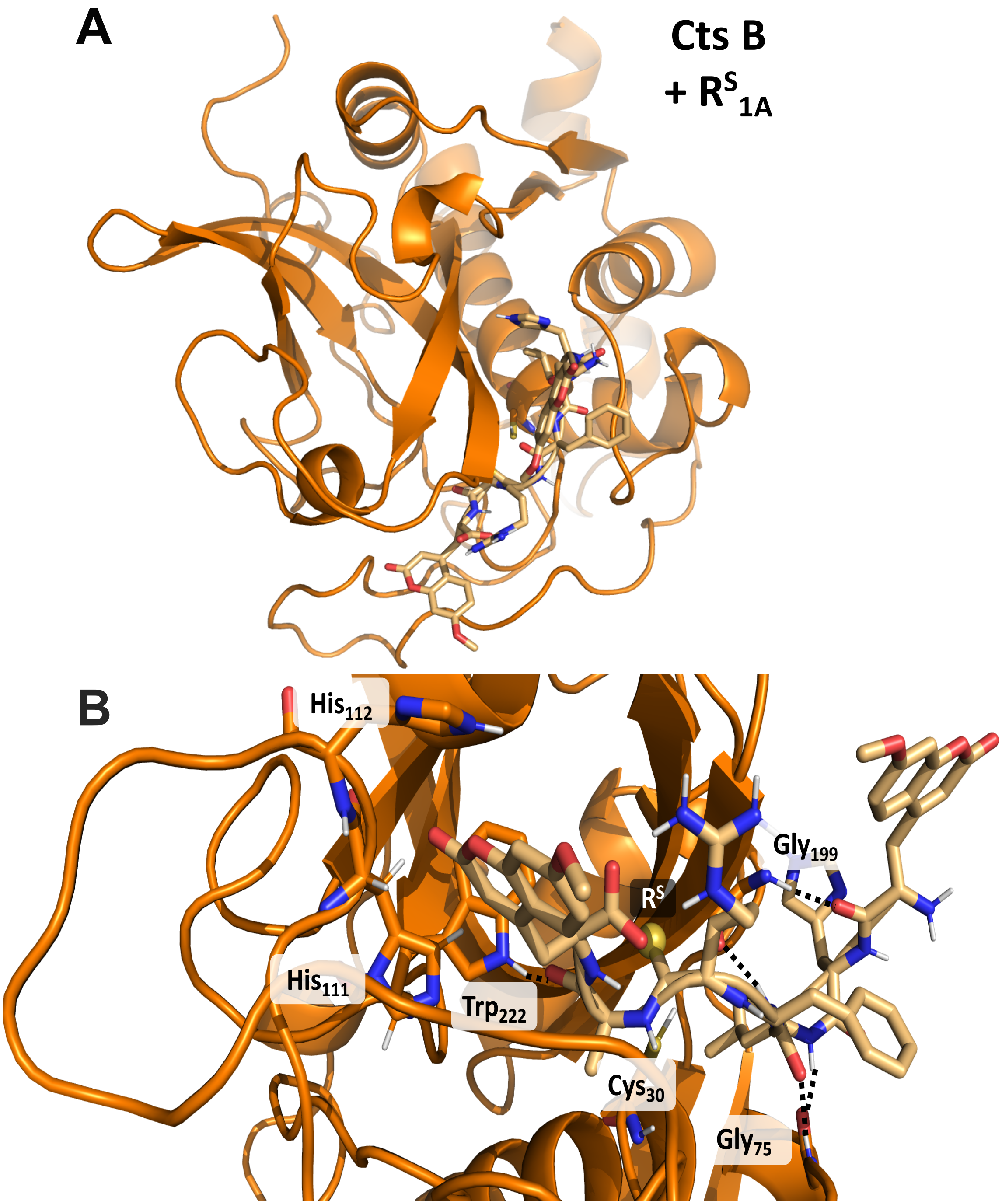

Figure S46. Structural Modeling of Cts B (orange) with R^{S}_{1A} peptide (μHLFR^{S}Aμ; light orange). (A) Cts B in complex with R^{S}_{1A} peptide. (B) Key interactions of R^{S}_{1A} peptide at the active site of Cts B. The catalytic Cys_{30} of Cts B is shown along with His_{111} and His_{112} on the occluding loop – the two key His residues for Cts B's carboxydipeptidase property. The thioamide carbonyl of R^{S}_{1A} peptide is shown as a yellow sphere. Key interactions are shown as black dashed lines.

Unsupervised Machine Learning

Score differences (deltas) for the energy features of the Rosetta full atom score function were computed between the thioamide and all-amide simulated complexes on a per-residue basis. Scores corresponding to the residue total scores of the amino acids comprising the catalytic triad and the two amino acids of the thioamide were passed as the feature matrix for machine learning. The dimensionality of this matrix was reduced to three dimensions using principal component analysis (PCA) in scikit-learn.[18] The resultant 3 PCA vectors were clustered with the KMeans clustering algorithm into four clusters. Four clusters were chosen for clustering as the silhouette heuristic was maximized at 4 clusters. **Figure 6** shows the ten peptide/protease combinations colored by cluster. **Table S26** shows the silhouette heuristic as a function of number of clusters. Finally, clustered PCA vectors were visualized with matplotlib.[19]

Table S26. Silhouette heuristics as a function of number of clusters for energetic clustering.

Number of Clusters	Silhouette Heuristic
2	0.531
3	0.547
4	0.562
5	0.470
6	0.369
7	0.317
8	0.252
9	0.095

Cell Culture & Cathepsin L Inhibition Assays in MDA-MB-231 Cells

MDA-MB-231 cells (ATCC, #HBT-26) were seeded at 1 x 10^5/well in a black, 24-well, TC-treated plate (Corning). Cells were cultured in 400 μL media per well containing DMEM (Corning), 10% Fetal Bovine Serum (FBS; Atlanta Biologicals), and 1% Penicillin/Streptomycin (Gibco) at 37ºC in 5% CO_2. After 48 hours, the cells in each well were treated with 400 μL fresh media containing either 0.5 % DMSO (sterile filtered; Sigma Aldrich), 20 μM SID 26681509 (a known Cts L inhibitor; Tocris), 10 μM E-64 (a known cell-permeable pan cathepsin inhibitor; Tocris), or 50 μM R^{S}_{1A}. Cathepsin L activity in MDA-MB-231 cells were then evaluated using ICT's Magic Red™ cathepsin-L substrate, MR-$(FR)_2$, according to the manufacturer's protocol (Immunochemistry Technologies, LLC). After incubating the cells for 24 hours in the experimental conditions, 16 μL sterile Milli-Q H_2O (for the unstained cell controls) or 16 μL 26X Magic Red staining solution was added to each well, and the 24-well plate was covered with foil for 60 minutes in a 37°C incubator with 5% CO_2. The media was removed from the cell monolayer surface and the cells were rinsed Dulbecco's phosphate-buffered saline containing Mg^{2+} and Ca^{2+} (DPBS; Corning) (2 x 500 μL DPBS/well with 1 min/rinse). The cells were then immediately visualized at 20X magnification with an Olympus CKX53 microscope with a Reflected Light Fluorescence Illuminator equipped with a green 530-550 nm exciter filler, 570 nm dichroic mirror, and a long pass 575 nm emission/barrier filter. Images were taken by Olympus EP50 camera via the Olympic EP app on an iPhone XR. Positive cells stained with Magic Red would appear red with more brightly stained vacuoles and lysosomes. All settings for the images (*e.g.* brightness, contrast, etc.) in **Figure S47** are normalized to the same values. The study was performed in two biological replicates (different batches of cells done on different days) with three technical replicates each.

Cell fluorescence from raw images was analyzed using ImageJ-Fiji. The color images were first split into RGB channels; all Red-channel images were then quantified for integrated fluorescence density across the entire images at the same threshold. There was no significant background fluorescence as can be seen in the DMSO/no Magic Red control in **Figure S47 A**. The integrated density of images from each experimental condition was converted to % Cathepsin L Activity by normalizing to the integrated density of the 0.5 % DMSO (+ Magic Red) control in each biological replicate (**Figure S47 B**). Statistical analysis using *t*-test was done in GraphPad Prism 8. Our results showed that our peptide R^{S}_{1A} (50 μM) led to reduction of Cts L activity in MDA-MB-231 by about 47%. The level of Cts L inhibition in MDA-MB-231 cells were also comparable with that of known Cts L inhibitors such as E-64 and SID 26681509. As a positive control, incubation of MDA-MB-231 cells with 10 μM E-64 for 24 hours reduced Cts L activity in MDA-MB-231 cells by 32%, which was consistent with previously done multiplex cathepsin zymography and immunoblotting experiments in the literature where 10 μM E-64 was shown to reduce Cts L activity in MDA-MB-231 cells by 30-40% after a 24-hour treatment.[20]

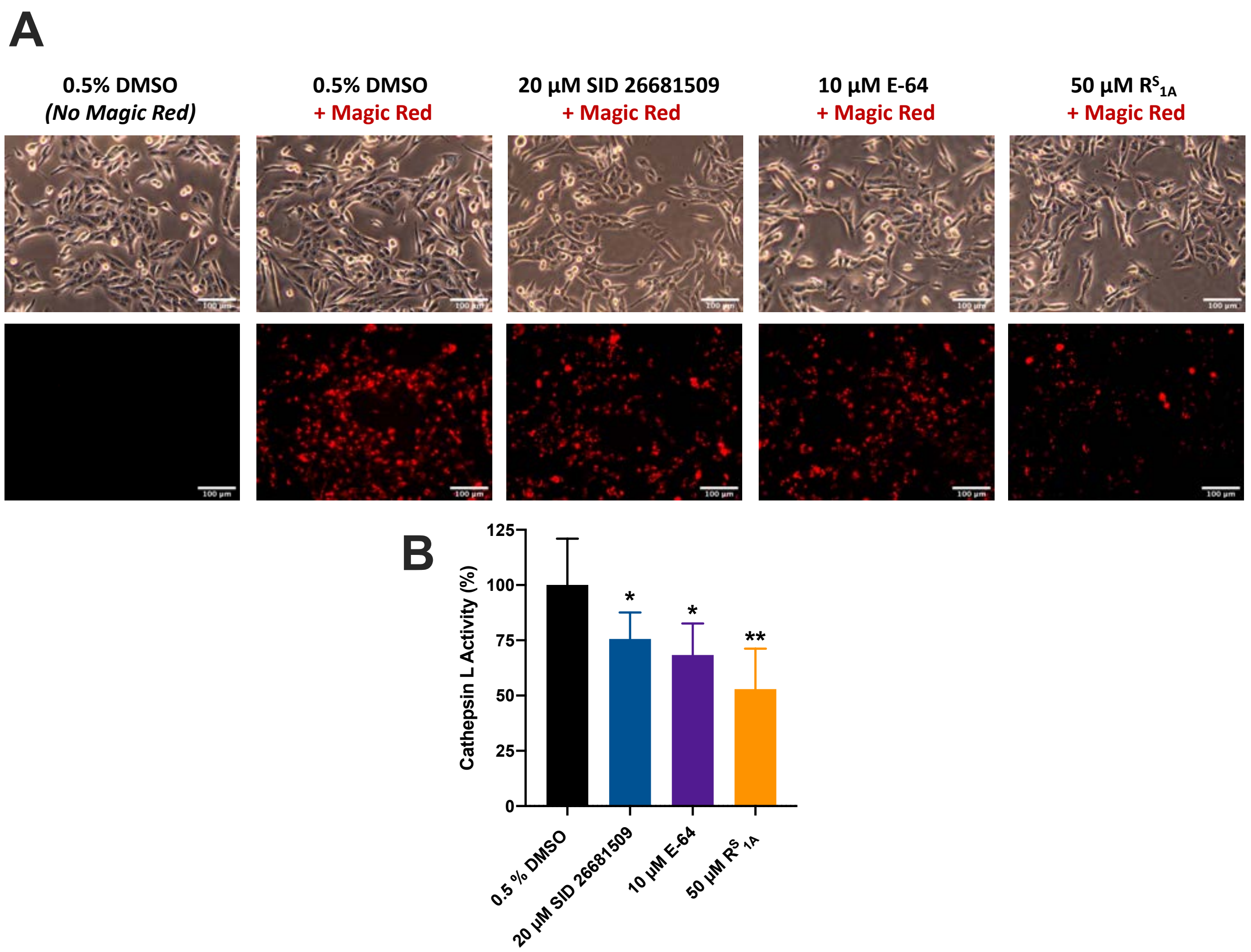

Figure S47. *In vitro* inhibition of Cathepsin L activity in human breast cancer MDA-MB-231 cells. (A) Representative images of unstained MDA-MB-231 cells and cells stained with ICT's cathepsin-L fluorogenic substrate Magic Red, MR-(FR)$_2$, taken from an Olympus CKX53 microscope at 20 X. Cts L activity was detected as the Magic Red substrate was cleaved by Cts L intracellularly. Scale bars indicate 100 µm. *Top*: Bright field images. *Bottom:* Corresponding fluorescence images of the cells. (B) Normalized Cathepsin L Activity (%) of the 0.5% DMSO control, positive controls (20 µM SID 26681509 and 10 µM SID E-64), and our peptide R^{S}_{1A}. Mean and SD from all replicates across two biological experiments in triplicates are shown. Statistical analysis using *t*-test compared to the DMSO control group was done in GraphPad Prism 8. Levels of statistical significance are expressed as * $p < 0.05$ and ** $p < 0.01$.

References

1. Y. Choe, F. Leonetti, D. C. Greenbaum, F. Lecaille, M. Bogyo, D. Brömme, J. A. Ellman and C. S. Craik, *J. Biol. Chem.*, 2006, **281**, 12824.
2. T. M. Barrett, X. S. Chen, C. Liu, S. Giannakoulias, H. a. T. Phan, J. Wang, E. K. Keenan, R. J. Karpowicz and E. J. Petersson, *ACS Chem. Biol.*, 2020, **15**, 774.
3. C. Liu, T. M. Barrett, X. Chen, J. J. Ferrie and E. J. Petersson, *ChemBioChem*, 2019, **20**, 2059.
4. D. M. Szantai-Kis, C. R. Walters, T. M. Barrett, E. M. Hoang and E. J. Petersson, *Synlett*, 2017, **28**, 1789.
5. GraphPad Prism 8 Curve Fitting Guide, https://www.graphpad.com/guides/prism/latest/curve-fitting/index.htm).

5. R. A. Copeland, *Evaluation of Enzyme Inhibitors in Drug Discovery: A Guide for Medicinal Chemists and Pharmacologists*, John Wiley & Sons, Incorporated, Somerset, United States, 2013.

7. P. P. Shah, M. C. Myers, M. P. Beavers, J. E. Purvis, H. Jing, H. J. Grieser, E. R. Sharlow, A. D. Napper, D. M. Huryn, B. S. Cooperman, A. B. Smith and S. L. Diamond, *Molecular Pharmacology*, 2008, **74**, 34.
8. J. M. Lalonde, B. Zhao, W. W. Smith, C. A. Janson, R. L. Desjarlais, T. A. Tomaszek, T. J. Carr, S. K. Thompson, H.-J. Oh, D. S. Yamashita, D. F. Veber and S. S. Abdel-Meguid, *J. Med. Chem.*, 1998, **41**, 4567.
9. S. Chaudhury, S. Lyskov and J. J. Gray, *Bioinformatics*, 2010, **26**, 689.
10. Schrödinger, *The PyMOL Molecular Graphics System, Version 1.3*, 2015
11. N. Asaad, P. A. Bethel, M. D. Coulson, J. E. Dawson, S. J. Ford, S. Gerhardt, M. Grist, G. A. Hamlin, M. J. James, E. V. Jones, G. I. Karoutchi, P. W. Kenny, A. D. Morley, K. Oldham, N. Rankine, D. Ryan, S. L. Wells, L. Wood, M. Augustin, S. Krapp, H. Simader and S. Steinbacher, *Bioorg. Med. Chem. Lett.*, 2009, **19**, 4280.
12. J. R. Somoza, H. Zhan, K. K. Bowman, L. Yu, K. D. Mortara, J. T. Palmer, J. M. Clark and M. E. Mcgrath, *Biochemistry*, 2000, **39**, 12543.
13. R. L. Desjarlais, D. S. Yamashita, H.-J. Oh, I. N. Uzinskas, K. F. Erhard, A. C. Allen, R. C. Haltiwanger, B. Zhao, W. W. Smith, S. S. Abdel-Meguid, K. D'alessio, C. A. Janson, M. S. Mcqueney, T. A. Tomaszek, M. A. Levy and D. F. Veber, *J. Am. Chem. Soc.*, 1998, **120**, 9114.
14. Y. D. Ward, D. S. Thomson, L. L. Frye, C. L. Cywin, T. Morwick, M. J. Emmanuel, R. Zindell, D. Mcneil, Y. Bekkali, M. Hrapchak, M. Deturi, K. Crane, D. White, S. Pav, Y. Wang, M.-H. Hao, C. A. Grygon, M. E. Labadia, D. M. Freeman, W. Davidson, J. L. Hopkins, M. L. Brown and D. M. Spero, *J. Med. Chem.*, 2002, **45**, 5471.
15. P. D. Greenspan, K. L. Clark, R. A. Tommasi, S. D. Cowen, L. W. Mcquire, D. L. Farley, J. H. Van Duzer, R. L. Goldberg, H. Zhou, Z. Du, J. J. Fitt, D. E. Coppa, Z. Fang, W. Macchia, L. Zhu, M. P. Capparelli, R. Goldstein, A. M. Wigg, J. R. Doughty, R. S. Bohacek and A. K. Knap, *J. Med. Chem.*, 2001, **44**, 4524.
16. Updates beta nov16, https://www.rosettacommons.org/docs/latest/Updates-beta-nov16, (accessed December 12, 2020).
17. S. Giannakoulias, S. R. Shringari, C. Liu, H. a. T. Phan, T. M. Barrett, J. J. Ferrie and E. J. Petersson, *J. Phys. Chem. B*, 2020, **124**, 8032.

18. F. Pedregosa, G. Varoquaux, A. Gramfort, V. Michel, B. Thirion, O. Grisel, M. Blondel, P. Prettenhofer, R. Weiss, V. Dubourg, J. Vanderplas, A. Passos, D. Cournapeau, M. Brucher, M. Perrot and É. Duchesnay, *J. Mach. Learn. Res.*, 2011, **12**, 2825.
19. J. D. Hunter, *Comput. Sci. Eng.*, 2007, **9**, 90.
20. W. A. Shockey, C. A. Kieslich, C. L. Wilder, V. Watson and M. O. Platt, *Cellular and Mol. Bioengineering*, 2019, **12**, 275.
